# Heteroleptic Ligation by an *endo*‐Functionalized Cage

**DOI:** 10.1002/anie.202106341

**Published:** 2021-07-13

**Authors:** Sarah C. Bete, Matthias Otte

**Affiliations:** ^1^ Institut für Anorganische Chemie University of Goettingen Tammannstraße 4 37077 Göttingen Germany

**Keywords:** dynamic combinatorial chemistry, *endo*-functionalization, facial triads, iron oxygenases, organic cages

## Abstract

A conceptual approach for the synthesis of quasi‐heteroleptic complexes with properly endo‐functionalized cages as ligands is presented. The cage ligand reported here is of a covalent organic nature, it has been synthesized via a dynamic combinatorial chemistry approach, making use of a masked amine. Inspired by enzymatic active sites, the described system bears one carboxylate and two imidazole moieties as independent ligating units through which it is able to coordinate to transition metals. Analysis of the iron(II) complex in solution and the solid state validates the structure and shows that no undesired but commonly observed dimerization process takes place. The solid‐state structure shows a five‐coordinate metal center with the carboxylate bidentately bound to iron, which makes **Fe@2** an unprecedentedly detailed structural model complex for this kind of non‐heme iron oxygenases. As, as confirmed by the crystal structure, sufficient space for other organic ligands is available, the biologically relevant ligand α‐ketoglutarate is implemented. We observe biomimetic reaction behavior towards dioxygen that opens studies investigating **Fe@2** as a functional model complex.

## Graphical abstract

A cage compound is synthesized that is *endo*‐functionalized with one carboxylic acid and two imidazole moieties. These groups can cooperate with each other to coordinate to Zn(II) and Fe(II) resulting in biomimetic quasi‐heteroleptic transition‐metal‐cage complexes. Coordination of α‐ketoglutarate to the iron complex leads to an increase of its reactivity towards dioxygen.

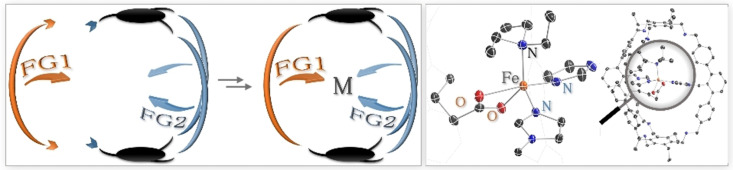

Synthetic supramolecular chemistry received wide attention during the past decades. Beside the diverse applications of supramolecular systems, such as new catalysts or as molecular machines, the synthesis of new motifs and topologies continuously steers the field towards new endeavors.[^1^, ^2^, ^3^]

A particular domain in supramolecular chemistry addresses the synthesis and application of molecular cages. Such architectures can be divided into three subclasses: 1) Cages that assemble via metal‐ligand coordination,^[4]^ 2) organic cages that assemble via supramolecular interactions such as hydrogen or halogen bonding^[5]^ and 3) organic covalent cages.^[6]^

These approaches typically lead to the thermodynamically favored products. Cage compounds that tend to have a high degree of symmetry are obtained in principally high yields. However, a lower symmetry may allow for even more specific and tunable properties.^[7]^ To selectively obtain cages of lower symmetry with the classical approach, building blocks need to be synthesized that favor social self‐sorting over narcistic self‐sorting and statistical scrambling. Unfortunately, the reaction outcome is often hard to predict. Also, examples of cage compound whose chemistry demonstrates added values occurring from lower symmetry are still rare.^[8]^

We were wondering if reducing the symmetry of a cage ligand may lead to new opportunities for cage‐type coordination chemistry. Thinking about caged metal complexes one can consider three possible scenarios:

1) The cage acts as a host for a given metal‐complex resulting in a host‐guest‐complex that has no directed bonding interaction between cage and complex,^[9]^ 2) the cage cavity is properly functionalized to form one or more directed bonds with one or more ligands of an encapsulated metal‐complex^[10]^ or 3) the cage is endo‐functionalized, enabling its direct engagement as a (multidentate) ligand to a metal.^[11]^ In this case the metal‐coordinating atoms belong to the cage. Considering covalent organic systems, well known multidentate ligand motifs such as tren or trialkanolamine units have been incorporated in cages as chelating units.^[12]^

Imidazole coordination is of great importance in enzymatic active copper sites.^[13]^ There, the coordinating amino acid residues mainly belong to different regions of the poly peptide sequence. This results in pseudo‐monodentate ligation by the imidazole groups. Reinaud mimicked such motives for coordination to copper and other metals via rim‐functionalized calixarenes that possess imidazole or imidazole and phenolate ligands.^[14]^ Yaghi reported imidazole‐functionalized MOFs for copper‐coordination.^[15]^

Inspired by these findings we transferred this concept to molecular organic cages and synthesized an imidazole‐functionalized aza‐cyclophane. We demonstrated that this cage can act as a homoleptic tridentate ligand for copper (I) resulting in **Cu@1** (Figure 1 top).^[16]^ Although **1** can be regarded as multidentate ligand, the coordinating units are connected to different edges of the cage reminiscent of different poly peptide regions of an enzyme.

**Figure 1:**
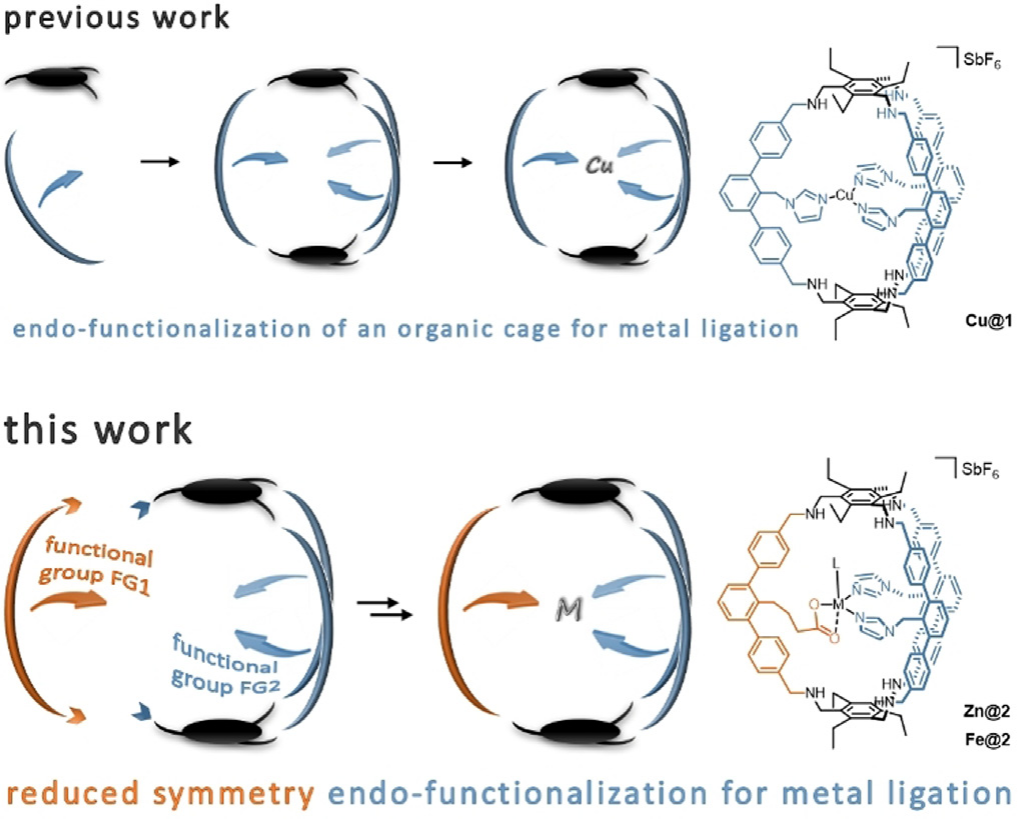
Schematic depiction of the synthesis of the endo‐functionalized aza‐cyclophanes **1** and **2** and their corresponding metal complexes **Cu@1**, **Zn@2** and **Fe@2**.

Intrigued by **Cu@1** we were wondering if one could design an advanced generation of aza‐cyclophanes that create a heteroleptic ligand environment. As many enzymatic active sites employ coordination of two imidazole and one carboxylate moiety to a metal centre, we chose to substitute one of the endohedral imidazole groups in **1** for a carboxylic acid. This coordination environment, the so‐called 2‐his‐1‐carboxylate facial triad, is in particular well known for iron.^[17]^ Therefore, we figured **Fe@2** (Figure 1 bottom) as a suitable synthetic goal.

The synthesis of **2** occurs in a convergent fashion. As a modification of the synthesis of **1**, the imidazole‐functionalized dialdehyde **3** is reacted with an amine precursor, in which one amine group is masked as an azide group (**4**, Scheme 1 top). With a combined yield of 59 % this reductive amination, followed by a Staudinger reaction, leads to the formation of macrocycle **5**.[^16^, ^18^] In principle, **5** may be reacted with any suitable derivative of **3** where the imidazole unit might be substituted for a different moiety. This has the potential to lead to a large variety of endo‐(Im)_2_(X)_1_‐functionalized organic cage ligands. Here, we chose for carboxylic acid **6** which could be obtained in three steps starting from known **7** (Scheme 1 bottom). Reaction of **7** with methyl acrylate in the presence of titanocene(III)chloride that is generated *in situ* from Cp_2_TiCl_2_ and zinc powder gave **8** in 16 % yield.^[19]^ Suzuki coupling with 4‐formylphenylboronic acid gave **9** (67 %) that was hydrolyzed to desired **6** (93 %).

**Scheme 1:**
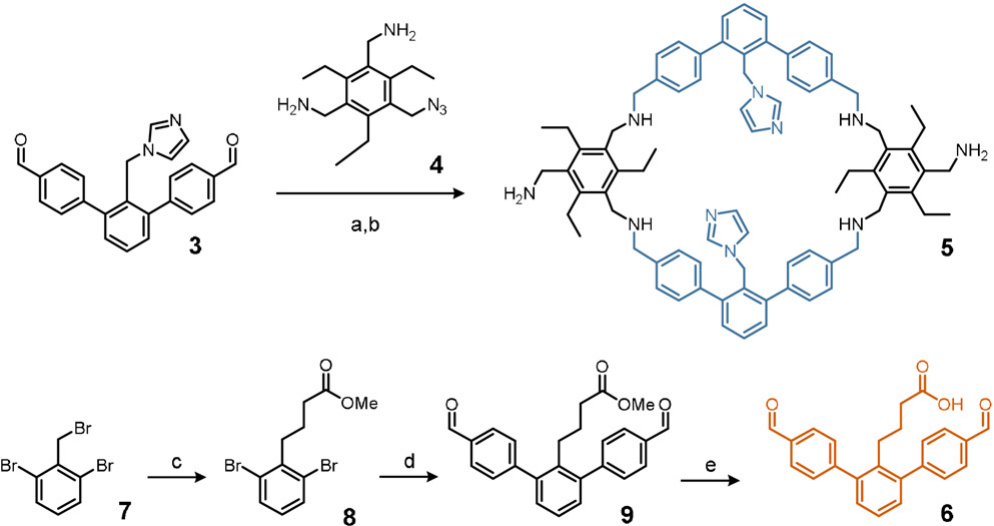
Synthesis of cage building blocks **5** and **6**: a) DCM, MeOH, rt, 48 h then NaBH_4_, aq. work‐up; b) excess PPh_3_, THF, MeOH and H_2_O (59 % over 2 steps) c) Cp_2_TiCl_2_, Zn, rt, THF, 2 h then **7** and methyl acrylate (16 %); d) 5 mol % Pd(PPh_3_)_4_, 3 equiv. 4‐formylphenylboronic acid, Na_2_CO_3_, toluene, EtOH, H_2_O, 100 °C, 72 h, (67 %); e) 5 equiv. pTsOH, MeCN, H_2_O, 100 °C, 72 h (93 %).

The reductive amination with **5** and **6** gives cage **2** almost quantitatively according to crude NMR but with a yield of 78 % after purification (Scheme 2). Notably, the reaction towards **2** via mixing of the corresponding triamine with **3** and **6** in a 2:2:1 ratio gives statistical product mixture out of which **2** could not be isolated. Tuning the selectivity by the use of templating effects was also not successful (see S6 for details, figures S9/S10). **2** was characterized via ESI‐MS, ^1^H, ^1^H DOSY, ^13^C NMR and IR spectroscopy. The ESI‐MS reveals signals with *m*/*z* values of 1507.8832, 754.4474 and 503.3011 corresponding to **2**+H^+^, **2**+2 H^+^ and **2**+3H^+^. In the ^1^H NMR spectrum, three singlets are detected at chemical shifts of 6.74, 6.45 and 6.21 ppm that originate from either one proton of the two imidazole units, showing their chemical equivalence on the NMR timescale (These signals as well as the one for the methylene linker are enlightened in blue, see Figure 2 top). The multiplets at 2.37, 1.67 and 1.24 ppm, enlightened in orange, correspond to the propylene linker between the cage backbone and the carboxylic acid group. The presence of the carboxylic acid is further confirmed by a signal at 174.05 ppm in the ^13^C NMR and a signal at 1710 cm^−1^ in the IR spectrum (see figures S14/S20). Figure 2 bottom shows the ^1^H DOSY NMR of **2**. Despite the NMR solvent signal all other observed signals belong to a species with a diffusion coefficient of 5.6×10^−10^ m^2^ s^−1^. Using the Stokes‐Einstein equation for spherically shaped molecules, this diffusion coefficient translates to a hydrodynamic radius of 9.4 Å which is in good agreement with the one previously found for **1**.^[16]^

**Figure 2:**
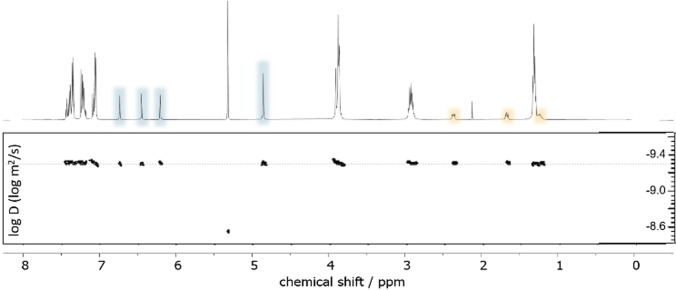
^1^H (top) and ^1^H DOSY NMR (bottom) spectra of **2** in CD_2_Cl_2_.

**Scheme 2:**
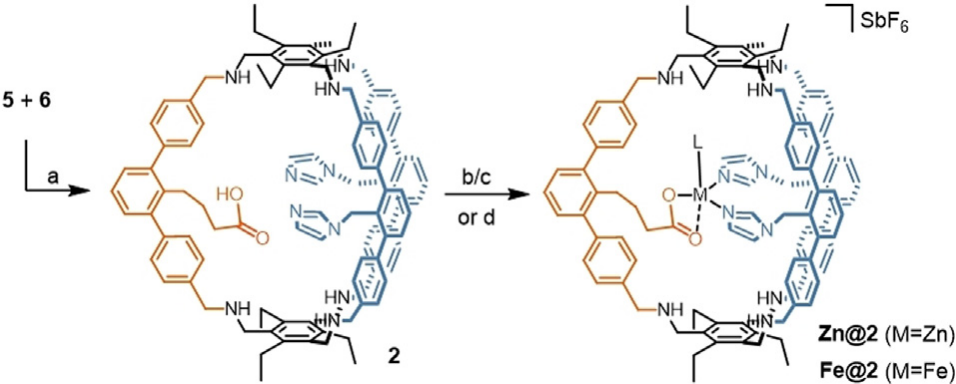
Synthesis of cage **2** and its reaction with Zn^II^ and Fe^II^ to **Zn@2** and **Fe@2**: a) DCM, MeOH, rt, 48 h then NaBH_4_, aq. work‐up (78 %); b) 1.1 equiv. [Zn(MeCN)_4_](SbF_6_)_2,_ [D_8_]THF, 1 h, then NEt_3_; c) 1.1 equiv. [Fe(MeCN)_6_](SbF_6_)_2_, [D_8_]THF, 1 h, then NEt_3_ d) 0.8 equiv. [Fe(MeCN)_6_](SbF_6_)_2_, MeCN, rt, 14 h, then NEt_3_. L=labile coordination site.

With cage **2** in hand, we studied its ability to serve as a quasi‐heteroleptic chelating ligand for transition metals. Due to their biological relevance, we chose for iron(II) and the diamagnetic zinc analogue[^17^, ^20^] (Scheme 2). Quantitative conversion is observed by reacting **2** with 1.1 equiv. of the divalent metal precursors [Zn(MeCN)_4_](SbF_6_)_2_ and [Fe(MeCN)_6_](SbF_6_)_2_ in [D_8_]THF and subsequent treatment with triethylamine. To ensure absence of metal salt traces in the follow up chemistry, a modified procedure with understoichiometric use and separation of **Fe@2** from the free ligand is employed in the case of iron. The IR absorption at 1710 cm^−1^ that can be assigned to the carbonyl stretching of the carboxylate of **2** is not found anymore in any case and is presumably shifted into the finger print area of the spectrum (see ESI). Such behavior is indicative for carboxylate coordination to a metal. By high resolution ESI‐MS, [C_102_H_109_N_10_O_2_Zn] (**Zn@2**) and [C_102_H_109_N_10_O_2_Fe] (**Fe@2**) could be confirmed (see figures S27/S35).

The complexes were analyzed by ^1^H, ^1^H DOSY and ^13^C NMR spectroscopy in [D_8_]THF. Comparison of the ^1^H and ^13^C NMR spectra of **2** and **Zn@2** confirms coordination of the functional residues as we observe a shift for both the imidazole signals and the propylene linker (see ESI for NMR spectra). In the ^13^C NMR of **Zn@2** we see broadening of the propylene linker signals, the carboxyl carbon resonance could not be detected. We attribute this behavior to a limited flexibility upon zinc coordination. ^1^H DOSY NMR reveals an enhancement of the hydrodynamic radius to 10.6 Å, which is expected due to the ionic nature of **Zn@2**.

The ^1^H NMR spectrum of **Fe@2** (see Figure 3) shows multiple broad resonances mostly part of the chemical shift region expected for diamagnetic compounds, but apart from the ones originating from solvent molecules only signals with *T*_1_ times with less than 200 ms are detected. Three signals are observed with a shift typical for paramagnetic substances, at 72, 44 and 20 ppm, but due to their decay times could not be correlated by DOSY NMR or any 2d methods. For all other signals, the same diffusion coefficient as in the case of **Zn@2** (*D*=4.4×10^−10^ m^2^ s^−1^) is found via DOSY NMR (Figure 3 b). We would like to stress that no indication was found that **2** may coordinate in an undesired carboxylate bridging fashion, in particular avoiding the formation of **Fe_2_@2_2_** or any other undesired dimerization products such as **Fe@2_2_** that are often observed in iron complexes with comparable first coordination sphere.^[21]^

**Figure 3:**
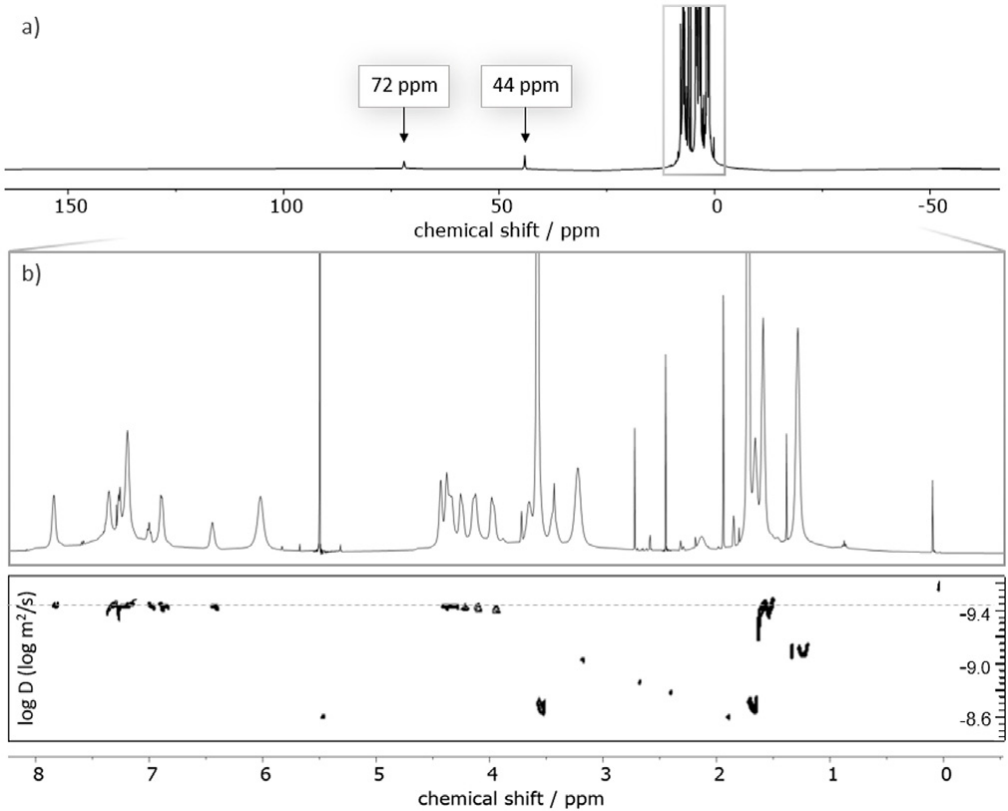
a) ^1^H and b) ^1^H DOSY NMR spectra of **Fe@2** in [D_8_]THF.

From a solution of **Fe@2** in acetonitrile/NEt_3_ the corresponding amine complex crystallizes over time, giving crystals suitable for *x*‐ray diffraction analysis. Figure 4 depicts the structure of **Fe(NEt_3_)@2** in the solid state confirming that **2** is heteroleptically ligating the iron center. The Fe−N bond lengths for the two coordinating imidazole units are 2.07 Å and 2.10 Å while the Fe−N bond for the additionally coordinating external NEt_3_ ligand is 2.15 Å (Figure 4 c). The Fe‐O distances are 2.06 and 2.29 Å, while the C‐O distances within the carboxylate are 1.28 and 1.25 Å, showing that at least in the solid state it acts as a bidentate ligand to iron. Although such asymmetric bidentate carboxylate coordination to iron bearing two imidazole ligands is also found in the active sites of enzymes such as naphthalene dioxygenase (NDO),^[22]^ we are not aware of a synthetic complex that shows this coordination motive. As such **Fe@2** is a nice addition to the toolbox of 2‐histidine‐1‐carboxylate model‐complexes.^[23]^

**Figure 4:**
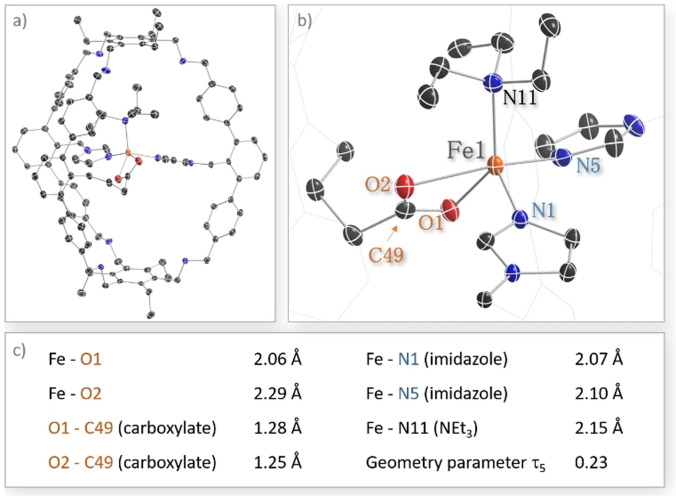
a) Molecular structure of **Fe(NEt_3_)@2** in the solid state. Hydrogen atoms, solvent molecules and the SbF_6_ anion are omitted for clarity, b) enlarged view of the coordination site and c) selected bond lengths in **Fe(NEt_3_)@2**.

The structure of **Fe(NEt_3_)@2** nicely shows that **2** is well‐designed to act as a multidentate ligand while offering sufficient space for the coordination of external compounds. Similar to the behavior observed in solution we see monomer formation and no undesired dimerization in the solid state. To the best of our knowledge, this is the first heteroleptic metal complex of a cage in which the metal is ligated by different independent functional groups that are covalently connected to the ligand backbone.

In agreement with the reaction mechanisms found for substrate oxidation in iron oxygenases, where O_2_ binding is prevented by the presence of an external water ligand,^[24]^ **Fe@2** does not readily react with dioxygen (see Figure S37). Instead, oxygen activation in iron oxygenases only takes place after cofactor and/or substrate binding, for instance after binding of α‐ketoglutarate (αKG) in α‐ketoglutarate‐dependent iron oxygenases. Inspired by this, we examined the reactivity of **Fe@2** towards dioxygen in the presence of αKG. Coordination to **Fe@2** is confirmed by a spectral change in NMR and via UV/Vis spectroscopy, where upon addition of αKG we observe a feature at 485 nm that is in the range of Fe^II^ t_2g_ to αKG π* MLCT transitions observed for αKG‐bound enzymatic Fe^II^ sites.^[25]^ Typically, this transition is found at 520 nm, for example in TauD.^[26]^ Interestingly, a very well matching absorption at 483 nm is observed in FIH, where the increase is attributed to the presence of additional H‐bonds stabilizing the bound αKG carboxyl group which then leads to lower kinetic barrier for peroxo bridge formation.[^27^, ^28^]

As an additional indication of the αKG complex being formed, [C_102_H_109_N_10_O_2_Fe(C_5_H_5_O_5_)] is found in high resolution ESI‐MS, but besides further adducts.

Warming up a cooled solution of the resulting αKG‐bound complex in the presence of O_2_ leads to a direct spectral change in UV/Vis even at low temperatures (pink to magenta, Figure 5, NMR Spectra see Figure S38), proving the increase of reactivity towards dioxygen in the presence of the paradigmatic co‐substrate αKG. A detailed analysis of the formed species and their capability of oxidizing organic substrates especially regarding the involvement of H‐bonding interactions to the ligand backbone will be part of future investigations, but with these early findings we were already able to show that **Fe@2** serves as a functional mimic for non‐heme iron oxygenases.

**Figure 5:**
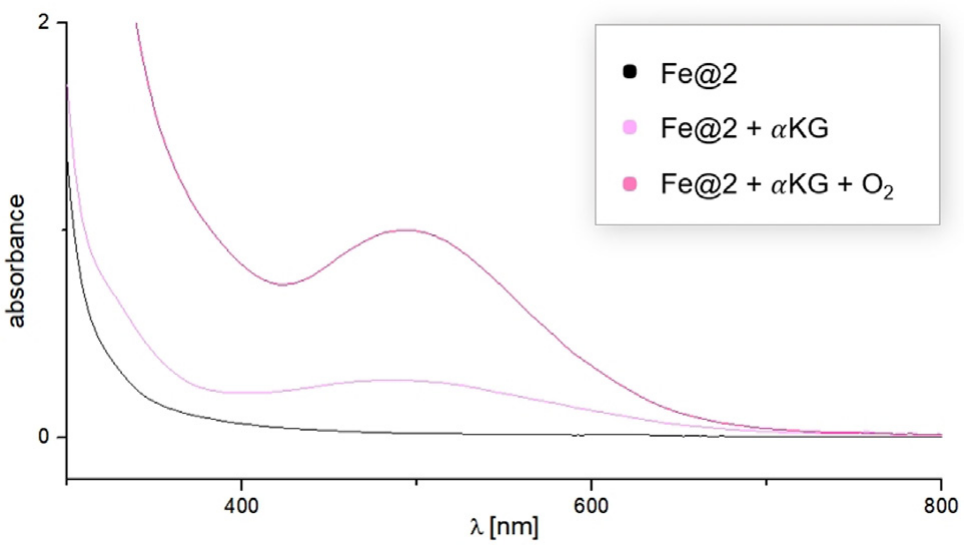
UV/Vis absorption spectra of **Fe@2** (black), after addition of αKG at rt (pink) and after warming up from −80 °C under O_2_ atmosphere (magenta).

In summary, we have reported the stepwise synthesis of the endo‐functionalized cage ligand **2. 2** is capable of coordinating zinc(II) and iron(II), resulting in the formation of the quasi‐heteroleptic cage‐complexes **Zn@2** and **Fe@2**. Solution and solid‐state investigation show that these are monomeric structures and no undesired dimers are obtained. X‐ray analysis shows that additional coordination of NEt_3_ to **Fe@2** is possible and that the carboxylate unit coordinates in an unsymmetrically bidentate fashion reminiscent of for example the active site of naphthalene dioxygenase. Finally, we found **Fe@2** not only being a structural but also a functional model of enzymatic active sites as its reactivity towards oxygen is strongly enhanced in the presence of bound α‐ketoglutarate. Overall, the reported concept of applying cages of lowered symmetry as ligands may lead to huge varieties of this class of metal complexes, whose properties could selectively be tuned by derivatization of the used building blocks. The accessibility of the introduced compound class can especially be of interest for the design of biomimetic complexes as many metalloproteins feature a heteroleptic coordination environment within the active site pocket.

## Conflict of interest

The authors declare no conflict of interest.

## Supporting information

As a service to our authors and readers, this journal provides supporting information supplied by the authors. Such materials are peer reviewed and may be re‐organized for online delivery, but are not copy‐edited or typeset. Technical support issues arising from supporting information (other than missing files) should be addressed to the authors.

Supporting Information

Supporting Information

Supporting Information
