# Supplementary material for: Heteroleptic Ligation by an endo‐Functionalized Cage
Source: Angew Chem Int Ed Engl. 2021 Jul 13;60(34):18582–6. doi: 10.1002/anie.202106341 (PMC8456844; doi:10.1002/anie.202106341)

checkCIF/PLATON report

Structure factors have been supplied for datablock(s) SB_191220_2_MO

THIS REPORT IS FOR GUIDANCE ONLY. IF USED AS PART OF A REVIEW PROCEDURE FOR PUBLICATION, IT SHOULD NOT REPLACE THE EXPERTISE OF AN EXPERIENCED CRYSTALLOGRAPHIC REFEREE.

No syntax errors found. CIF dictionary Interpreting this report

Datablock: SB_191220_2_MO

Bond precision: C-C = 0.0076 A Wavelength=0.71073

Cell:	a=16.5958(4)	b=18.6880(5)	c=19.4938(5)
	alpha=64.040(1)	beta=76.155(1)	gamma=86.568(2)
Temperature:	100 K		

	Calculated	Reported
Volume	5270.9(2)	5270.9(2)
Space group	P -1	P -1
Hall group	-P 1	-P 1
Moiety formula	C108 H124 Fe N11 O2, F6 Sb, 4(C2 H3 N)	?
Sum formula	C116 H136 F6 Fe N15 O2 Sb	C116 H136 F6 Fe N15 O2 Sb
Mr	2064.02	2063.99
Dx,g cm-3	1.301	1.300
Z	2	2
Mu (mm-1)	0.463	0.463
F000	2168.0	2168.0
F000’	2168.33	
h,k,lmax	20,23,24	20,23,24
Nref	20947	20848
Tmin,Tmax	0.936,0.963	0.900,0.960
Tmin’	0.915	

Correction method= # Reported T Limits: Tmin=0.900 Tmax=0.960
AbsCorr = MULTI-SCAN

Data completeness= 0.995 Theta(max)= 26.100

R(reflections)= 0.0815(16716) wR2(reflections)= 0.1665(20848)

S = 1.158 Npar= 1307

The following ALERTS were generated. Each ALERT has the format

test-name_ALERT_alert-type_alert-level.

Click on the hyperlinks for more details of the test.

Alert level C

```
RINTA01_ALERT_3_C  The value of Rint is greater than 0.12
            Rint given   0.145
PLAT020_ALERT_3_C The Value of Rint is Greater Than 0.12 .........      0.145 Report
PLAT222_ALERT_3_C NonSolvent Resd 1  H   Uiso(max)/Uiso(min) Range       10.0 Ratio
PLAT244_ALERT_4_C Low    'Solvent' Ueq as Compared to Neighbors of        Sb1 Check
PLAT244_ALERT_4_C Low    'Solvent' Ueq as Compared to Neighbors of       C111 Check
PLAT250_ALERT_2_C Large U3/U1 Ratio for Average U(i,j) Tensor ....        3.0 Note
PLAT420_ALERT_2_C D-H Without Acceptor       N3       --H803     .     Please Check
PLAT420_ALERT_2_C D-H Without Acceptor       N4       --H804     .     Please Check
PLAT420_ALERT_2_C D-H Without Acceptor       N10      --H810     .     Please Check
PLAT906_ALERT_3_C Large K Value in the Analysis of Variance ......     10.949 Check
PLAT906_ALERT_3_C Large K Value in the Analysis of Variance ......      2.417 Check
PLAT910_ALERT_3_C Missing # of FCF Reflection(s) Below Theta(Min).          7 Note
```

Alert level G

```
PLAT002_ALERT_2_G Number of Distance or Angle Restraints on AtSite         12 Note
PLAT083_ALERT_2_G SHELXL Second Parameter in WGHT  Unusually Large      26.69 Why ?
PLAT172_ALERT_4_G The CIF-Embedded .res File Contains DFIX Records          6 Report
PLAT720_ALERT_4_G Number of Unusual/Non-Standard Labels ..........          1 Note
PLAT790_ALERT_4_G Centre of Gravity not Within Unit Cell: Resd.  #          6 Note
              C2 H3 N
PLAT794_ALERT_5_G Tentative Bond Valency for Sb1       (V)       .       5.13 Info
PLAT794_ALERT_5_G Tentative Bond Valency for Fe1       (II)      .       1.85 Info
PLAT860_ALERT_3_G Number of Least-Squares Restraints .............          6 Note
PLAT883_ALERT_1_G No Info/Value for _atom_sites_solution_primary .     Please Do !
PLAT912_ALERT_4_G Missing # of FCF Reflections Above STh/L=  0.600         88 Note
PLAT978_ALERT_2_G Number C-C Bonds with Positive Residual Density.          5 Info
```

---

0 **ALERT level A** = Most likely a serious problem - resolve or explain
0 **ALERT level B** = A potentially serious problem, consider carefully
12 **ALERT level C** = Check. Ensure it is not caused by an omission or oversight
11 **ALERT level G** = General information/check it is not something unexpected

1 ALERT type 1 CIF construction/syntax error, inconsistent or missing data
7 ALERT type 2 Indicator that the structure model may be wrong or deficient
7 ALERT type 3 Indicator that the structure quality may be low
6 ALERT type 4 Improvement, methodology, query or suggestion
2 ALERT type 5 Informative message, check

It is advisable to attempt to resolve as many as possible of the alerts in all categories. Often the minor alerts point to easily fixed oversights, errors and omissions in your CIF or refinement strategy, so attention to these fine details can be worthwhile. In order to resolve some of the more serious problems it may be necessary to carry out additional measurements or structure refinements. However, the purpose of your study may justify the reported deviations and the more serious of these should normally be commented upon in the discussion or experimental section of a paper or in the "special_details" fields of the CIF. checkCIF was carefully designed to identify outliers and unusual parameters, but every test has its limitations and alerts that are not important in a particular case may appear. Conversely, the absence of alerts does not guarantee there are no aspects of the results needing attention. It is up to the individual to critically assess their own results and, if necessary, seek expert advice.

Publication of your CIF in IUCr journals

A basic structural check has been run on your CIF. These basic checks will be run on all CIFs submitted for publication in IUCr journals (*Acta Crystallographica*, *Journal of Applied Crystallography*, *Journal of Synchrotron Radiation*); however, if you intend to submit to *Acta Crystallographica Section C* or *E* or *IUCrData*, you should make sure that full publication checks are run on the final version of your CIF prior to submission.

Publication of your CIF in other journals

Please refer to the *Notes for Authors* of the relevant journal for any special instructions relating to CIF submission.

PLATON version of 05/12/2020; check.def file version of 05/12/2020

Datablock SB_191220_2_MO - ellipsoid plot

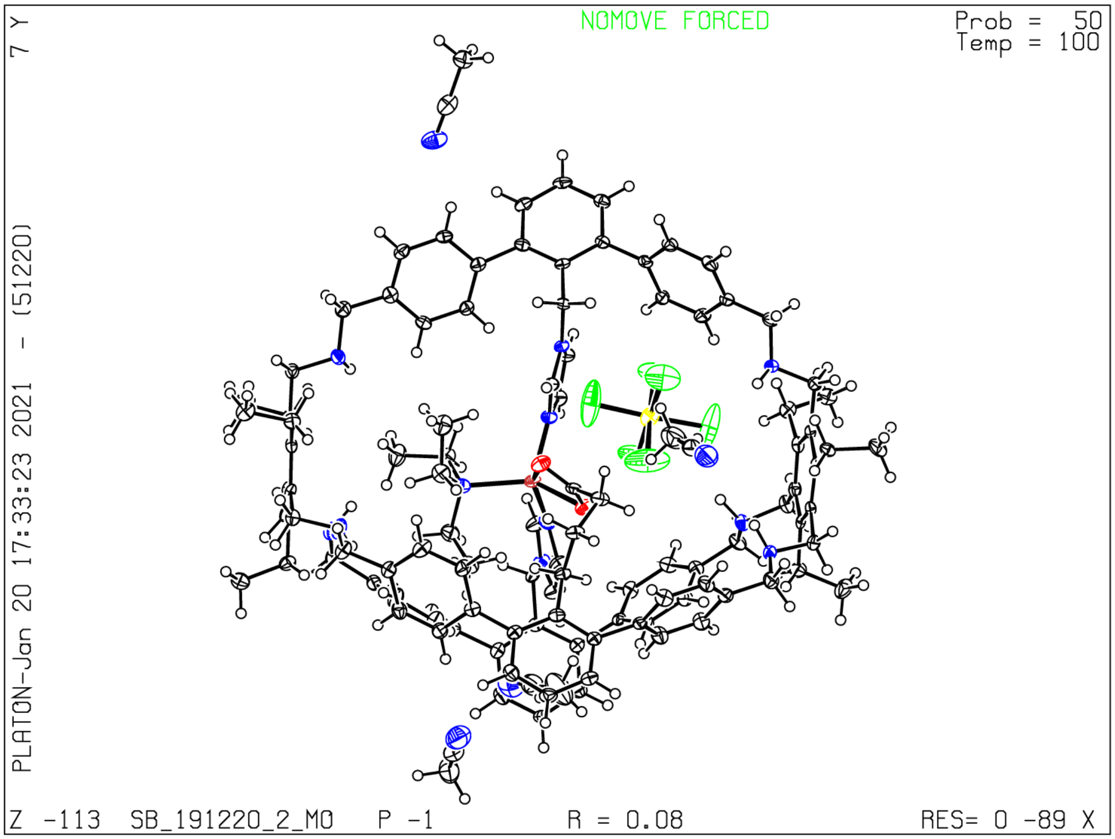

Supplement: Supplementary file 1 — Supporting Information [file ANIE-60-18582-s003.zip › checkcif_Fe(NEt3)_at_2.pdf]
