# Supplementary material for: Heteroleptic Ligation by an endo‐Functionalized Cage
Source: Angew Chem Int Ed Engl. 2021 Jul 13;60(34):18582–6. doi: 10.1002/anie.202106341 (PMC8456844; doi:10.1002/anie.202106341)
Supplement: Supplementary file 2 — Supporting Information [file ANIE-60-18582-s001.pdf]

Angewandte Chemie
Eine Zeitschrift der Gesellschaft Deutscher Chemiker

Supporting Information

Heteroleptic Ligation by an *endo*-Functionalized Cage

*Sarah C. Bete and Matthias Otte**

anie_202106341_sm_miscellaneous_information.pdf
anie_202106341_sm_Video.mp4

Table of Contents

Experimental Procedures 2
General information 2
Synthesis of 5 2
Synthesis of 8 3
Synthesis of 9 3
Synthesis of 6 4
Synthesis of 2 4
Statistical approaches towards 2 5
Method 1 5
Methods 2 and 3 5
Synthesis of Zn@2 6
Synthesis of Fe@2 7
NMR-monitored Reaction of Fe@2 with dioxygen 7
NMR-monitored Reaction of Fe@2 and α-ketoglutarate with dioxygen 7
UV/Vis-monitored Reaction of Fe@2 and α-ketoglutarate with dioxygen 8
X-ray Single-Crystal Structure Analysis 31
Crystallographic Details 31
X-ray Single-Crystal Structure Analysis of Fe@2 31
References 51
Author Contributions 51

Experimental Procedures

General information

All reactions involving air- or moisture sensitive compounds were carried out under nitrogen using either standard Schlenk and vacuum line techniques or in UNILAB Glovebox from MBraun. All reagents were purchased from commercial sources and used as received unless otherwise stated. Methanol was distilled over calcium hydride and stored over molecular sieves under argon. Tetrahydrofurane (THF), dichloromethane (DCM), acetonitrile and benzene were taken from a MBRAUN MB SPS-800 solvent purification system. Deuterated DCM (CD_2Cl_2), benzene (C_6D_6), tetrahydrofurane (THF-d_8) and Methanol were degassed using the freeze-thaw-pump cycles and subsequently stored over molecular sieves. Triethylamine was dried over KOH and CaH_2, destilled and afterwards overcondensed in vacuo. Compounds **3**[1] and **4**[2] and **7**[3] and **10**[4] , were synthesized as described in literature. $[Zn(MeCN)_4](SbF_6)_2$ and $[Fe(MeCN)_6](SbF_6)_2$ were prepared following the literature procedures for the corresponding tetrafluoroborate salts.[5] Dioxygen was dried by storing in a cooling coil at -80 °C for 2 hours prior to use.

^1H and ^{13}C NMR spectra were recorded on a Bruker Avance 300, Avance 400 or Avance 500 spectrometer at 25 °C. ^1H and ^{13}C NMR chemical shifts are reported in ppm relative to TMS using the residual solvent resonance as internal standard. Infrared spectra were recorded using an ALPHA Platinum-ATR FTIR spectrometer from Bruker. MS measurements were performed at the analytic laboratory of the chemistry department. Hydrodynamic radii were calculated using the Stokes Einstein equation using the dynamic viscosity.[6]

Synthesis of 5

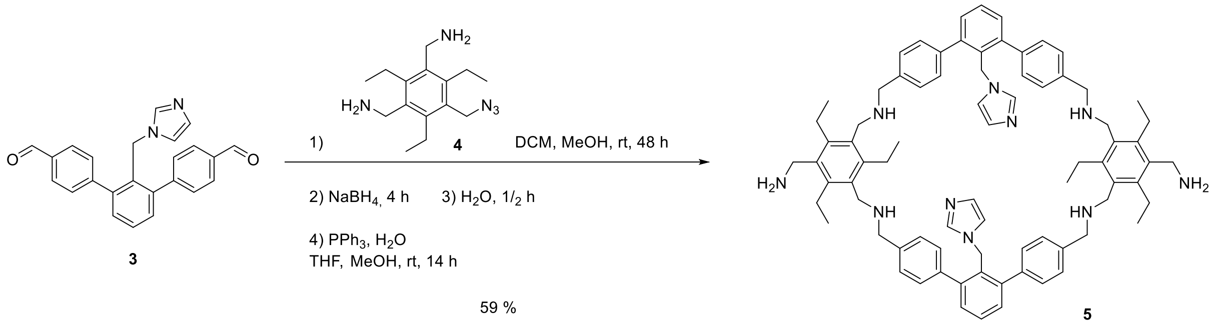

Scheme S1. Synthesis of **5**.

4 (603 mg, 2.19 mmol, 1.00 eq) and **3** (806 mg, 2.19 mmol, 1.00 eq) were placed in a Schlenk flask under argon. Dry dichloromethane (120 mL) and dry methanol (12 mL) were added and the reaction mixture was stirred for 48 h at room temperature. $NaBH_4$ (166 mg, 4.38 mmol, 2.00 eq) was added and the resulting mixture was stirred for 4 h at room temperature. After addition of water (50 ml) the mixture was stirred for 10 minutes. The phases were separated and the aqueous phase was extracted with dichloromethane (2 x 25 ml). The organic phase was washed with water, dried over magnesium sulfate and the solvent was removed under reduced pressure. The residue was dissolved in THF (80 ml) and MeOH (20 ml) and Triphenylphosphine (1.26 g, 4.82 mmol, 2.20 eq) was added. The mixture was allowed to stir for 14 h in a flask with a gas outlet. Water (100 ml) was added and the resulting suspension was filtered. The residue was washed with water (20 ml), acetonitrile (2 x 30 ml) and extracted with a mixture of dichloromethane and methanol (10:1, 50 ml). The product was obtained as a white solid (754 mg, 0.646 mmol, 59%).

^1H NMR (300 MHz, CD_2Cl_2) δ 7.41 (d, *J* = 7.8 Hz, 9H), 7.36 (dd, *J* = 8.4, 6.7 Hz, 2H), 7.25 (d, *J* = 7.4 Hz, 4H), 7.16 (d, *J* = 7.7 Hz, 9H), 6.19 (t, *J* = 1.2 Hz, 2H), 5.00 (s, 4H), 3.94 (s, 9H), 3.85 (s, 4H), 3.77 (s, 8H), 2.86 (q, *J* = 7.4 Hz, 8H), 2.70 (q, *J* = 7.4 Hz, 5H), 1.46 (s, 14H), 1.25 (t, *J* = 7.4 Hz, 13H), 1.10 (t, *J* = 7.4 Hz, 6H).

^{13}C NMR (101 MHz, CD_2Cl_2) δ 144.16, 142.45, 141.95, 140.60, 139.69, 137.54, 136.99, 134.72, 131.15, 130.39, 129.31, 128.64, 128.58, 128.52, 118.85, 54.86, 47.91, 45.41, 40.03, 22.95, 22.61, 17.21, 17.00.

IR (ATR-FTIR): 2960, 2925, 2900, 2865, 1585, 1565, 1500, 1450, 1400, 1385, 1370, 1355, 1275, 1225, 1190, 1105, 1070, 1045, 1020, 945, 1020, 945, 905, 850, 805, 775, 725, 700, 660, 615, 635.

Exact mass ESI MS: $C_{78}H_{90}N_{10}$ $[M+H]^+$ calculated: 1167.7423 found: 1167.7405.

Synthesis of 8

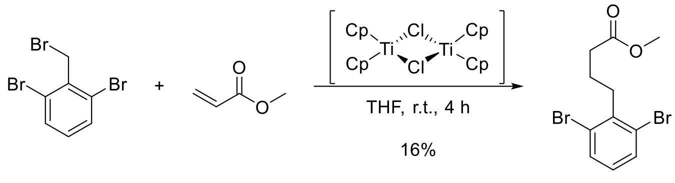

7 8

Scheme S2. Synthesis of **8**.

Titanocendichloride (4,98 g, 20.0 mmol, 2.00 eq) and Zink (6.54 g, 100 mmol, 10.0 eq) were placed in a Schlenk flask. Dry THF (300 ml) was added and the mixture was stirred for 1 hour. Over the course of 2 hours via a filter canula the green solution was transferred dropwise into a solution of **7** (3.29 g, 10.0 mmol, 1.00 eq) and methyl acrylate (0,906 ml, 10.0 mmol, 1.00 eq) in dry THF (150 ml). The resulting mixture was stirred for 2 hours and afterwards decomposed with H_2SO_4 (10% aqueous solution, 150 ml). The mixture was extracted with diethyl ether (3 x 100 ml) and the combined organic phases were washed with water (2 x 100 ml) and brine (2 x 50 ml) and dried over magnesium sulfate. After removal of the solvent the crude product was extracted with Hexane (50 °C, 500 ml) and purified via column chromatography (Hexanes (R_F=0) -> Hexane/EtOAc 199:1 (R_F=0.1)). **8** was obtained as a yellow oil (550 mg, 1.64 mmol, 16%).

^{1}H NMR (400 MHz, CDCl3) δ 7.52 (d, J = 7.9 Hz, 2H), 6.93 (t, J = 8.0 Hz, 1H), 3.71 (s, 3H), 3.11 – 3.00 (m, 2H), 2.48 (t, J = 7.5 Hz, 2H), 2.10 – 1.88 (m, 2H).

^{13}C NMR (101 MHz, $CDCl_3$) δ 173.75, 140.38, 132.45, 128.81, 125.35, 51.74, 36.22, 33.87, 23.62.

IR (ATR-FTIR): 2950, 2875, 2845, 1735, 1575, 1550, 1455, 1430, 1365, 1340, 1260, 1195, 1170, 1155, 1124, 1075, 1040, 995, 970, 885, 840, 770, 745, 720, 705, 615, 580, 530.

Exact mass ESI MS: $C_{11}H_{12}O_2Br_2$ $[M+H]^+$ calculated: 334.9277 found: 334.9266.

Synthesis of 9

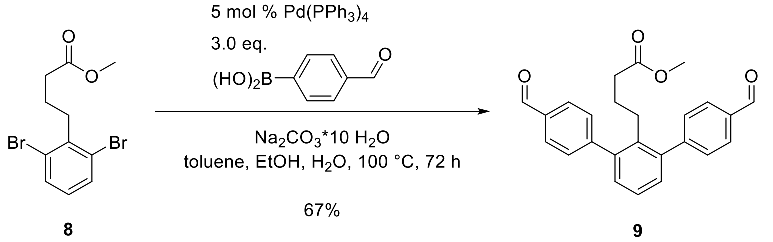

8 9

Scheme S3. Synthesis of **9**.

Toluene (8 mL), ethanol (5 mL) and water (2 mL) were added to a 2-necked Schlenk flask under argon. **8** (100 mg, 0.298 mmol, 1.00 eq), 4-formylphenylboronic acid (98.4 mg, 0.656 mmol, 2.20 eq) and Na_2CO_3*10H_2O (0.545 g, 1.91 mmol, 6.40 eq) were added to the mixture. While stirring, argon was bubbled through the mixture for 30 minutes. $Pd(PPh_3)_4$ (34.4 mg, 0.0291 mmol, 5 mol%) was added and the mixture was stirred under argon for 72 h at 100 °C (oil bath temperature). Afterwards, the mixture was allowed to cool to room temperature. Water (10 mL) and ethyl acetate (10 mL) were added and the phases were separated. The organic phase was washed with water (2 x 10 mL) and dried over MgSO4. Afterwards, removal of solvents under reduced pressure until a volume of approx. 10 mL gave a suspension that was filtered and extracted with toluene. The solvent of the filtrate was removed under reduced pressure to give the crude product that was further purified by column chromatography (DCM:EtOH = 199:1, R_F = 0.05) as eluent. The product was obtained as a colourless oil (73,0 mg, 0.198 mmol, 67%).

^{1}H NMR (300 MHz, $CDCl_3$) δ 10.09 (s, 2H), 7.96 (d, J = 8.3 Hz, 4H), 7.54 (d, J = 8.0 Hz, 4H), 7.34 (dd, J = 8.4, 6.7 Hz, 1H), 7.22 (d, J = 7.0 Hz, 1H), 7.22 (d, J = 8.4 Hz, 1H), 3.41 (s, 3H), 2.66 – 2.52 (m, 2H), 1.82 (t, J = 7.3 Hz, 2H), 1.45 – 1.26 (m, 2H).

^{13}C NMR (101 MHz, CDCl3) δ 192.06, 173.22, 148.51, 141.92, 136.40, 135.34, 130.18, 130.01, 129.83, 126.16, 76.84, 51.52, 33.78, 29.33, 25.73.

IR (ATR-FTIR): 3085, 2950, 2830, 2730, 1730, 1695, 1605, 1565, 1460, 1435, 1410, 1285, 1370, 1305, 1250, 1205, 1165, 1155, 1105, 1015, 1005, 835, 800, 775, 730, 685, 650, 635, 610, 565, 540, 520, 495.

Exact mass ESI MS: $C_{25}H_{22}O_4$ $[M+H]^+$ calculated: 387.1591 found: 387.1588.

Synthesis of 6

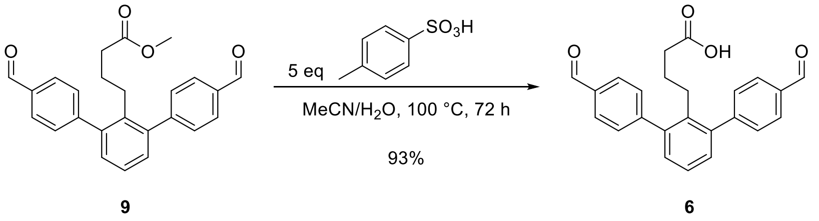

Scheme S4. Synthesis of **6**.

9 (116 mg, 0.300 mmol, 1.00 eq) was dissolved in Acetonitrile (50 ml) and placed in a 2-necked Schlenk flask under argon. Water (50 ml) was added and argon was bubbled through the mixture for 30 minutes. P-Toluenesulfonic acid monohydrate (285 mg, 1.50 mmol, 5.00 eq) was added and the reaction mixture was heated to 100 °C and stirred for 72 hours. After cooling, acetonitrile was evaporated and the resulting suspension was filtered. The precipitate was washed with water (2 x 20 ml) and extracted with acetonitrile. After evaporating the solvent, the residue was collected with dichloromethane and precipitated with pentane. The product was obtained as a white solid (104 mg, 27.9 mmol, 93%).

^{1}H NMR (400 MHz, $CDCl_3$) δ 10.04 (s, 2H), 8.02 – 7.82 (m, 4H), 7.57 – 7.50 (m, 4H), 7.34 (dd, J = 8.3, 6.9 Hz, 1H), 7.22 (d, J = 7.5 Hz, 2H), 2.70 – 2.55 (m, 2H), 1.97 – 1.78 (m, 2H), 1.39 – 1.28 (m, 2H).

^{13}C NMR (101 MHz, $CDCl_3$) δ 192.09, 178.60, 148.40, 141.95, 136.30, 135.31, 130.17, 129.96, 129.81, 126.17, 33.32, 29.14, 25.30.

IR (ATR-FTIR): 3050, 2960, 2820, 2725, 1695, 1604, 1565, 1465, 1440, 1330, 1300, 1380, 1200, 1170, 1155, 1105, 1035, 1015, 1005, 935, 835, 800, 785, 755, 730, 690, 650, 630, 576, 540, 520, 495, 420.

Exact mass ESI MS: $C_{24}H_{20}O_4$ $[M+H]^+$ calculated: 373.1434 found: 373.1436.

Synthesis of 2

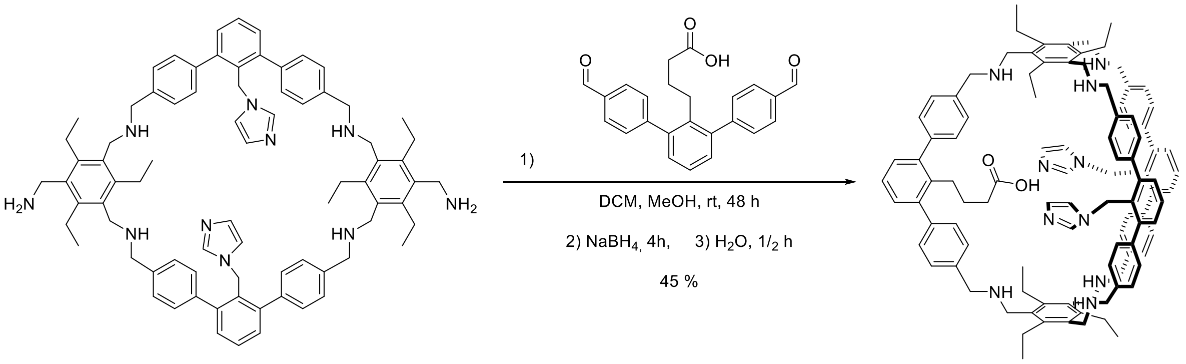

Scheme S5. Synthesis of **2**.

5 (406.9 mg, 0.349 mmol, 1.00 eq) and **6** (130 mg, 0.349 mmol, 1.00 eq) were placed in a Schlenk flask under argon. Dry dichloromethane (40 mL) and dry methanol (6 mL) were added and the reaction mixture was stirred for 48 h at room temperature. $NaBH_4$ (50.0 mg, 1.32 mmol, 3.80 eq) was added and the resulting mixture was stirred for 4 h at room temperature. After addition of water (40 ml) the mixture was stirred for 10 minutes and the organic solvent was removed in vacuo. The precipitate was filtered off, washed with water (2 x 10 ml), acetonitrile (2 x 10 ml) and extracted with dichloromethane (20 ml). The solution was treated with concentrated hydrochloric acid (20 ml) and the mixture was filtered. The residue was washed with dichloromethane (2 x 10 ml), HCl (aq, conc, 5 ml) and afterwards extracted with diluted hydrochloric acid (50 ml). Triethylamine was added until a solid precipitates from

the basic solution that was filtered off, washed with Triethylamine in water (10%, 2 x 10 ml), acetonitrile (2 x 10 ml), diethyl ether (100 ml) and finally extracted with benzene (50 ml). The product was obtained as a white solid (411 mg, 273 μmol, 78%).

^{1}H NMR (500 MHz, CD_2Cl_2) δ 7.43 (t, *J* = 7.6 Hz, 2H), 7.39 (d, *J* = 8.1 Hz, 4H), 7.35 (d, *J* = 8.0 Hz, 8H), 7.24 (d, *J* = 7.7 Hz, 4H), 7.21 (d, *J* = 8.1 Hz, 4H), 7.19 (dd, *J* = 8.1, 7.0 Hz, 1H), 7.08 (d, *J* = 7.6 Hz, 2H), 7.06 (d, *J* = 8.1 Hz, 8H), 6.74 (t, *J* = 1.2 Hz, 2H), 6.45 (t, *J* = 1.2 Hz, 2H), 6.21 (t, *J* = 1.4 Hz, 2H), 4.86 (s, 4H), 3.91 (s, 4H), 3.88 (s, 8H), 3.87 (s, 4H), 3.86 (s, 8H), 2.99 – 2.85 (m, 12H), 2.42 – 2.31 (m, 2H), 1.72 – 1.64 (m, 2H), 1.31 (td, *J* = 7.5, 5.3 Hz, 18H), 1.27 – 1.18 (m, 2H).

^{1}H DOSY NMR (500 MHz, CD_2Cl_2): $D = 5.6*10^{-10}$ m^2 s^{-1}.

^{13}C NMR (126 MHz, $CDCl_3$) δ 174.65, 144.35, 142.95, 142.62, 142.49, 141.42, 140.50, 139.60, 139.56, 138.23, 136.73, 134.47, 134.28, 130.51, 129.87, 129.82, 129.35, 129.05, 128.70, 128.67, 128.34, 127.34, 125.38, 119.18, 55.73, 55.49, 48.91, 48.83, 45.53, 35.27, 29.80, 26.17, 22.93, 17.23.

IR (ATR-FTIR): 3310, 3055, 3025, 2960, 2925, 2870, 1710, 1605, 1580, 1570, 1510, 1455, 1400, 1370, 1320, 1300, 1265, 1225, 1185, 1110, 1075, 965, 945, 910, 840, 825, 800, 775, 760, 730, 700, 685, 660, 635, 615, 605, 600, 595, 555, 535.

Exact mass ESI MS: $C_{102}H_{110}N_{10}O_2$ $[M+H]^+$ calculated: 1507.8886 found: 1507.8832.

Statistical approaches towards 2

Method 1

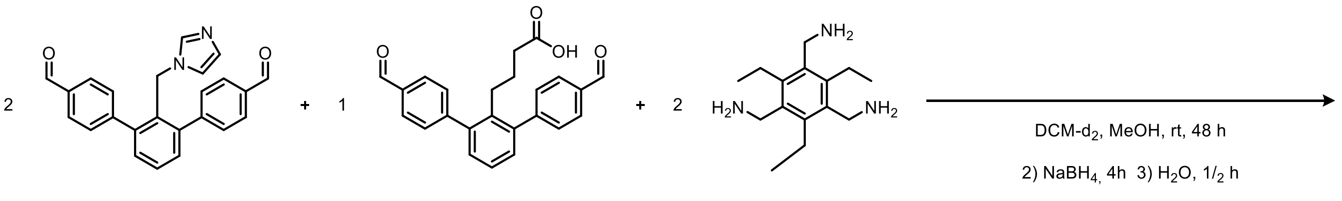

3 6 10

Using stock solutions, **3** (7.3 mg, 20.0 μmol, 2.00 eq in 0.4 ml DCM-d_2), **6** (3.7 mg, 10.0 μmol, 1.00 eq in 0.4 ml DCM-d_2), and **10** (5.0 mg, 20.0 μmol, 2.00 eq in 0.4ml DCM-d_2), were placed in a J-young nmr tube under argon. Dry methanol-d_3 (0.2 mL) was added and the reaction mixture was stirred for 48 h at room temperature. $NaBH_4$ (18.1 mg, 0,478 mmol, 3.30 eq) was added and the resulting mixture was stirred for 4 h at room temperature. After addition of water (20 ml) the mixture was stirred for 10 minutes and the organic solvents were removed in vacuo. The precipitate was filtered off, washed with water (2 x 2 ml), acetonitrile (2 x 2 ml), diethyl ether and extracted with dichloromethane (5 ml). 13.8 mg were obtained.

Methods 2 and 3

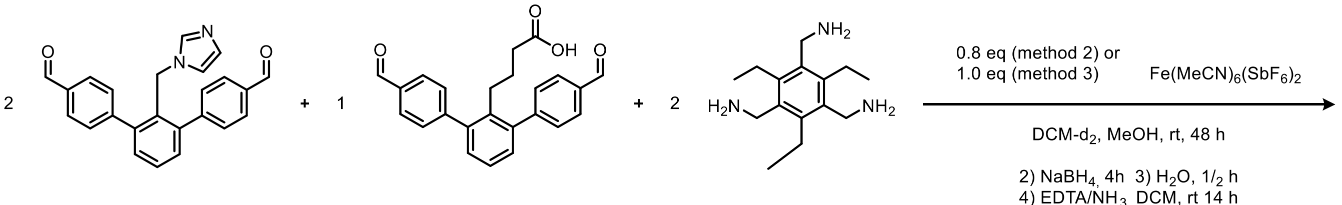

3 6 10

Using stock solutions, $[Fe(MeCN)_6](SbF_6)_2$ (**3** (7.3 mg, 20.0 μmol, 2.00 eq in 0.4 ml DCM-d_2), **6** (3.7 mg, 10.0 μmol, 1.00 eq in 0.4 ml DCM-d_2), and **10** (5.0 mg, 20.0 μmol, 2.00 eq in 0.4ml DCM-d_2), were placed in a J-young nmr tube under argon. Dry methanol-d_3 (0.2 mL) was added and the reaction mixture was stirred for 48 h at room temperature. $NaBH_4$ (1.4 mg, 37 μmol, 3.70 eq) was added and the resulting mixture was stirred for 4 h at room temperature. After addition of water (1 ml) the mixture was stirred for 10 minutes and the organic solvents were removed in vacuo. The precipitate was filtered off, washed with water (2 x 2 ml), acetonitrile (2 x 2 ml) and extracted with dichloromethane (5 ml). A saturated solution of EDTA in 45% aqueous ammonium hydroxide solution (2 ml) was added and the mixture was stirred for 14 hours. The phases were separated, the organic phase was washed with water and dried over magnesium sulfate. 12.4 mg (method 2) and 12.0 mg (method 3) were obtained.

Synthesis of Zn@2

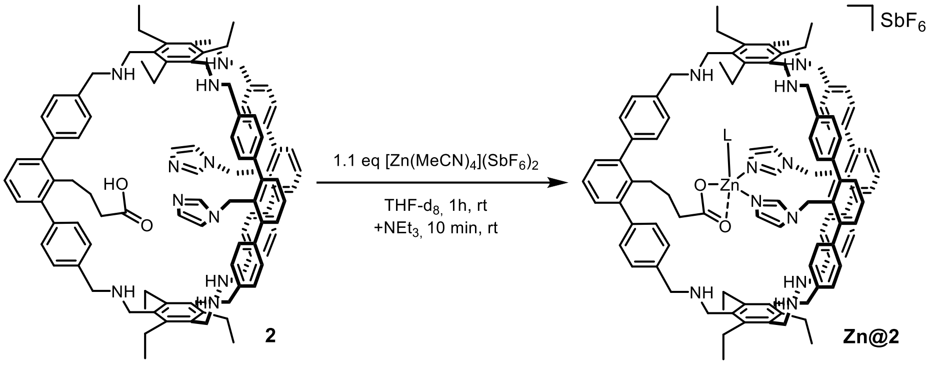

Scheme S6. Synthesis of **Zn@2**.

2 (5.0 mg, 3.3 μmol, 1.0 eq) and $[Zn(MeCN)_4](SbF_6)_2$ (2.5 mg, 3.6 μmol, 1.1 eq) are placed in a j young nmr tube in a glovebox. Dry THF-d_8 (1 ml) is added and stirred for 1 h. Triethylamine (1.7 μL, 13 μmol, 3.8 eq) is added and the resulting suspension is stirred for 10 minutes and filtered afterwards.

1H NMR (600 MHz, THF-d_8) δ 7.51 (t, J = 7.7 Hz, 2H), 7.38 (d, J = 7.8 Hz, 4H), 7.33 – 7.28 (m, 14H), 7.19 (t, J = 8.2 Hz, 1H) 7.17 (d, *J* = 7.5 Hz, 4H), 7.12 – 7.06 (m, 2H), 6.74 (s), 6.68 (s), 4.98 (s), 3.89 (s, 12H), 3.85 (s, 12H), 3.00 – 2.94 (m, 8H), 2.90 (q, J = 7.7 Hz, 4H) 2.60 (t, J = 7.5 Hz, 2H), 1.30 (s, br, 2H), 1.29 (t, J = 7.5 Hz, 12H), 1.25 (t, J = 7.4 Hz, 6H), 1.18 (s, br, 2H).

^{1}H DOSY NMR (500 MHz, THF-d_8): $D = 4.4 \cdot 10^{-10}\ m^2\ s^{-1}$

^{13}C NMR (126 MHz, THF-d_8) δ 145.68, 143.36, 143.20, 143.15, 142.18, 141.41, 140.40, 139.76, 139.00, 138.62, 134.77, 134.71, 130.58, 129.90, 129.81, 129.73, 129.25, 128.91, 128.68, 128.55, 126.38, 125.82, 121.01, 56.03, 55.54, 49.38, 49.26, 47.54, 46.44, 36.06, 30.20, 27.39, 23.03, 22.99, 17.18, 17.12, 11.23.

Exact mass ESI MS: $C_{102}H_{109}N_{10}O_2Zn$ $[M]^+$ calculated: 1561.8083 found: 1561.8063.

IR (ATR-FTIR): 3140, 3050, 3020, 2965, 2930, 2870, 1585, 1530, 1510, 1455, 1435, 1405, 1375, 1325, 1300, 1240, 1180, 1045, 1020, 955, 945, 850, 805, 780, 750, 735, 705, 660, 645, 615, 545.

Synthesis of Fe@2

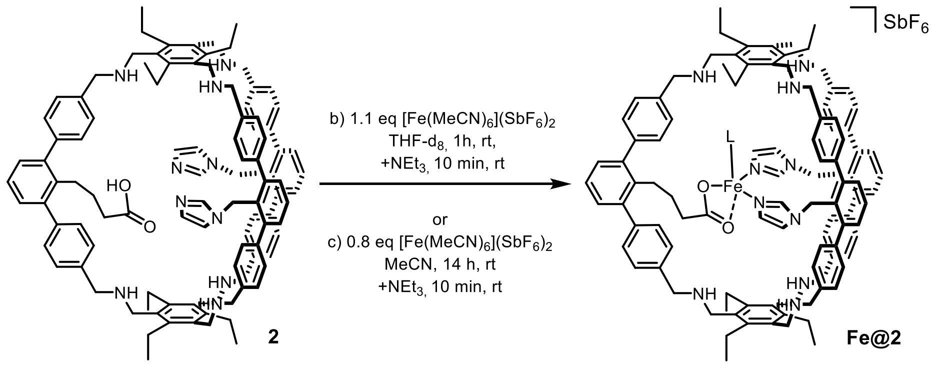

Scheme S7. Synthesis of **Fe@2** (method b and c).

b) **2** (5.0 mg, 3.3 μmol, 1.0 eq) and $[Fe(MeCN)_6](SbF_6)_2$ (2.8 mg, 3.6 μmol, 1.1 eq) are placed in a j young nmr tube in a glovebox. Dry THF-d_8 (1 ml) is added and stirred for 1 h. Triethylamine (1.7 μL, 13 μmol, 3.8 eq) is added and the resulting suspension is stirred for 10 minutes and filtered afterwards.

1H NMR (300 MHz, THF-d8): δ. 71.68, 43.85, 7.83, 7.34, 7.29 – 7.14 (m), 6.99, 6.90, 6.45, 6.03, 4.50 – 3.90 (m), 3.64, 3.45, 1.66, 1.60.

1H NMR (500 MHz, THF-d8) δ 7.84 (s), 7.36 (s), 7.27 (s), 7.19 (s), 7.00 (m), 6.90 (s), 6.45 (s), 6.02 (s), 4.66 – 3.89 (m), 3.43 (s), 1.66 (s), 1.59 (s).

c) **2** (17.0 mg, 11.3 μmol, 1.20 eq) and $[Fe(MeCN)_6](SbF_6)_2$ (7.0 mg, 9.0 μmol, 1.0 eq) are placed in a vial in a glovebox. Acetonitrile (dried and degassed, 15 ml) is added and the suspension is allowed to stir for 20 h. The volume of the reaction solution is reduced to 5 ml, Triethylamine (4.5 μL, 34 mmol, 3.8 eq) is added and the resulting suspension is stirred for 10 minutes and filtered afterwards. The solvent is removed under reduced pressure and an off-white solid (16.5 mg) is obtained.

^{1}H NMR (300 MHz, THF-d_8): δ. 72.19, 44.13, 7.84, 7.35, 7.29- 7.19 (m), 7.00, 6.90, 6.44, 6.02, 4.51- 3.94 (m), 3.65, 3.43, 1.66, 1.59.

^{1}H DOSY NMR (600 MHz, THF-d_8): $D = 4.4 *10^{-10}$ m^2 s^{-1}

^{13}C NMR (126 MHz, THF-d_8) δ 148.26, 145.44, 144.73, 143.67, 142.31, 141.61, 141.40, 137.63, 136.05, 132.22, 130.94, 130.02, 129.25, 129.14, 128.64, 127.75, 127.26, 56.93, 55.93, 55.03, 50.30, 50.10, 48.01, 24.15, 17.94, 17.70, 9.57.

Exact mass ESI MS: $C_{102}H_{109}N_{10}O_2Fe$ $[M]^+$ calculated: 1569.8021 found: 1569.8015.

IR (ATR-FTIR): 3335, 3130, 3060, 3020, 2975, 2935, 2875, 1580, 1570, 1515, 1455, 1435, 1400, 1375, 1315, 1265, 1230, 1185, 1110, 1085, 1045, 1020, 945, 850, 835, 800, 780, 735, 705, 655, 640, 615, 600, 560, 535.

NMR-monitored Reaction of Fe@2 with dioxygen

Fe@2 (6.5 mg) was dissolved in a j young nmr tube in THF-d_8 (0.5 ml) in an argon atmosphere. The atmosphere was exchanged with dioxygen using freeze-pump-thaw cycles. The mixture was allowed to warm up to room temperature and analyzed by 1H NMR spectroscopy.

NMR-monitored Reaction of Fe@2 and α-ketoglutarate with dioxygen

Fe@2 (6.5 mg) was dissolved in a j young nmr tube in THF-d_8 (0.4 ml) in an argon atmosphere in the presence of triethylamine (2 μl). α-ketoglutarate (1.2 mg in 0.1 ml) was added and the reaction product was analyzed by 1H NMR spectroscopy. The atmosphere was exchanged with dioxygen using freeze-pump-thaw cycles. The mixture was allowed to warm up to room temperature and analyzed by 1H NMR spectroscopy.

UV/Vis-monitored Reaction of Fe@2 and α-ketoglutarate with dioxygen

Fe@2 (5.0 mg) was dissolved in a UV VIS cuvette with septum cap in THF (2 ml) in an argon atmosphere in the presence of triethylamine (3.1 μl). α-ketoglutarate (0.9 mg in 0.05 ml) was added and the reaction product was analyzed by UV/Vis spectroscopy. The solution was cooled to -80 °C and the atmosphere was exchanged with dioxygen by bubbling through the cooled solution for 5 minutes. The mixture was allowed to warm up to -20 °C the reaction was monitored by UV/Vis spectroscopy.

Spectra

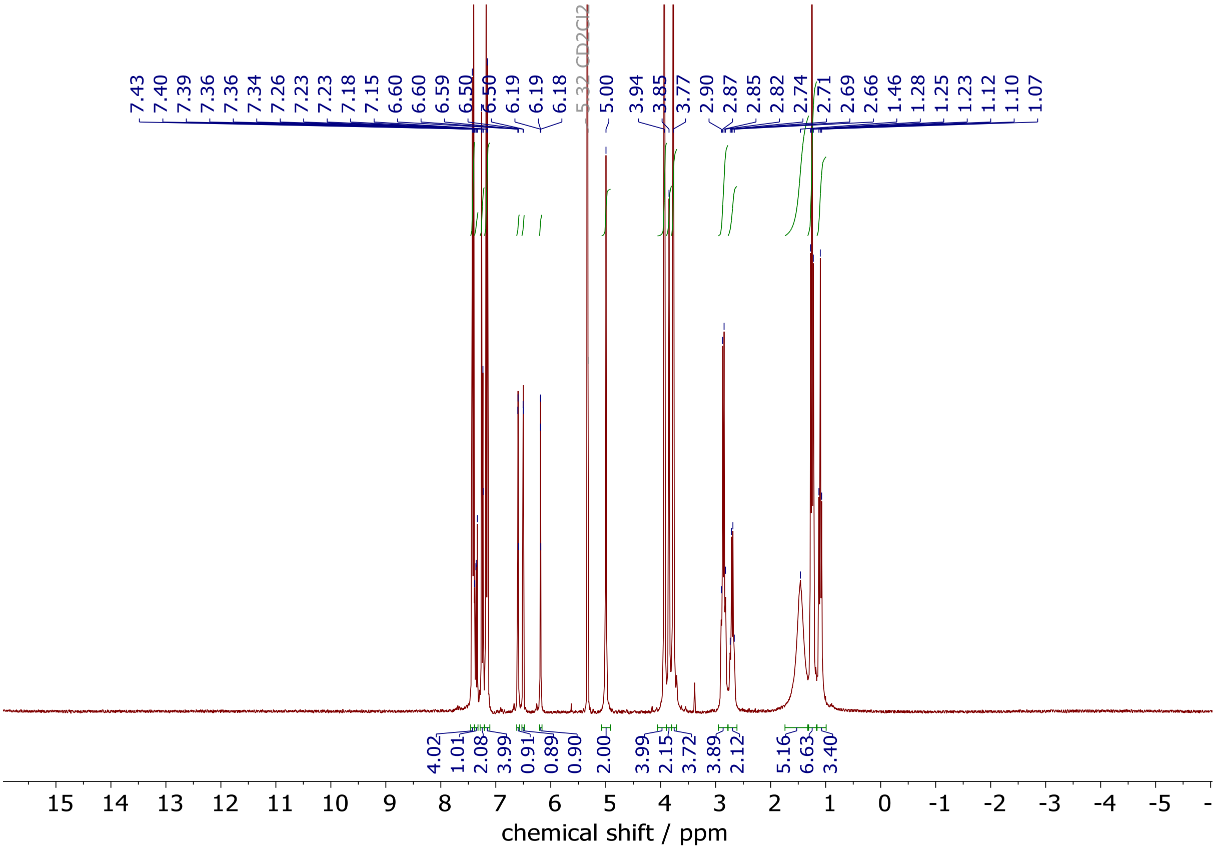

Figure S1. ^{1}H NMR spectrum of **5** in CD_2Cl_2 at rt.

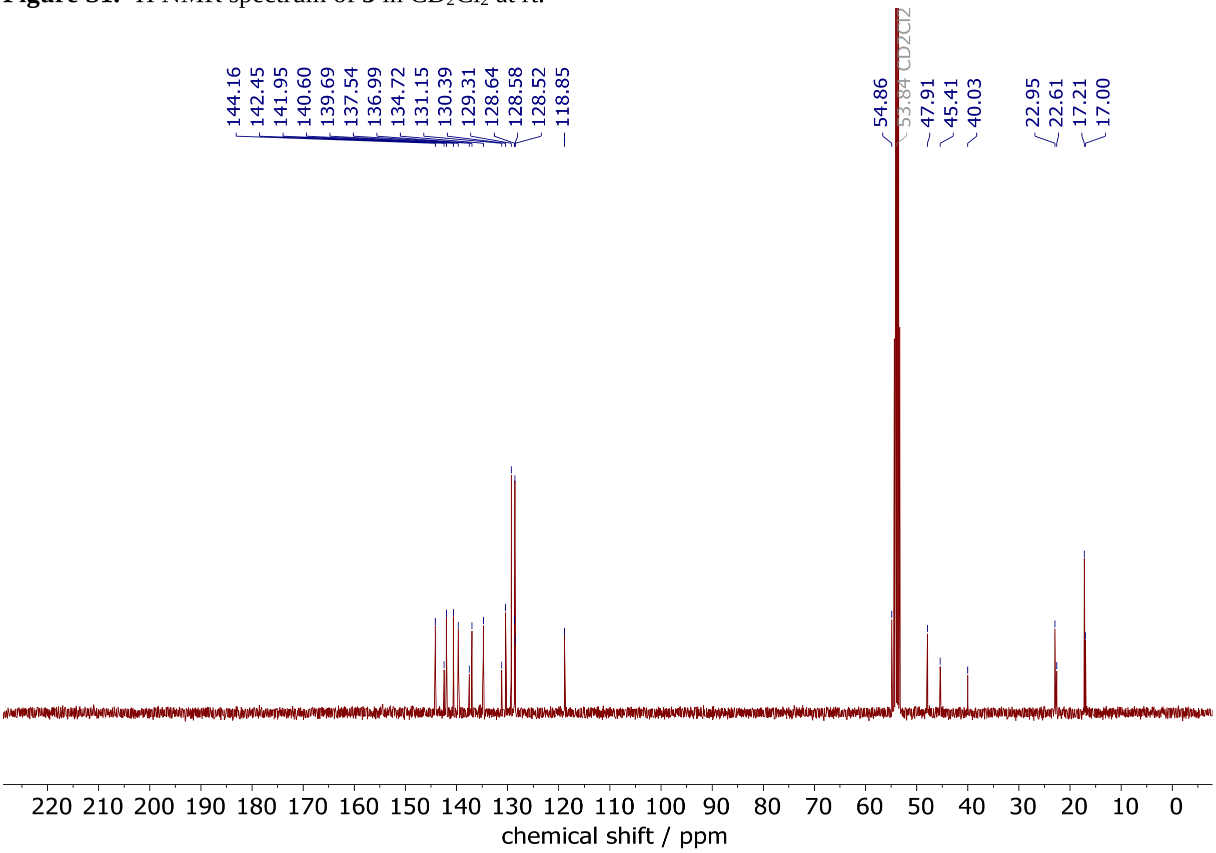

Figure S2. ^{13}C NMR Spectrum of **8** in CD_2Cl_2 at rt.

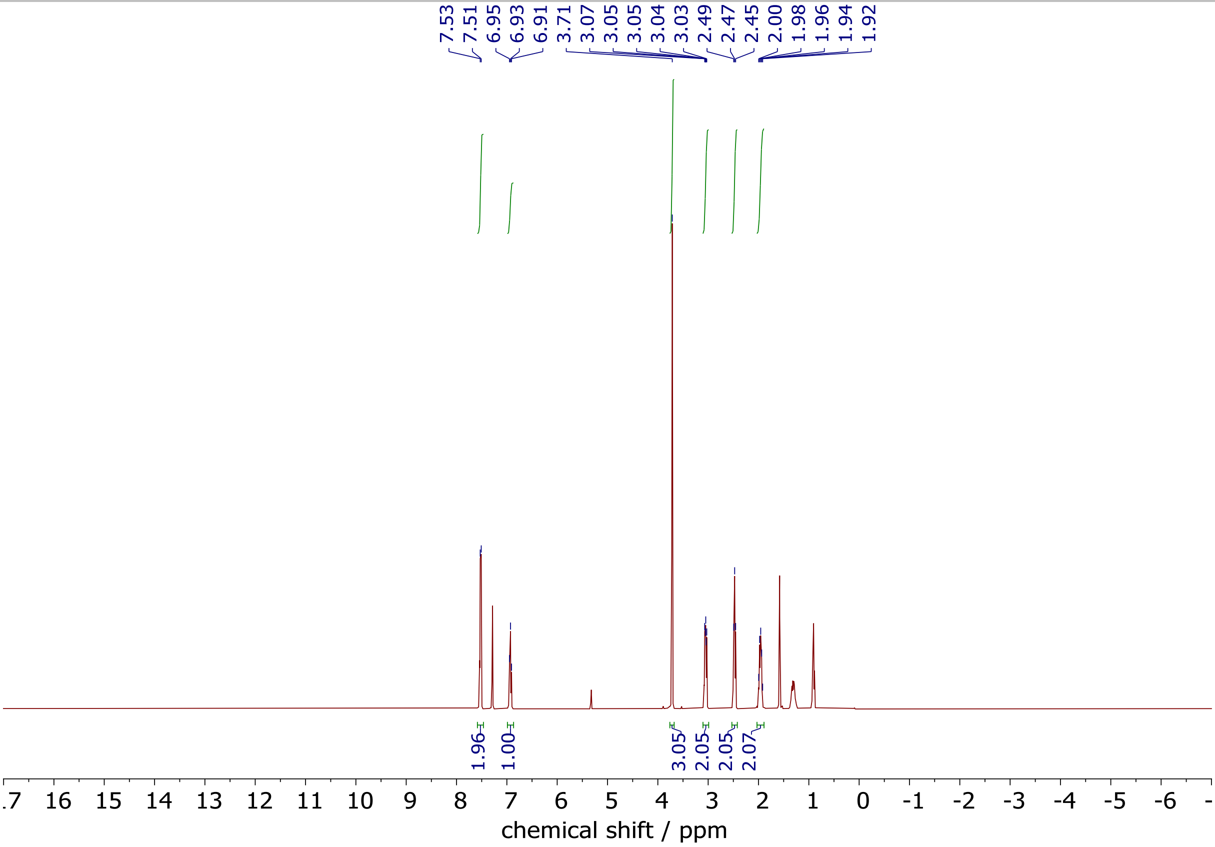

Figure S3. ^{1}H NMR Spectrum of **8** in $CDCl_3$ at rt.

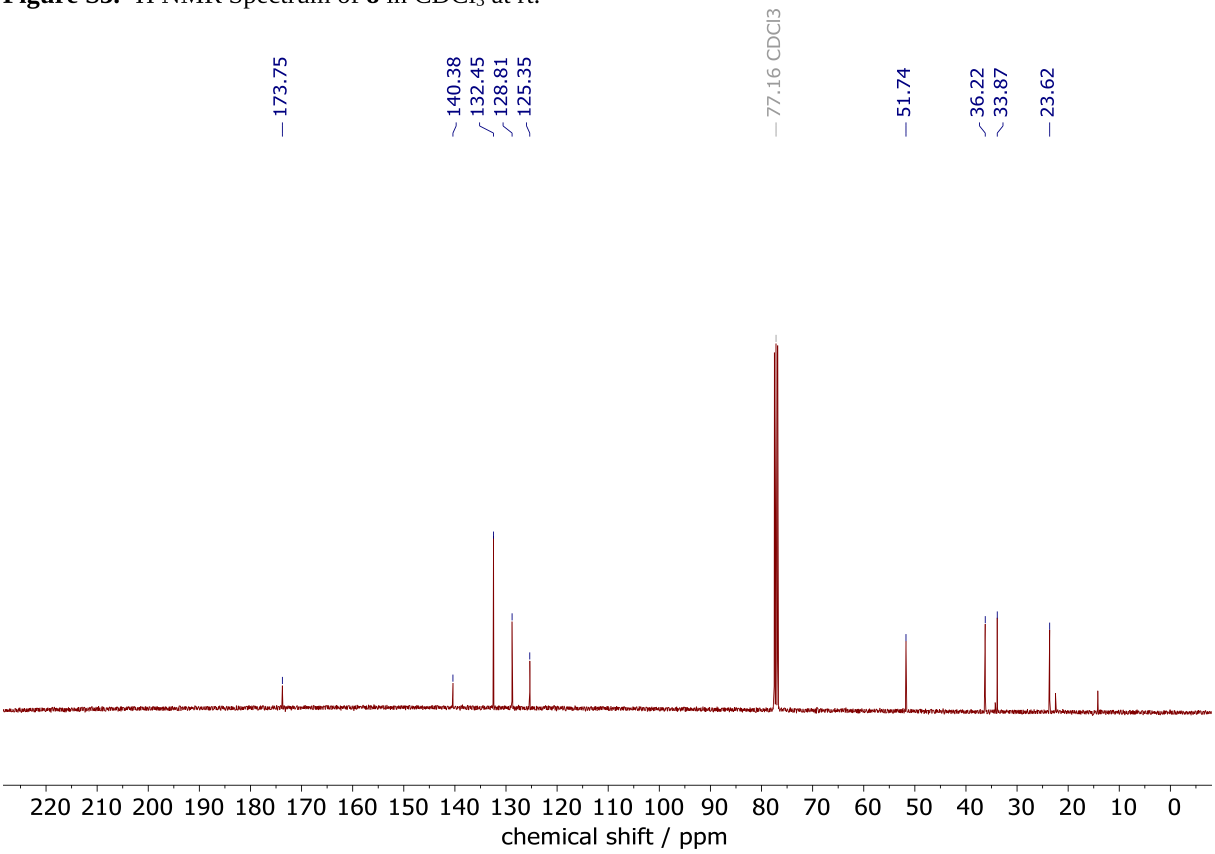

Figure S4. ^{13}C NMR Spectrum of **8** in $CDCl_3$ at rt.

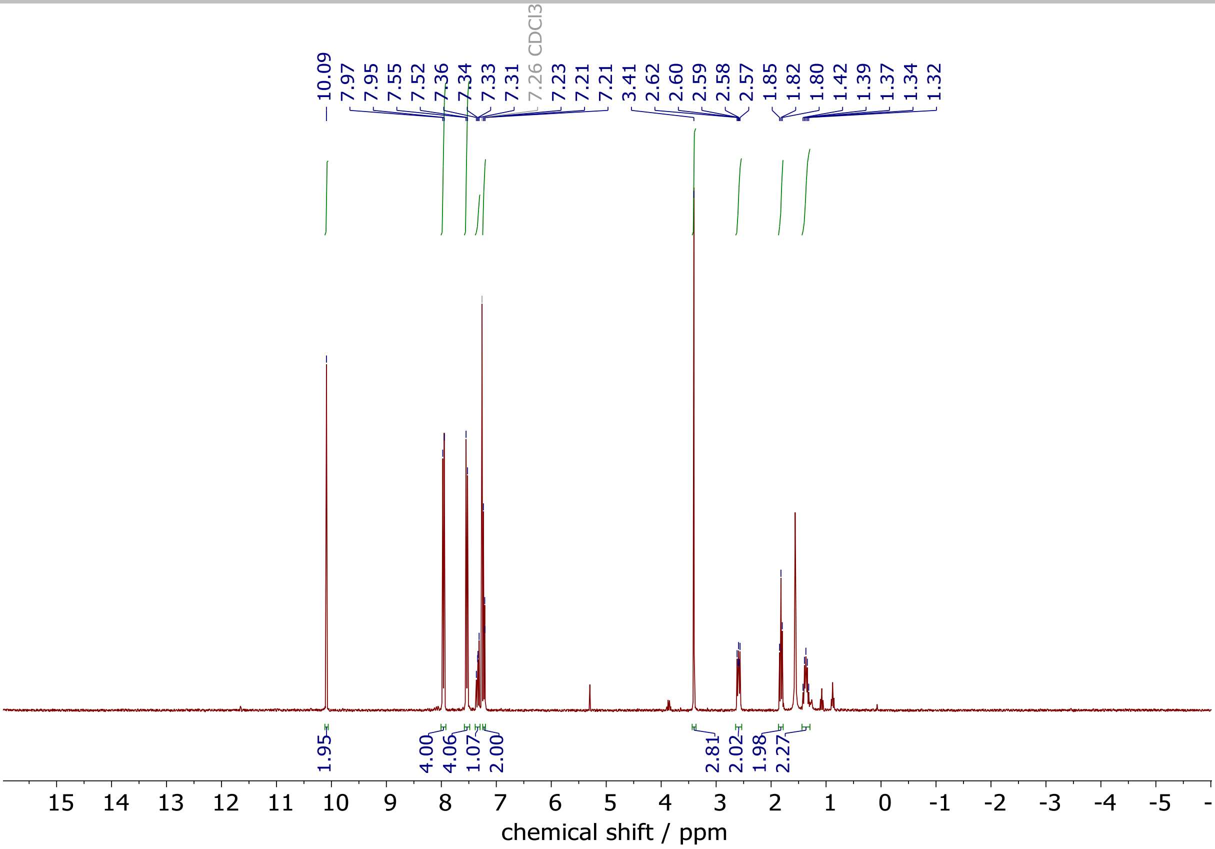

Figure S5. ^{1}H NMR Spectrum of **9** in $CDCl_3$ at rt.

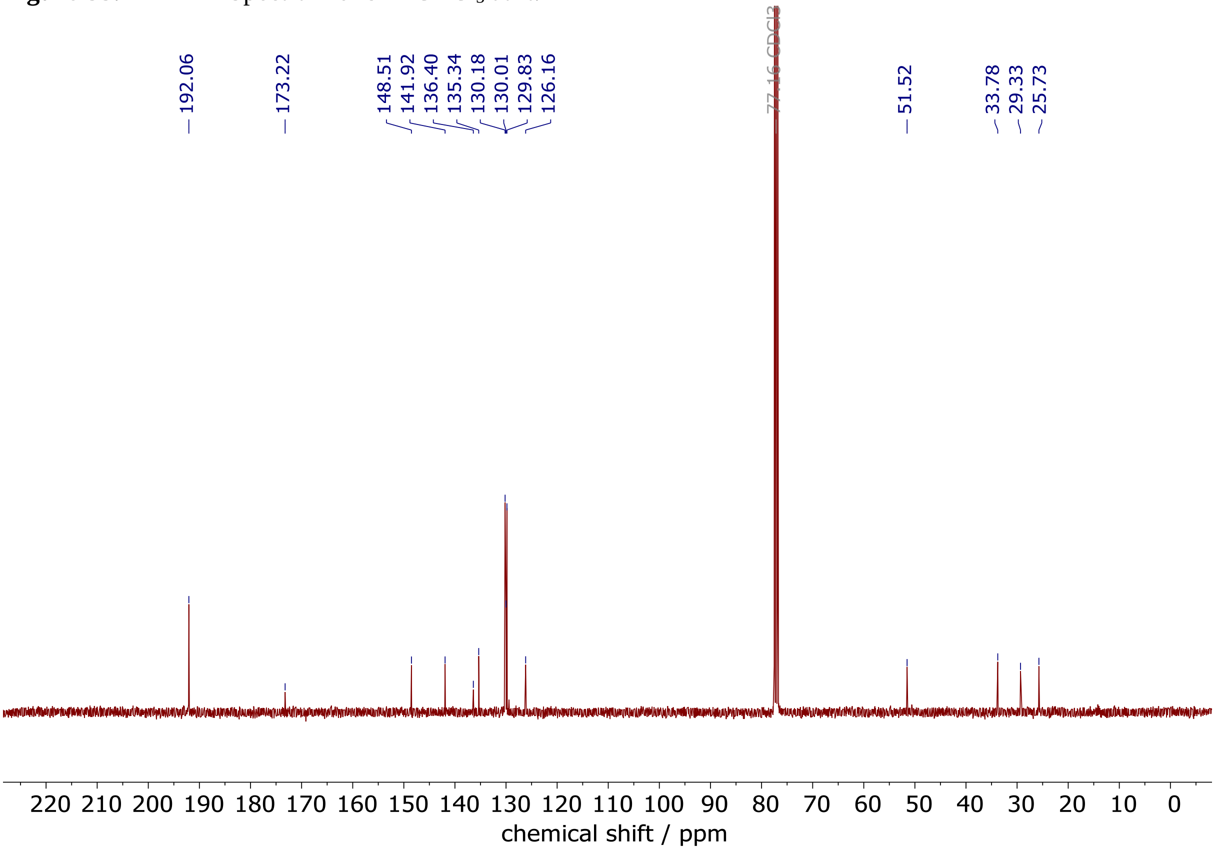

Figure S6. 13C NMR Spectrum of **9** in CDCl3 at rt.

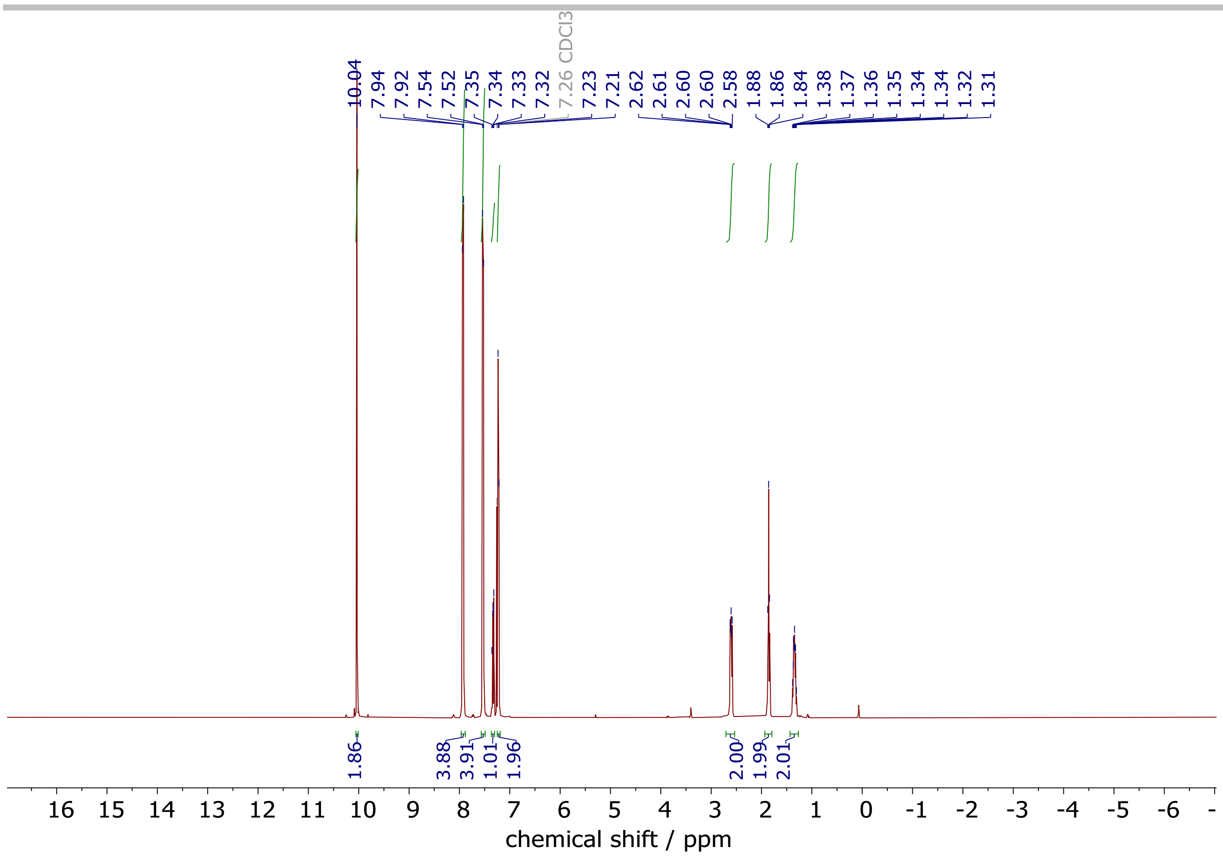

Figure S7: ^{1}H NMR Spectrum of 6 in $CDCl_3$ at rt.

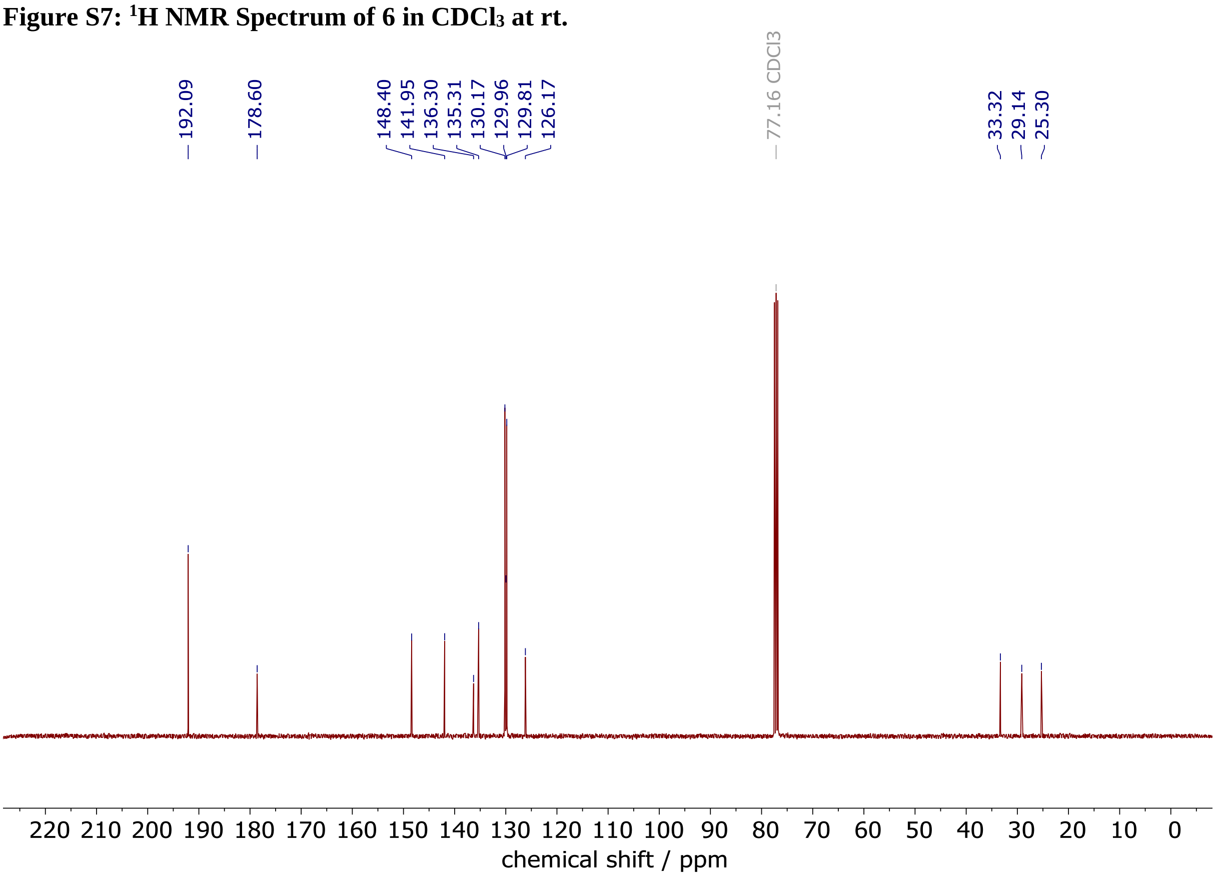

Figure S8. 13C NMR Spectrum of **6** in CDCl3 at rt.

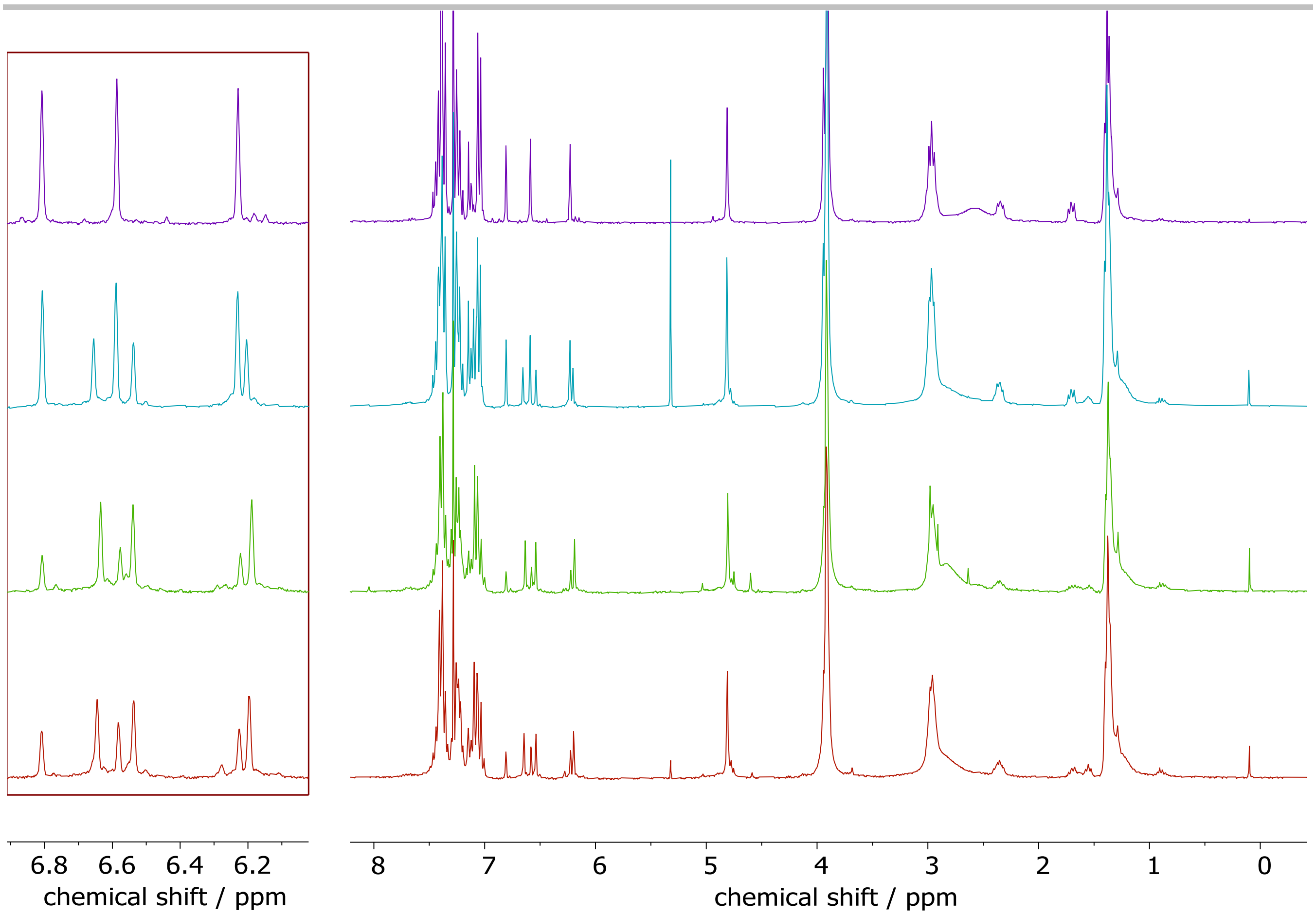

Figure S9. ^{1}H NMR spectra of **2**, and the product mixtures obtained by statistical cage formation methods 1, 2 and 3 (from top to bottom). Cutout shows the region, in which the imidazole CH resonances are expected.

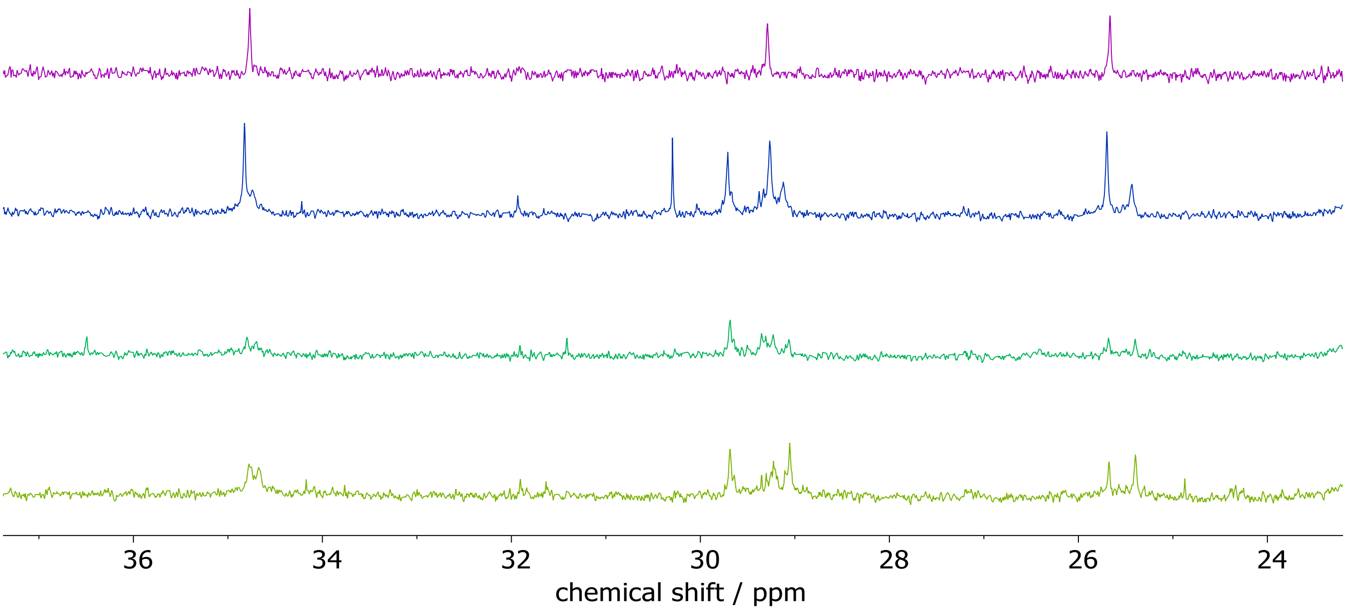

Figure S10. Cutout of the ^{13}C NMR spectra of **2**, and the product mixtures obtained by statistical cage formation methods 1, 2 and 3 (from top to bottom), showing the region, in which all propylene linker CH_2 resonances are expected.

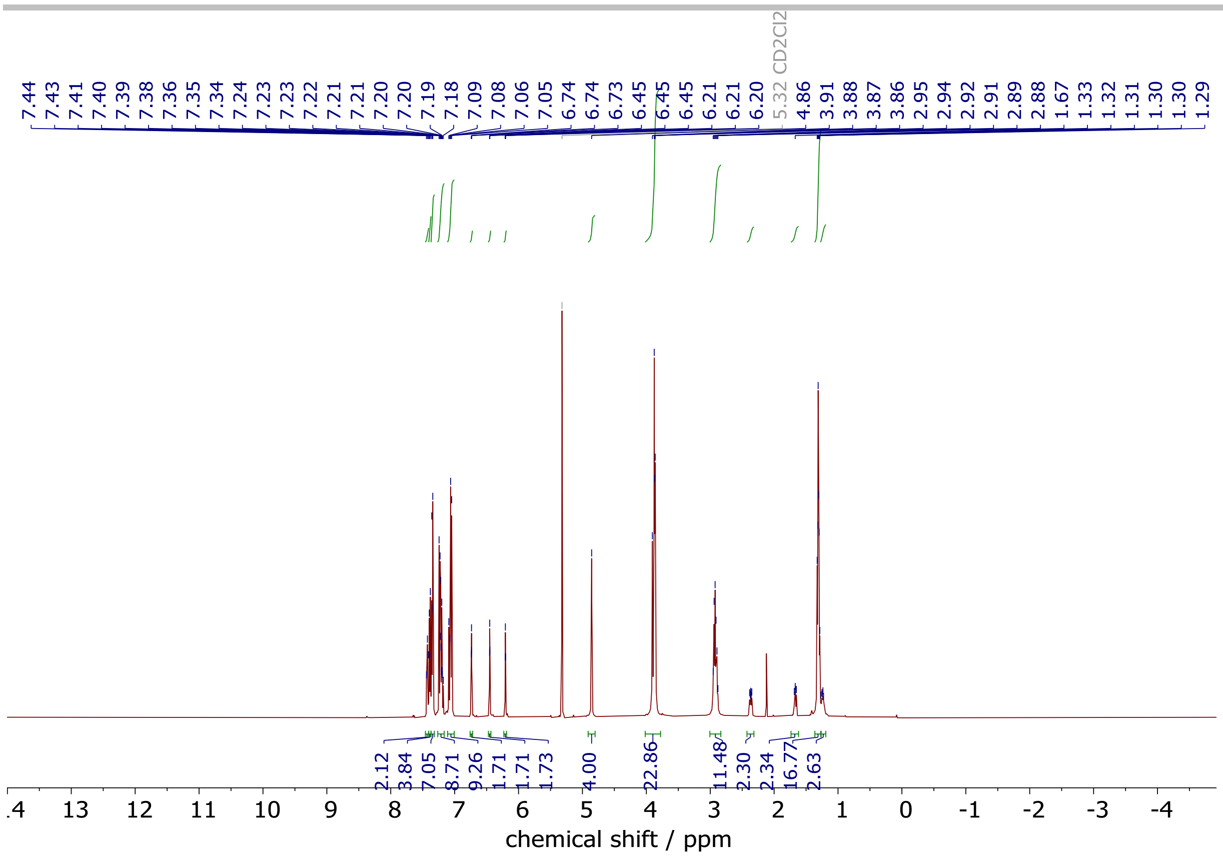

Figure S11. **^{1}H NMR Spectrum of 2 in CD_2Cl_2 at rt.**

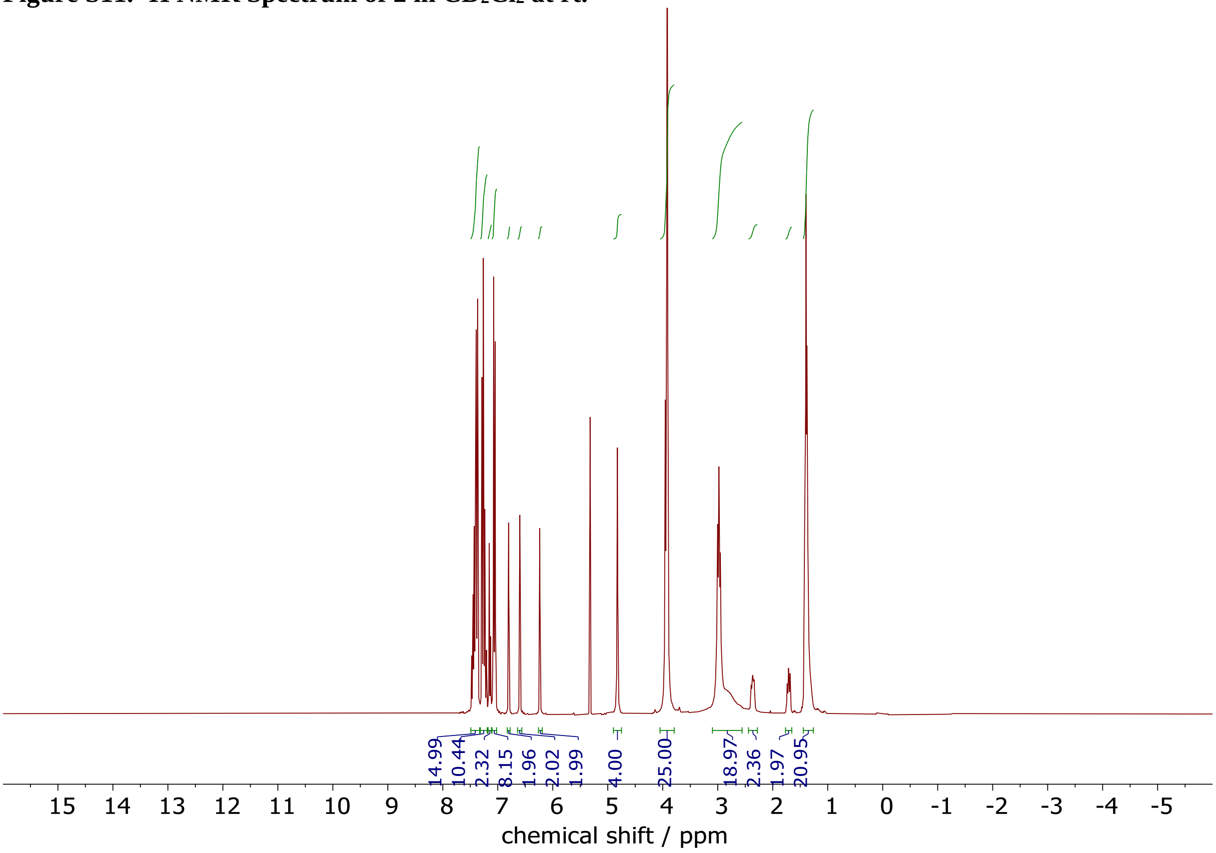

Figure S12. ^{1}H NMR Spectrum of **2** in CD_2Cl_2 at rt.

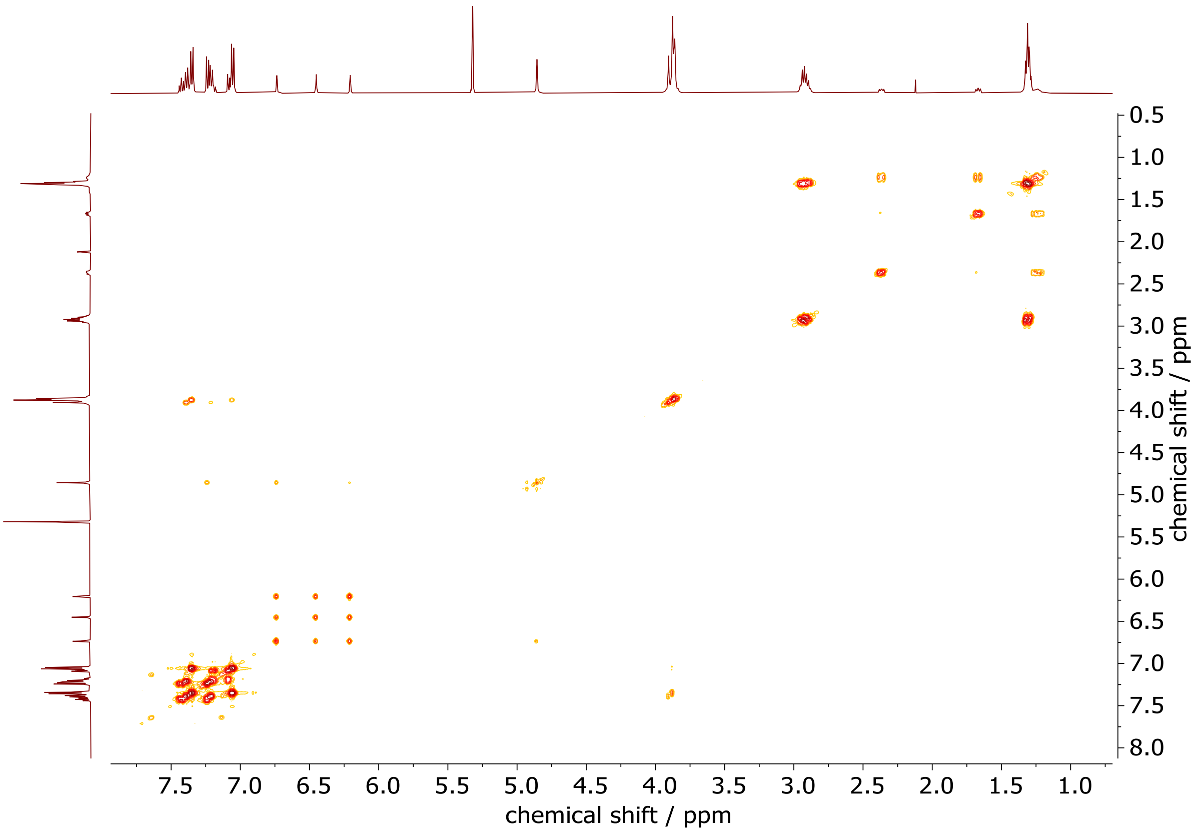

Figure S13. ^{1}H DOSY NMR Spectrum of **2** in CD_2Cl_2 at rt.

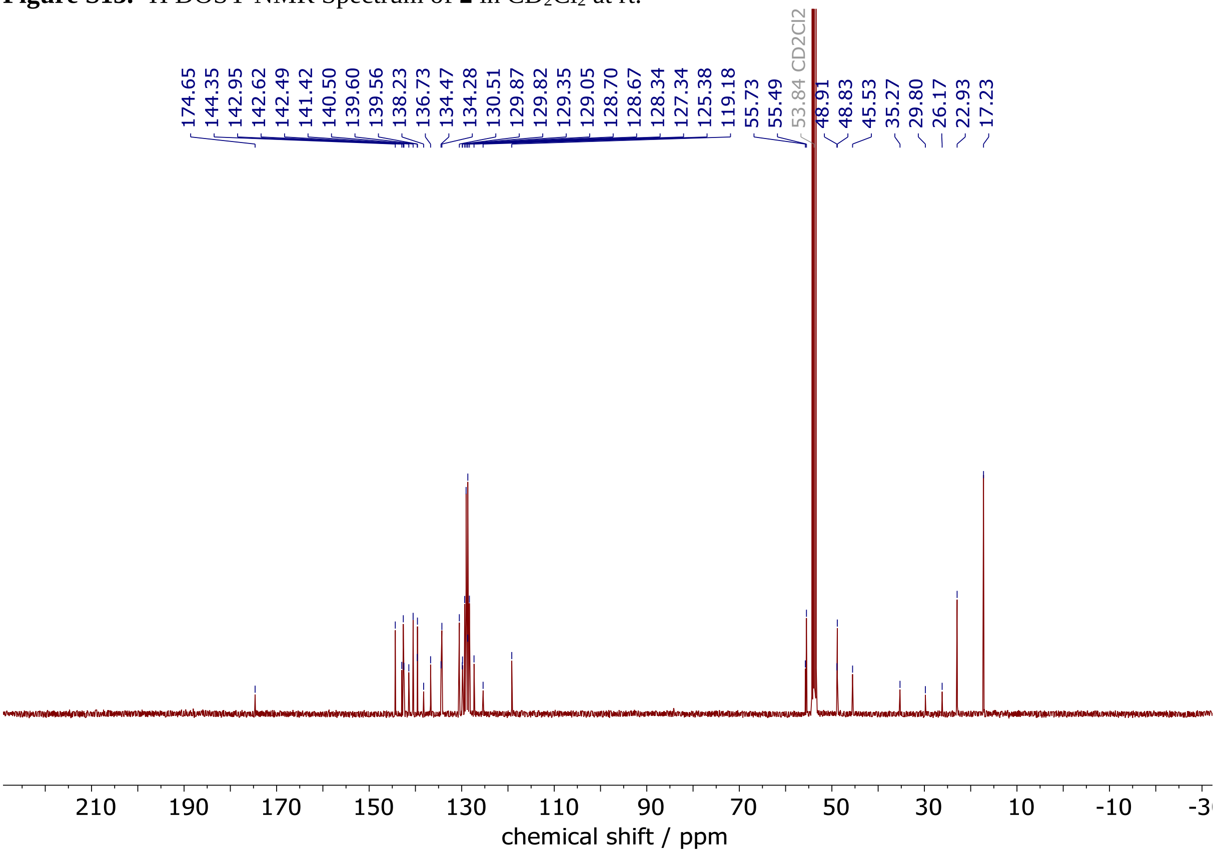

Figure S14. ^{13}C NMR Spectrum of **2** in CD_2Cl_2 at rt.

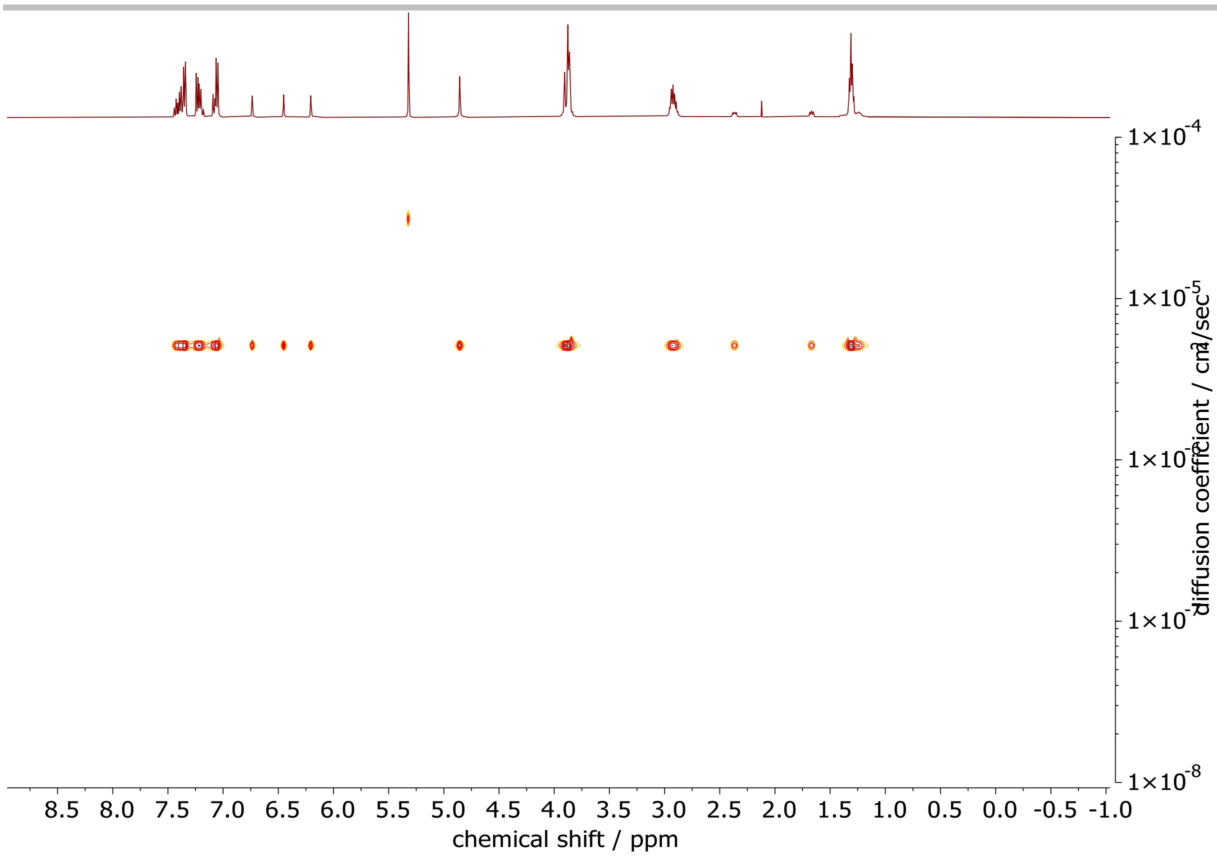

Figure S15. ^{1}H DOSY NMR Spectrum of **2** in CD_2Cl_2 at rt.

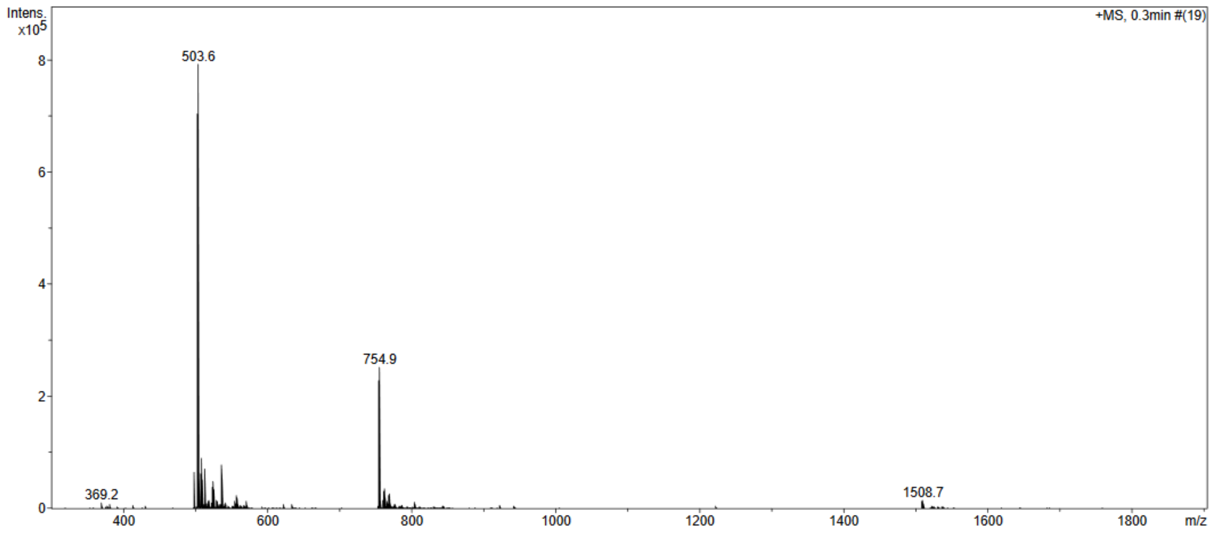

Figure S16. ESI MS of **2**.

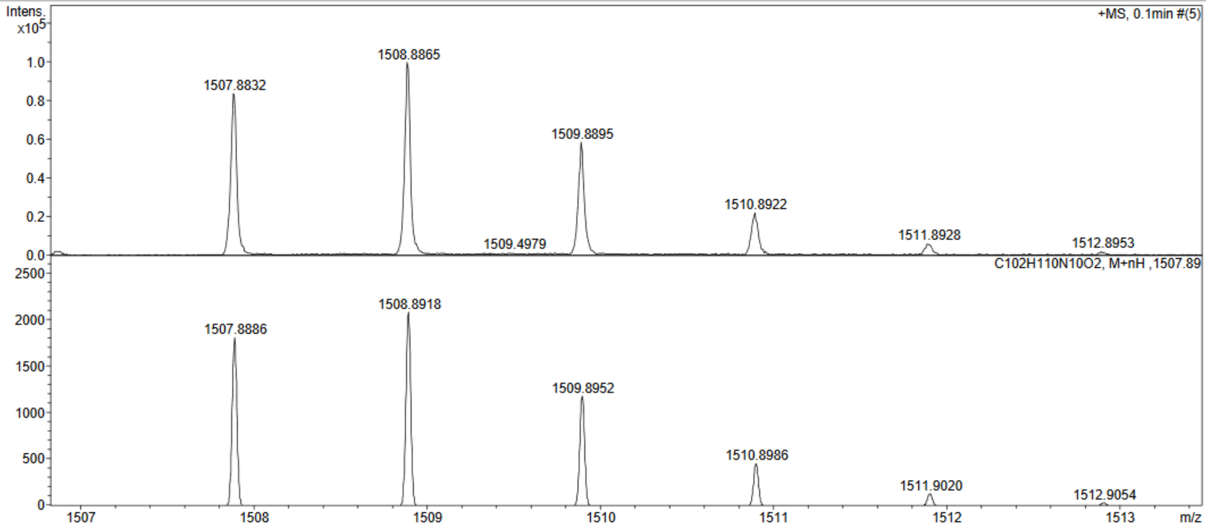

Figure S17. Measured and calculated ESI-MS of **2** for [M+H]$^{+}$.

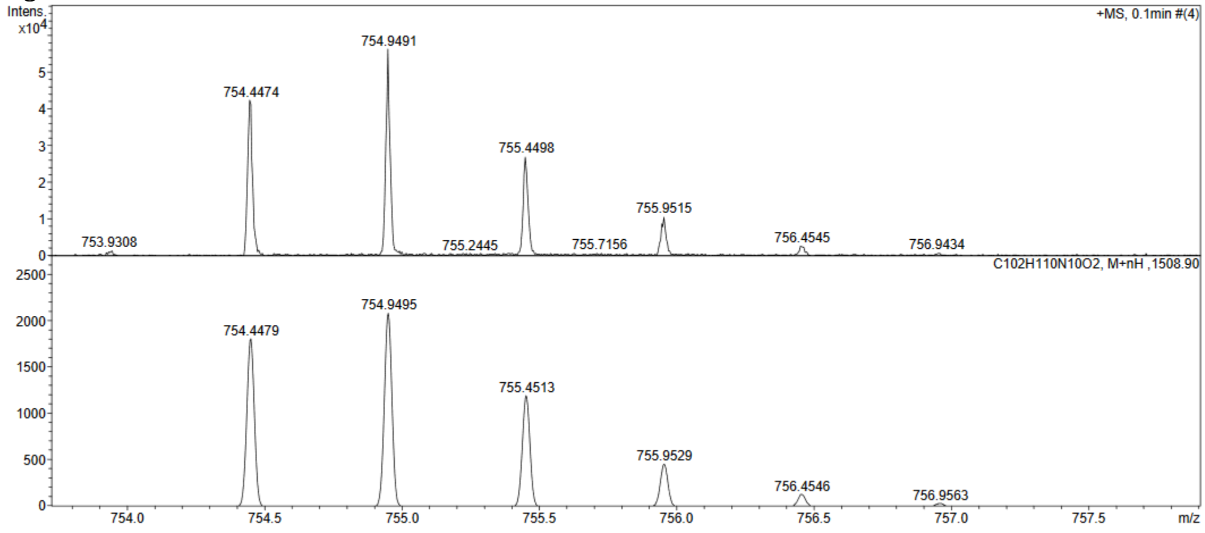

Figure S18. Measured and calculated ESI-MS of **2** for [M+H]$^{2+}$.

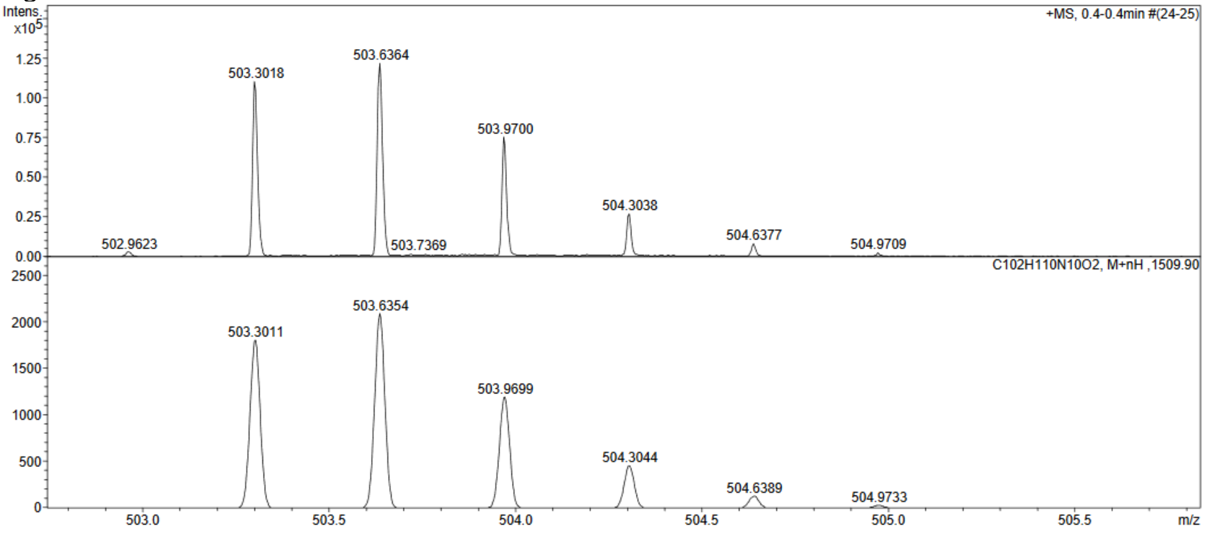

Figure S19. Measured and calculated ESI-MS of **2** for [M+H]$^{3+}$.

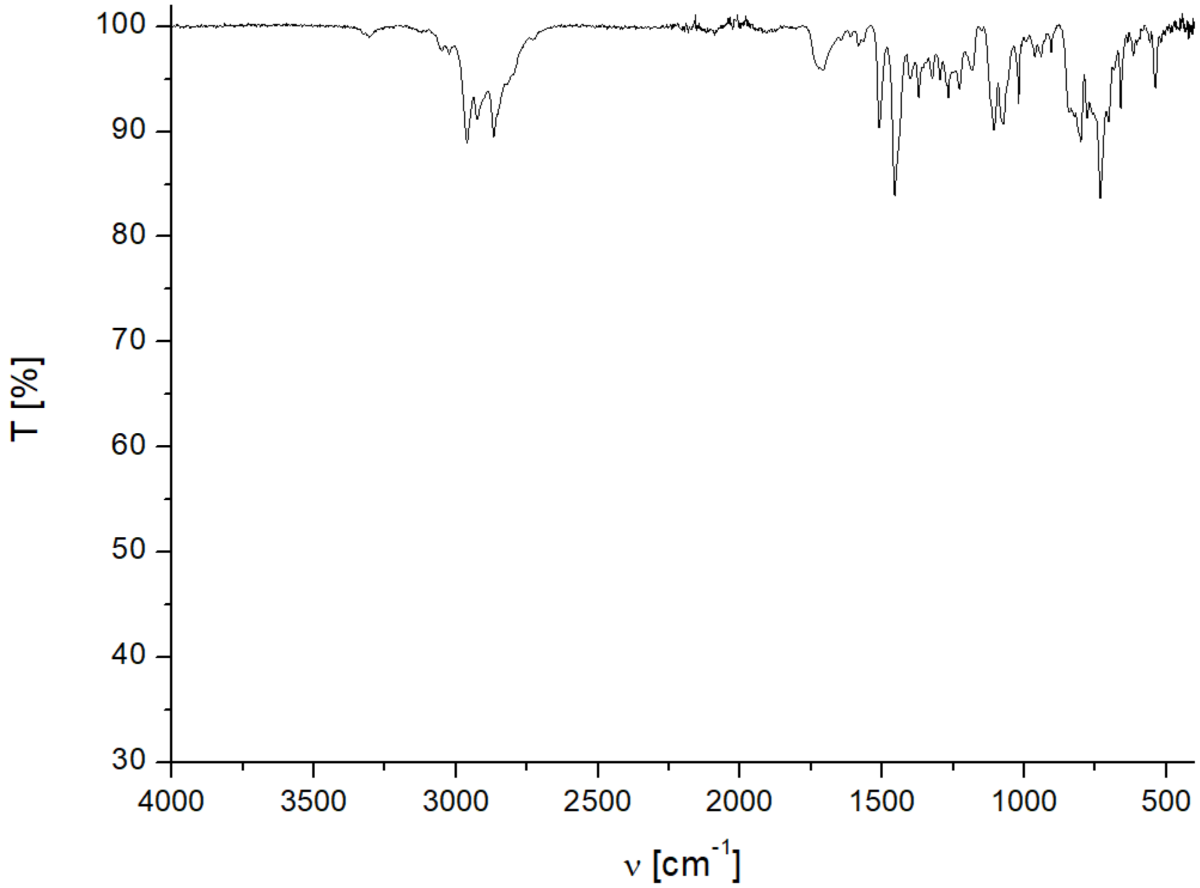

Figure S20. ATR-IR spectrum of **2**.

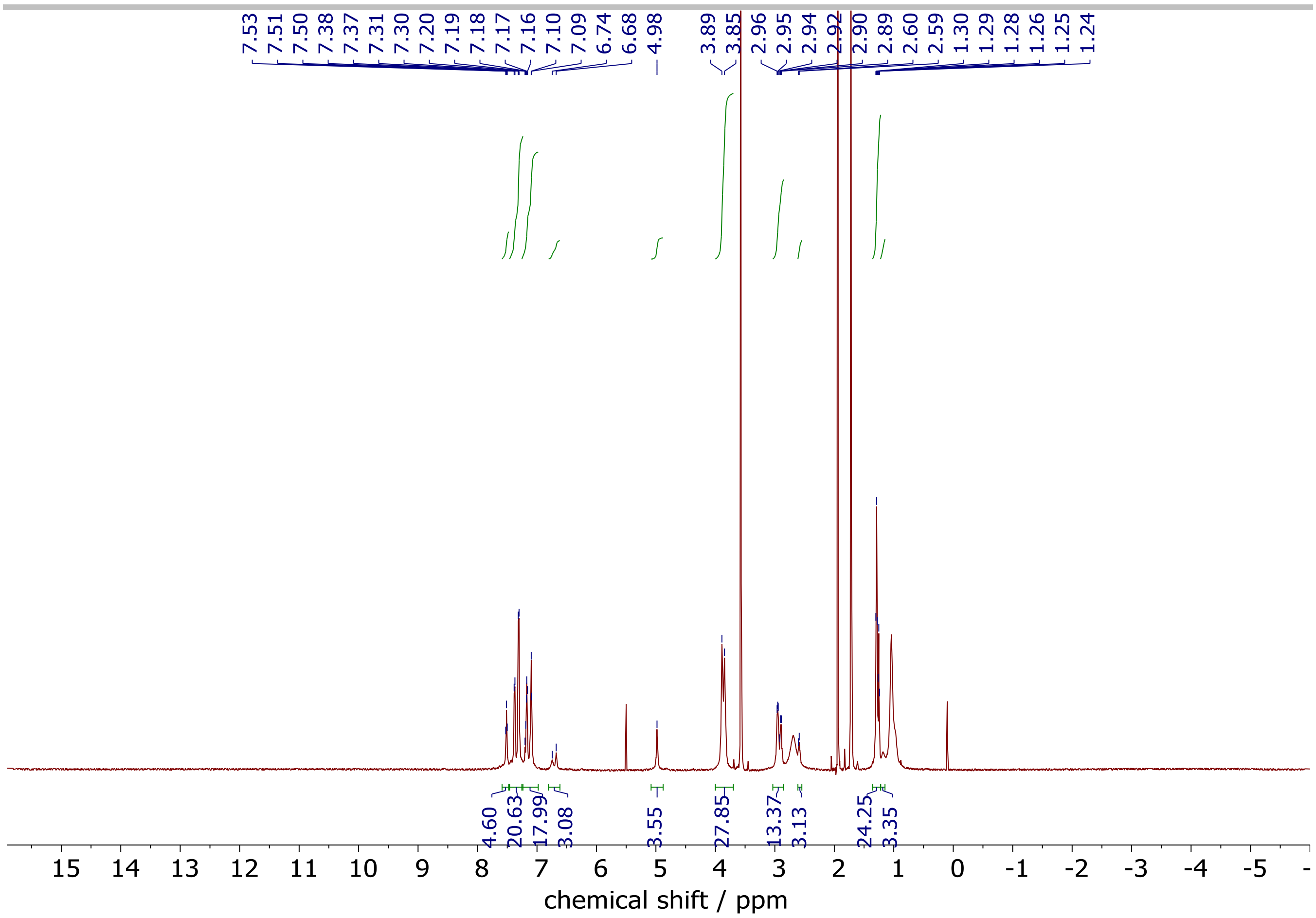

Figure S21. ^{1}H NMR Spectrum of **Zn@2** in THF-d_8 at rt.

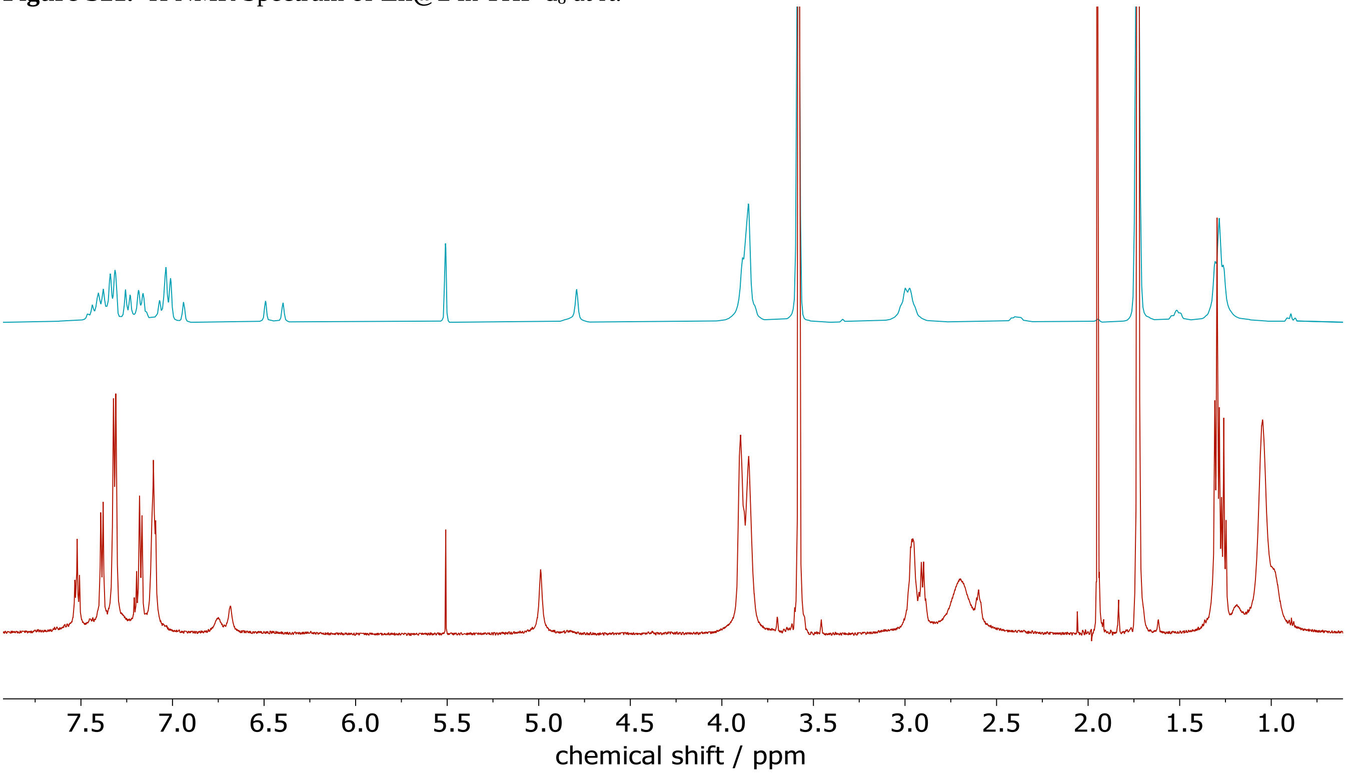

Figure S22. Comparison of the ^{1}H NMR Spectra of **2** (top) and **Zn@2** (bottom) in THF-d_8 at rt.

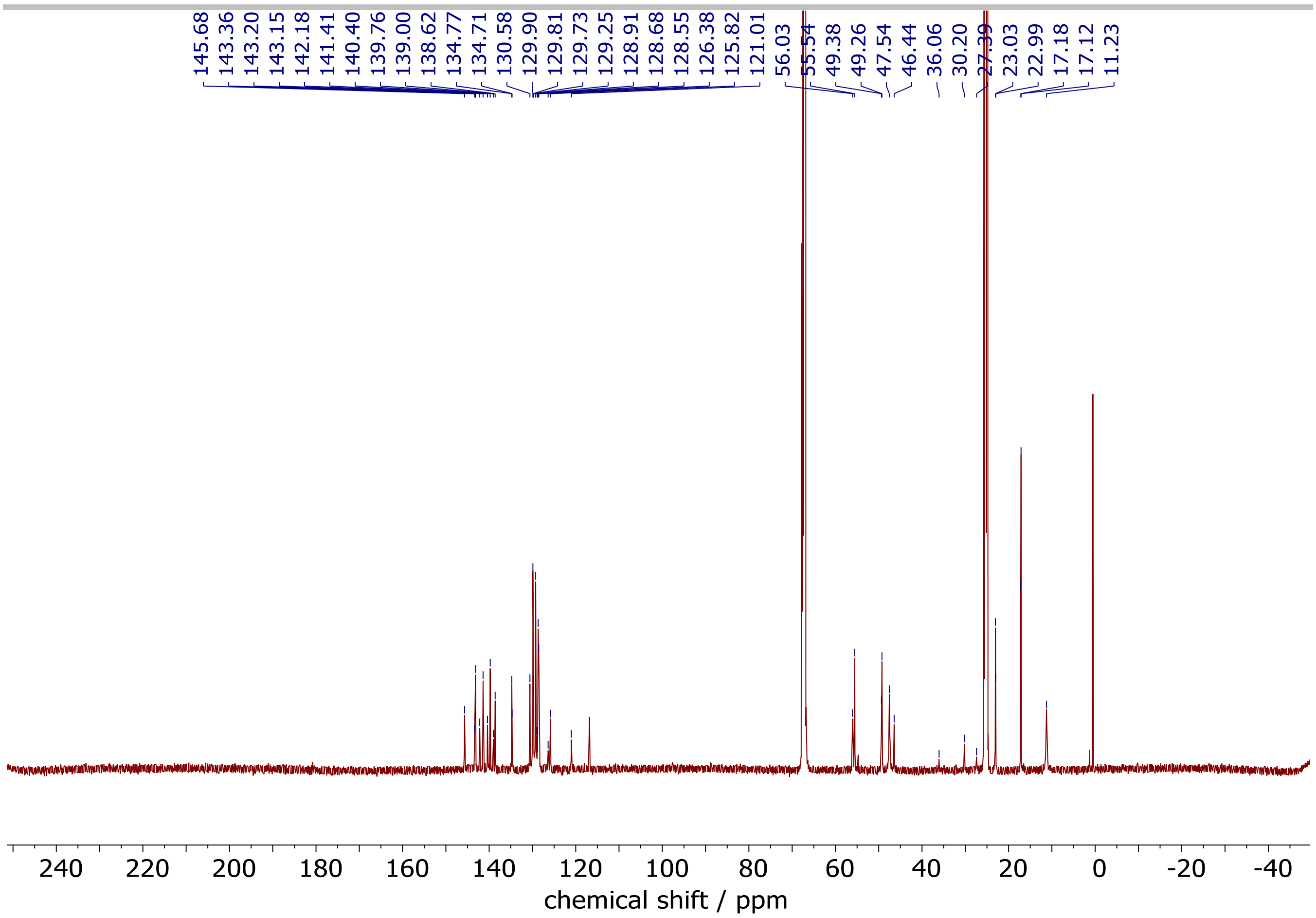

Figure S23. ^{13}C NMR Spectrum of **Zn@2** in THF-d_8 at rt.

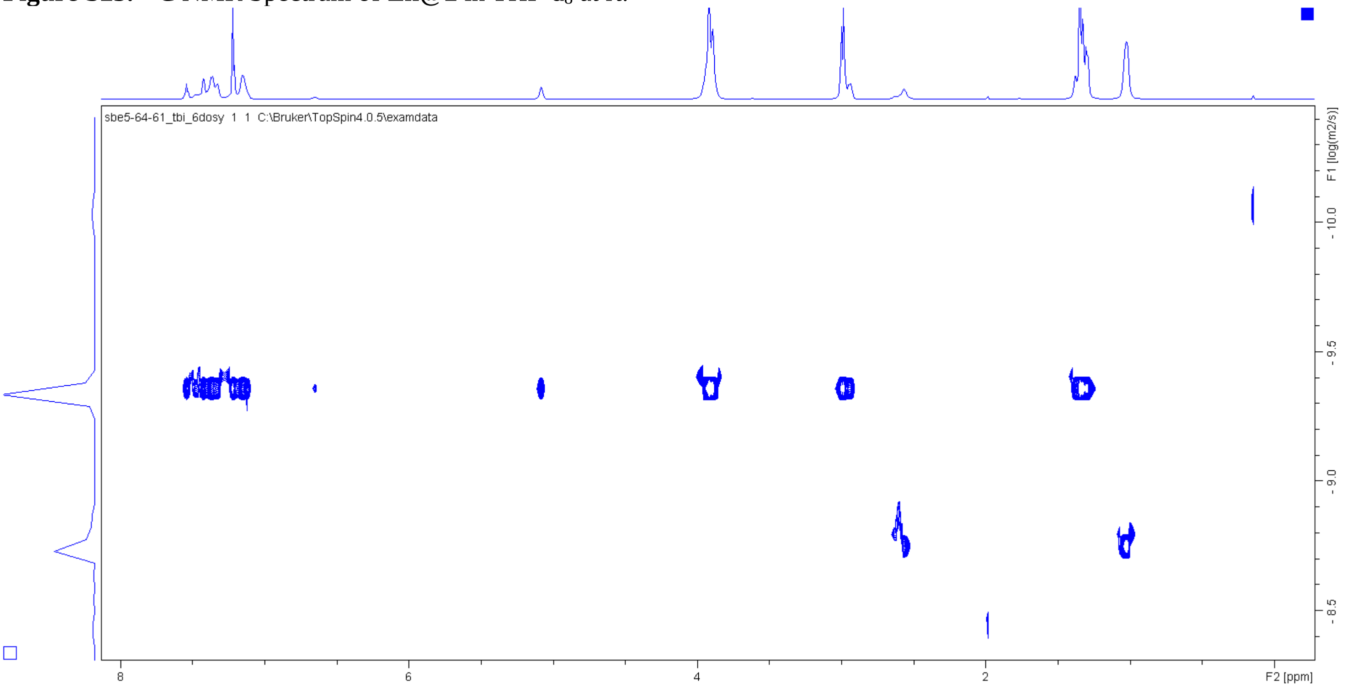

Figure S24. ^{1}H DOSY NMR Spectrum of **Zn@2** in THF-d_8 at rt.

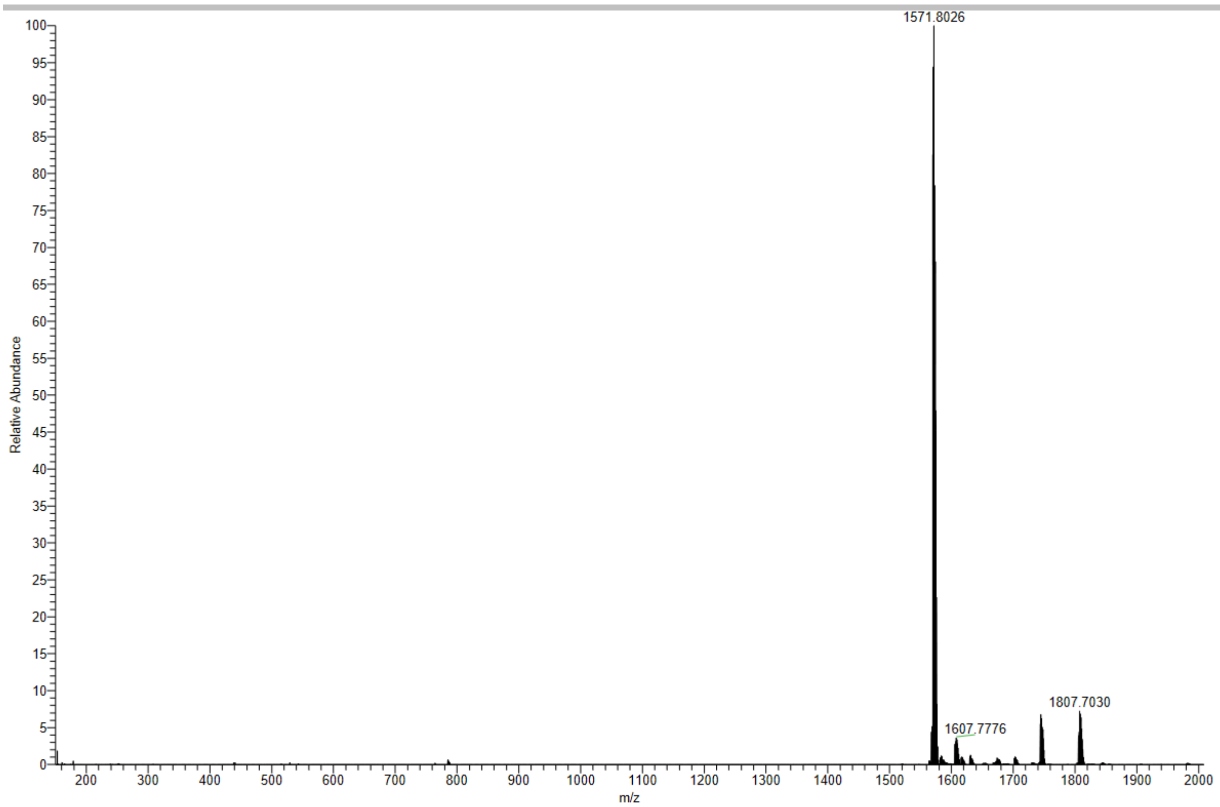

Figure S25. ESI MS of **Zn@2**.

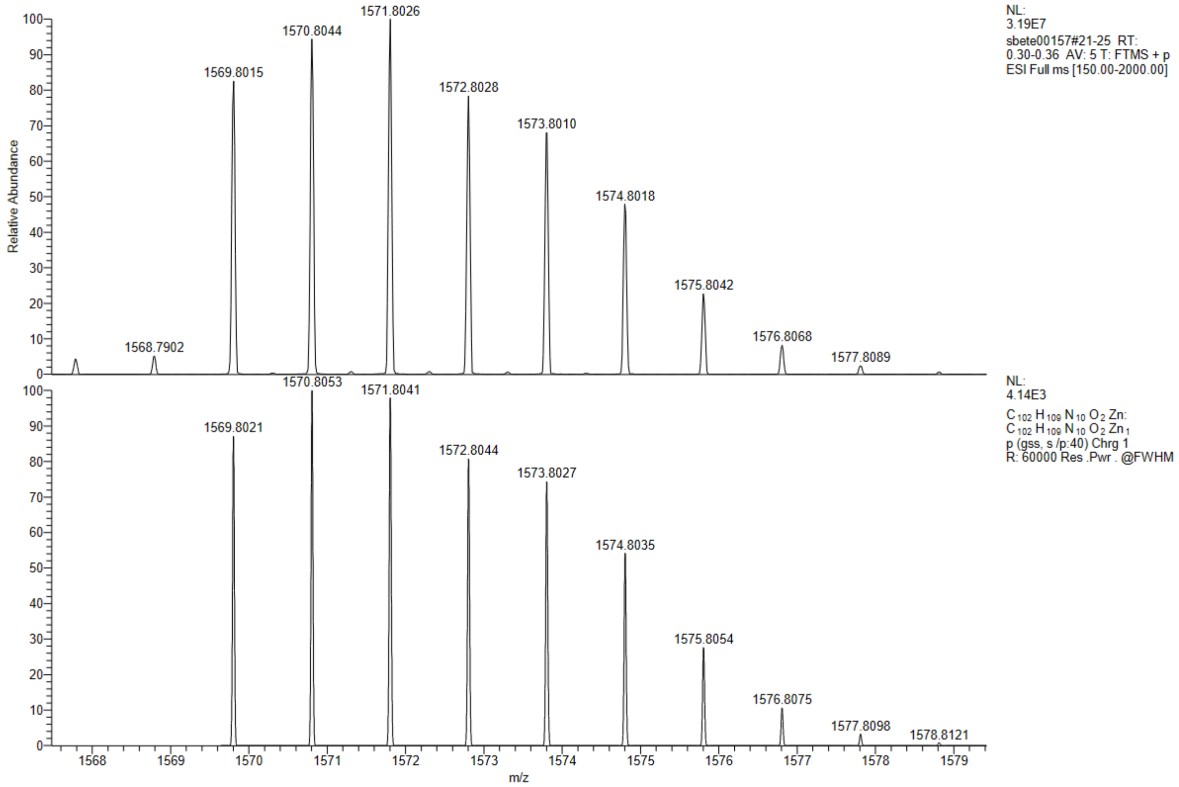

Figure S26. Measured and calculated ESI-MS of **Zn@2** for $[M]^{+}$.

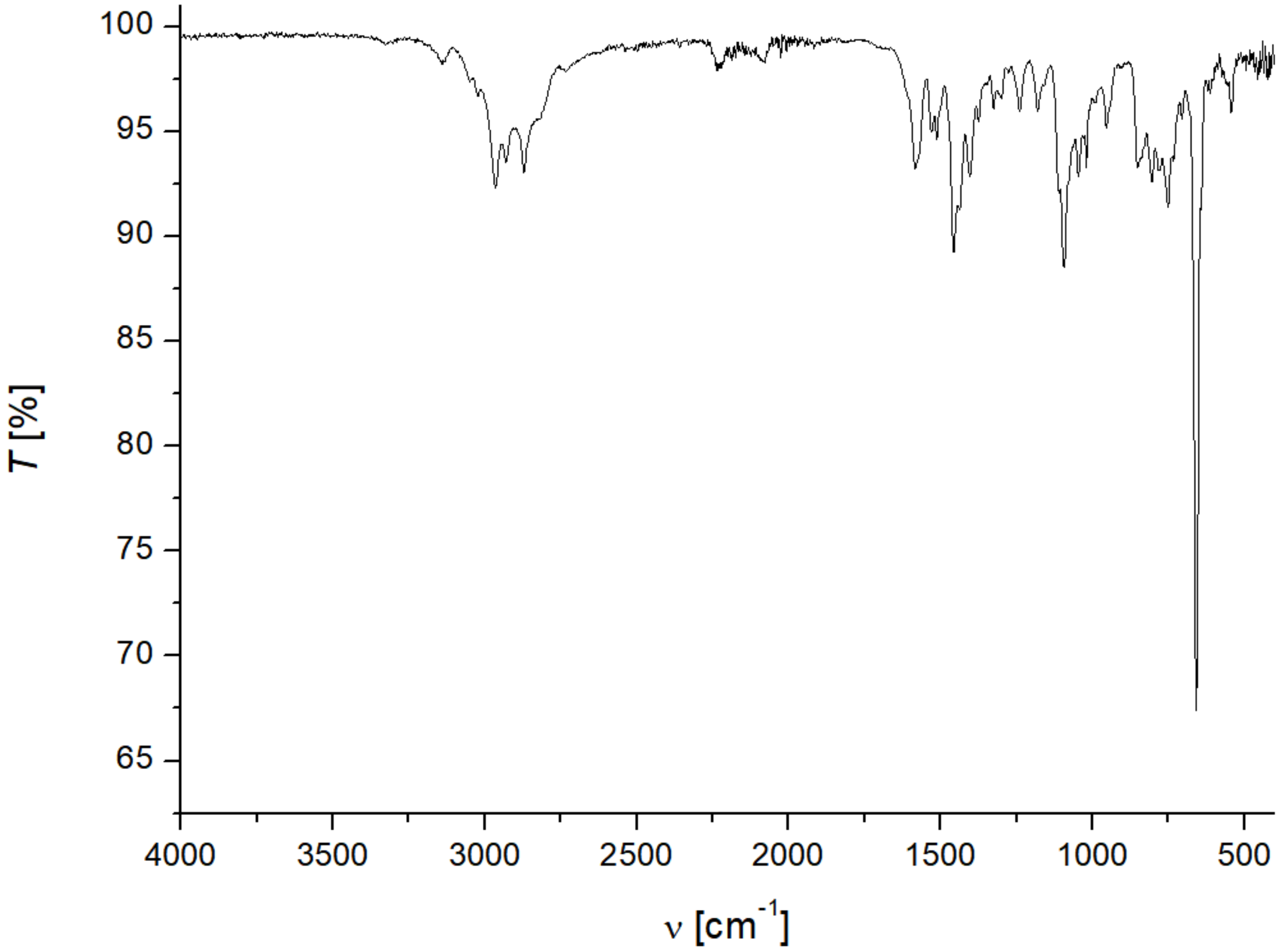

Figure S27. ATR-IR spectrum of **Zn@2**.

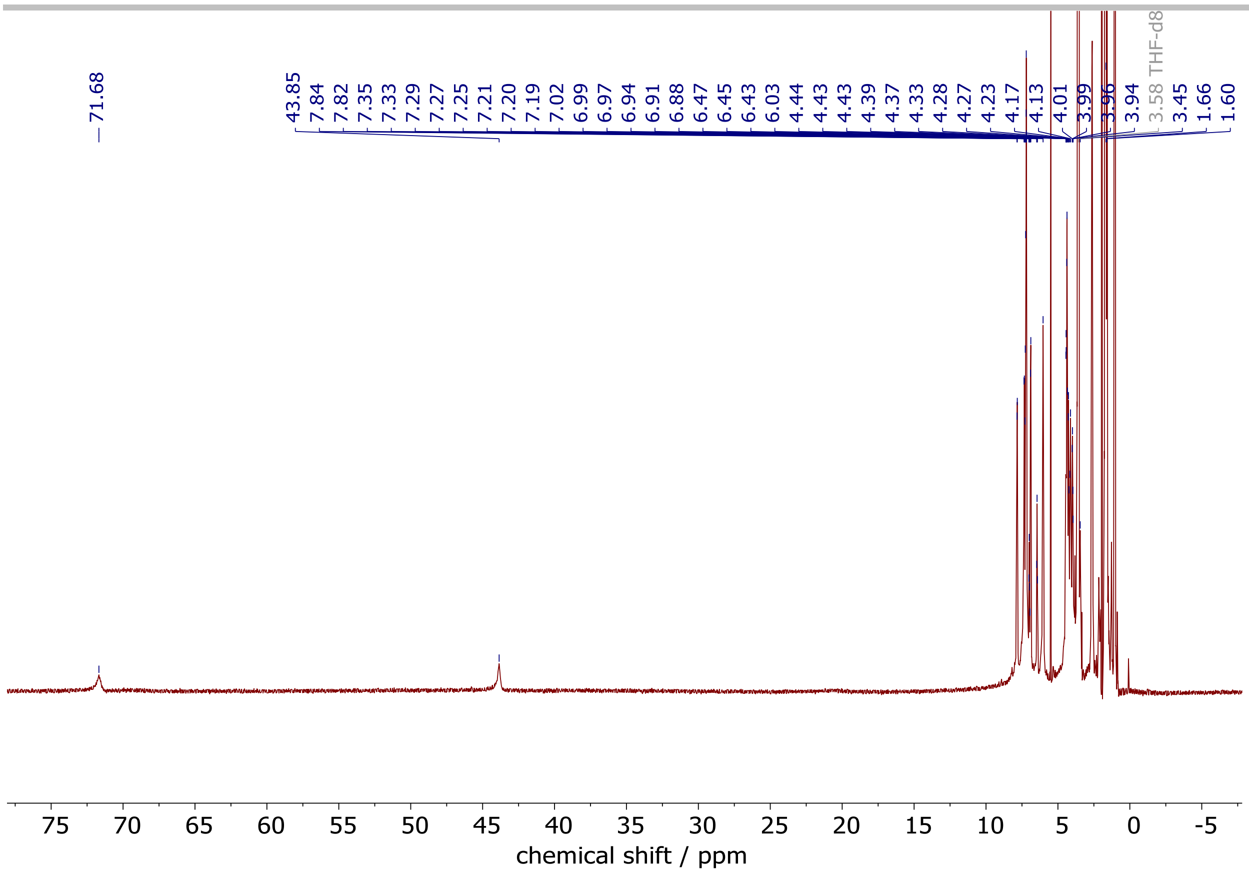

Figure S28. ^{1}H NMR Spectrum of **Fe@2** (method b) in THF-d_8 at rt.

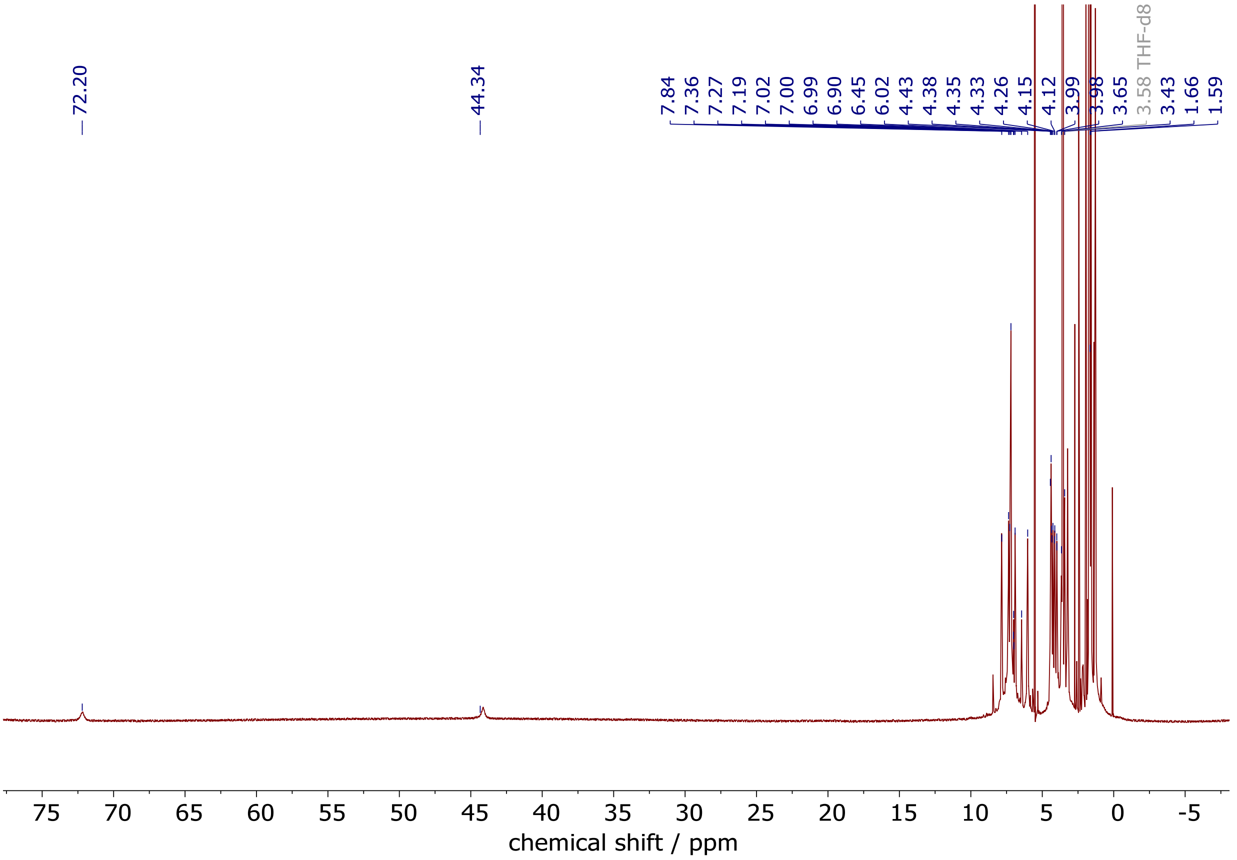

Figure S29. ^{1}H NMR Spectrum of **Fe@2** (method c) in THF-d_8 at rt.

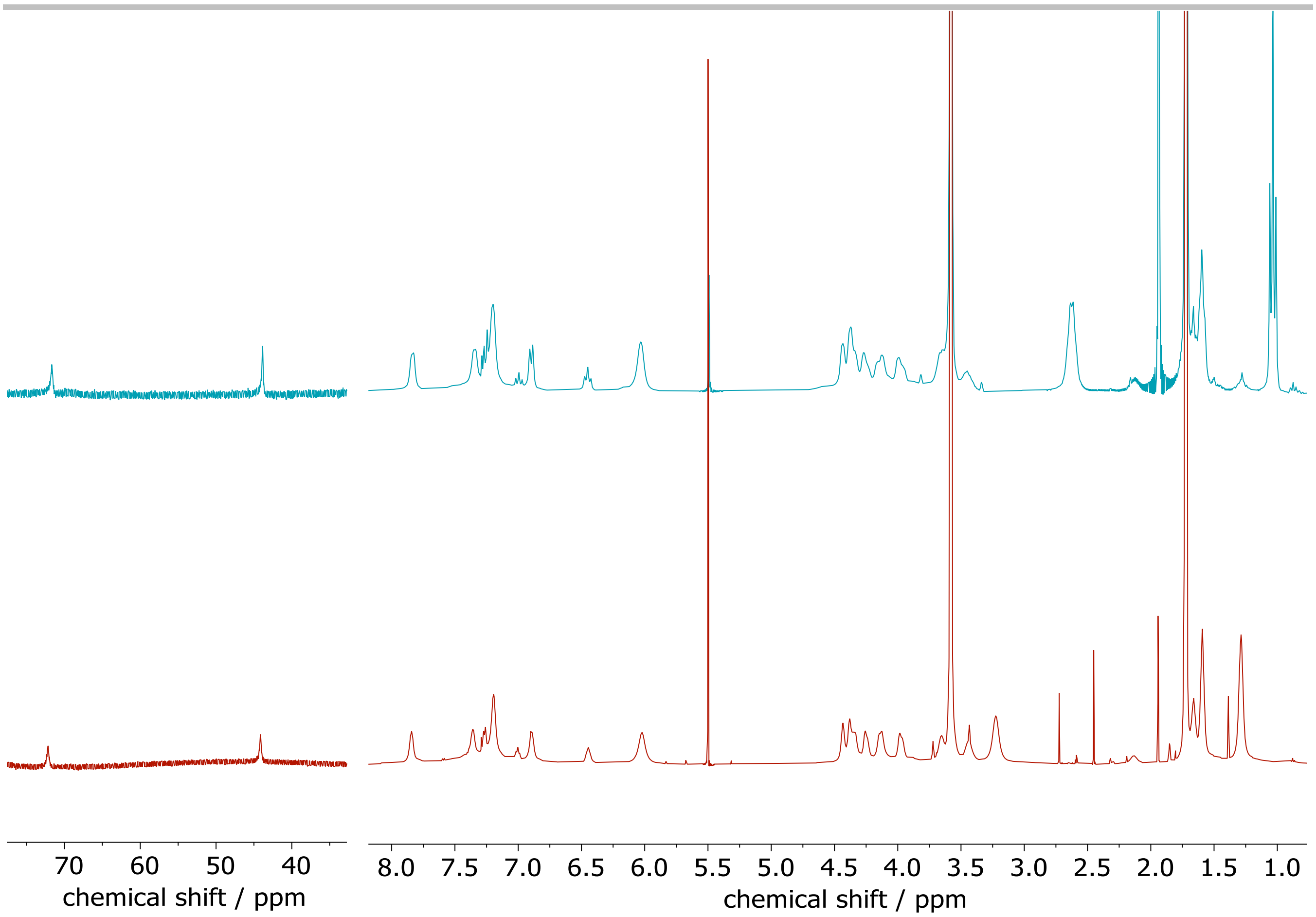

Figure S30. Stack of the ^{1}H NMR Spectra of **Fe@2** after treatment with excess $[Fe(MeCN)_6](SbF_6)_2$ and NEt_3 (method b, top) and synthesized with understoichiometric use of $[Fe(MeCN)_6](SbF_6)_2$ (method c, bottom) in THF-d_8 at rt.

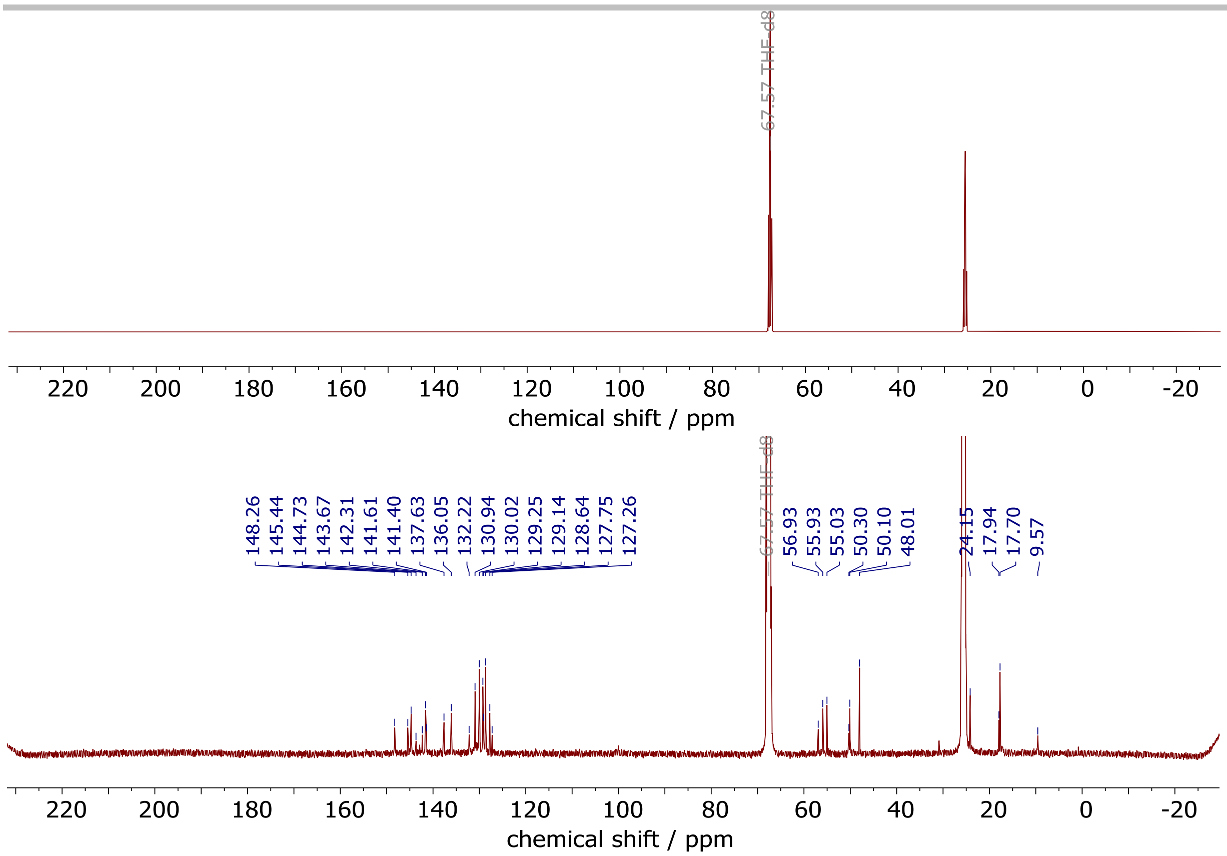

Figure S31. ^{13}C NMR Spectrum of **Fe@2** in THF-d_8 at rt.

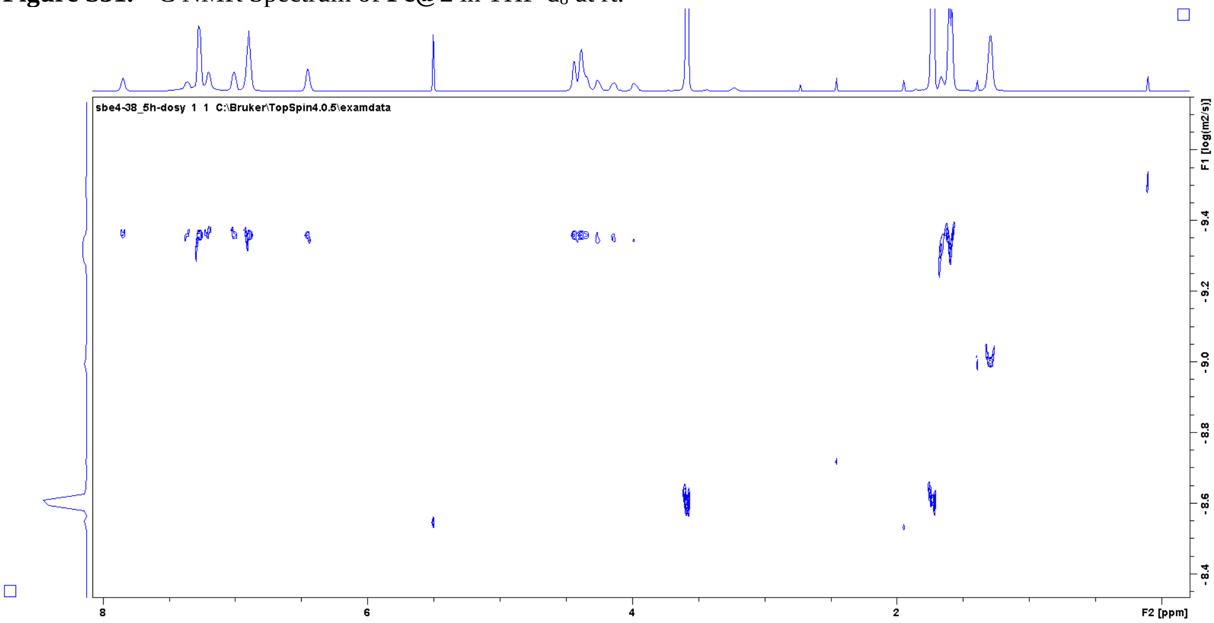

Figure S32. ^{1}H DOSY NMR Spectrum of **Fe@2** in CD_2Cl_2 at rt.

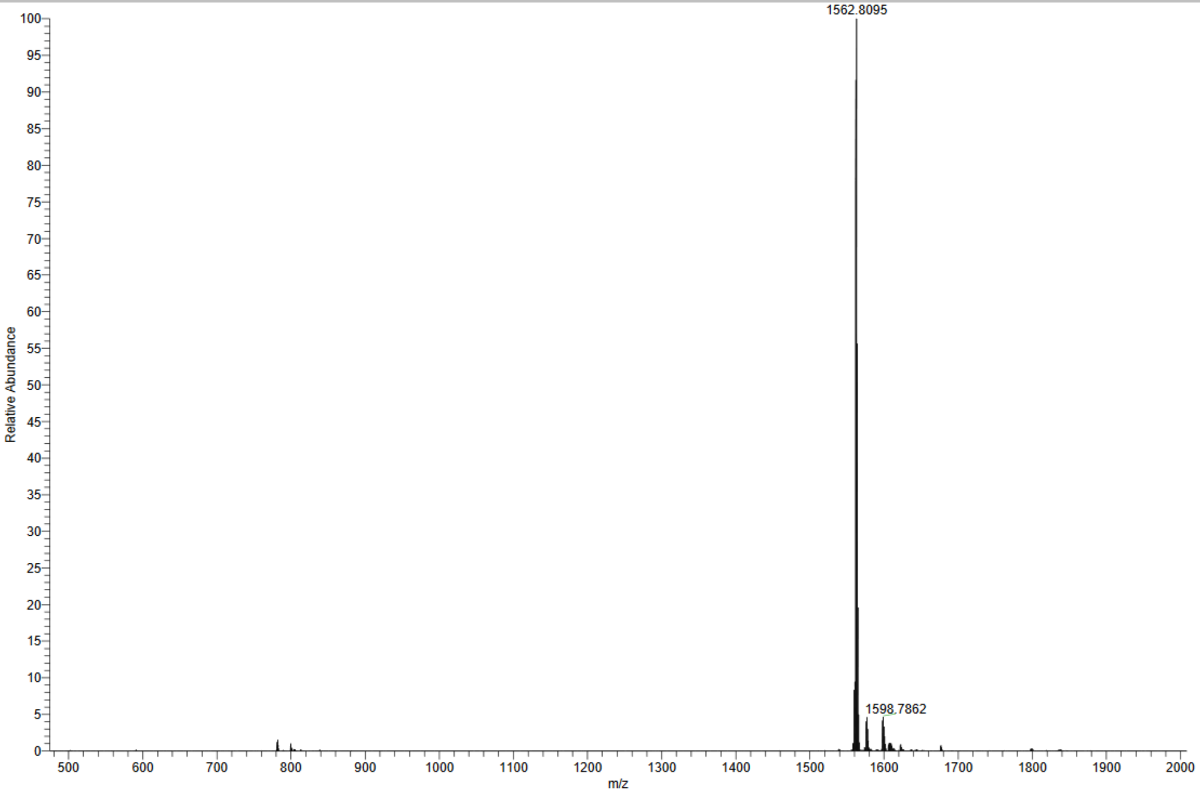

Figure S 1. ESI MS of **Fe@2**.

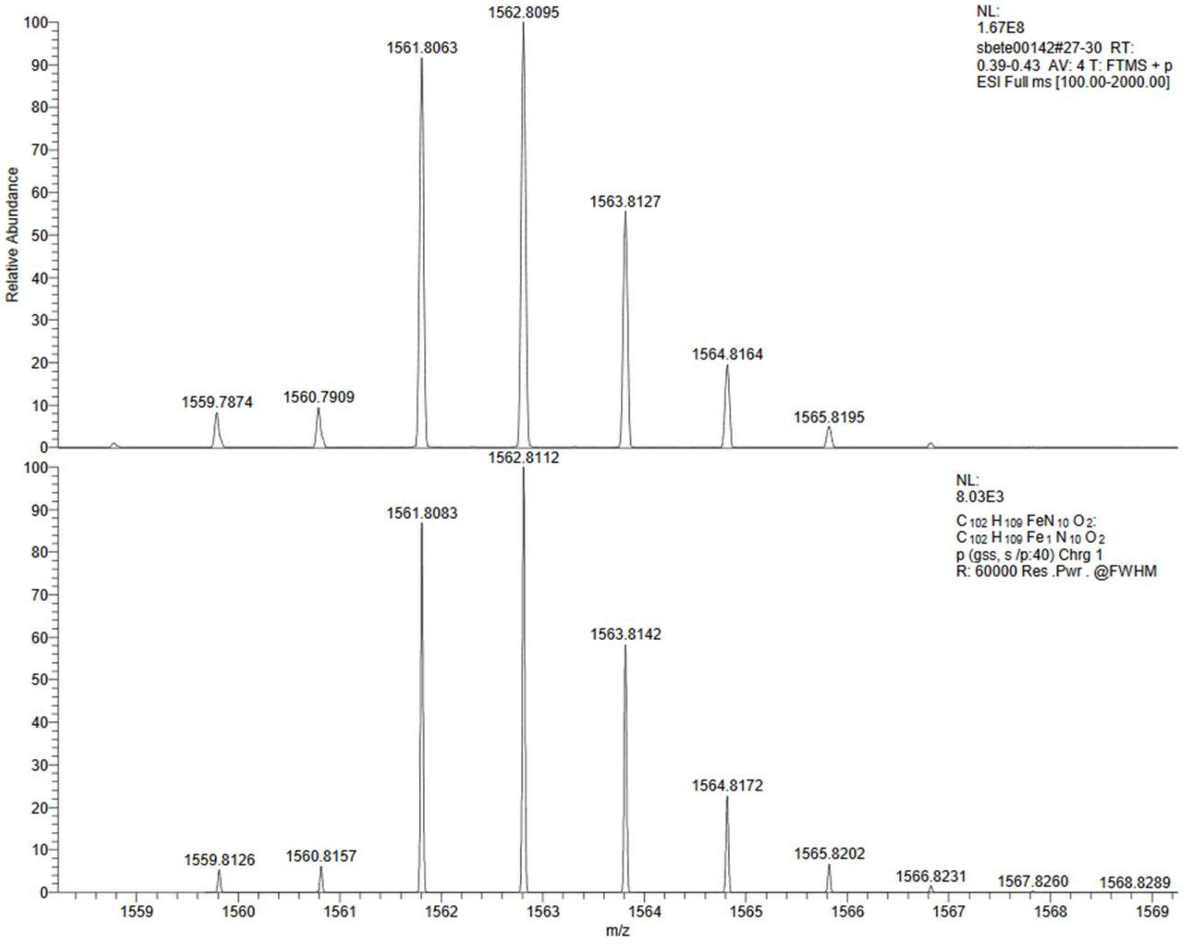

Figure S34. Measured and calculated ESI-MS of **Fe@2** for $[M]^+$.

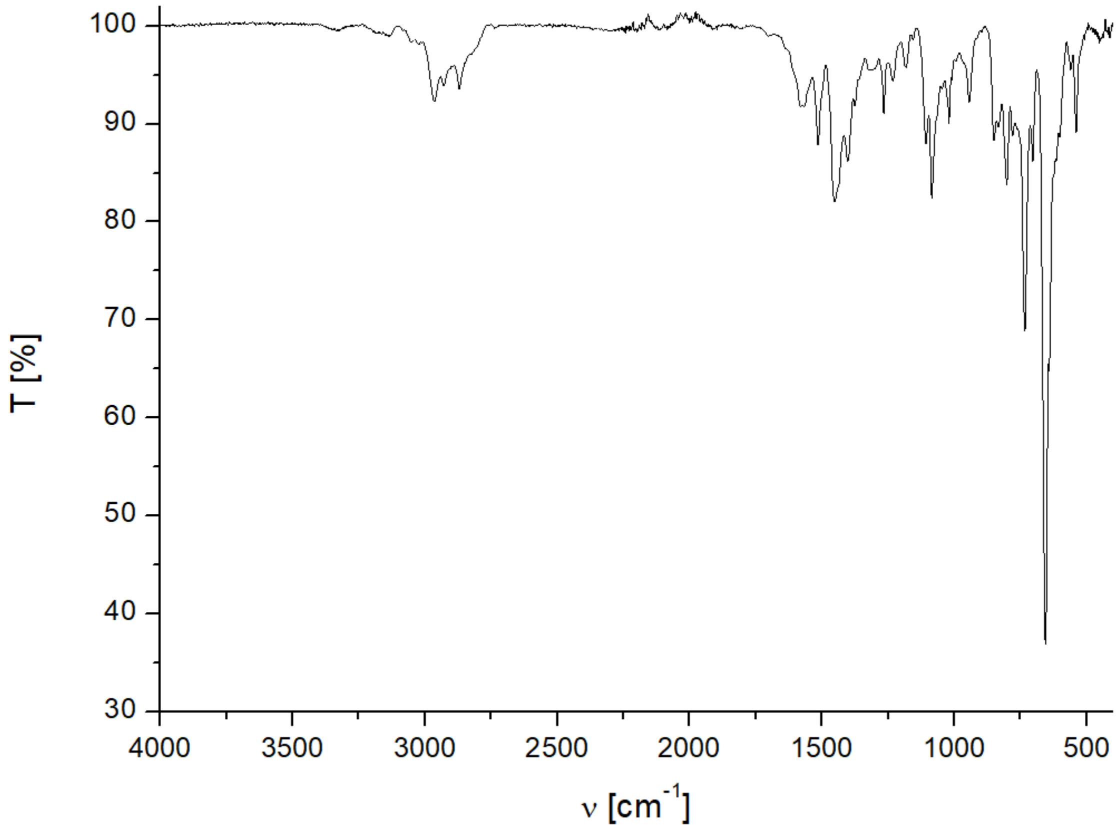

Figure S35. ATR-IR spectrum of **Fe@2.**

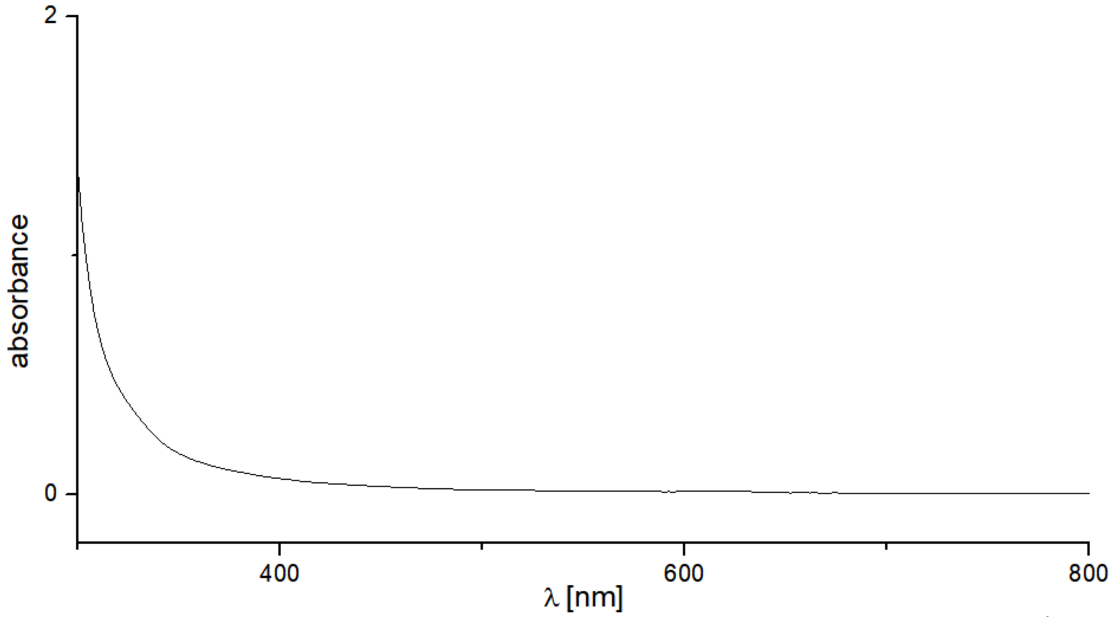

Figure S36. UV/Vis absorption spectrum of **Fe@2** in presence of NEt_3 in THF at rt, c=1.2 · $\mathbf{10^{-6}}$ mM.

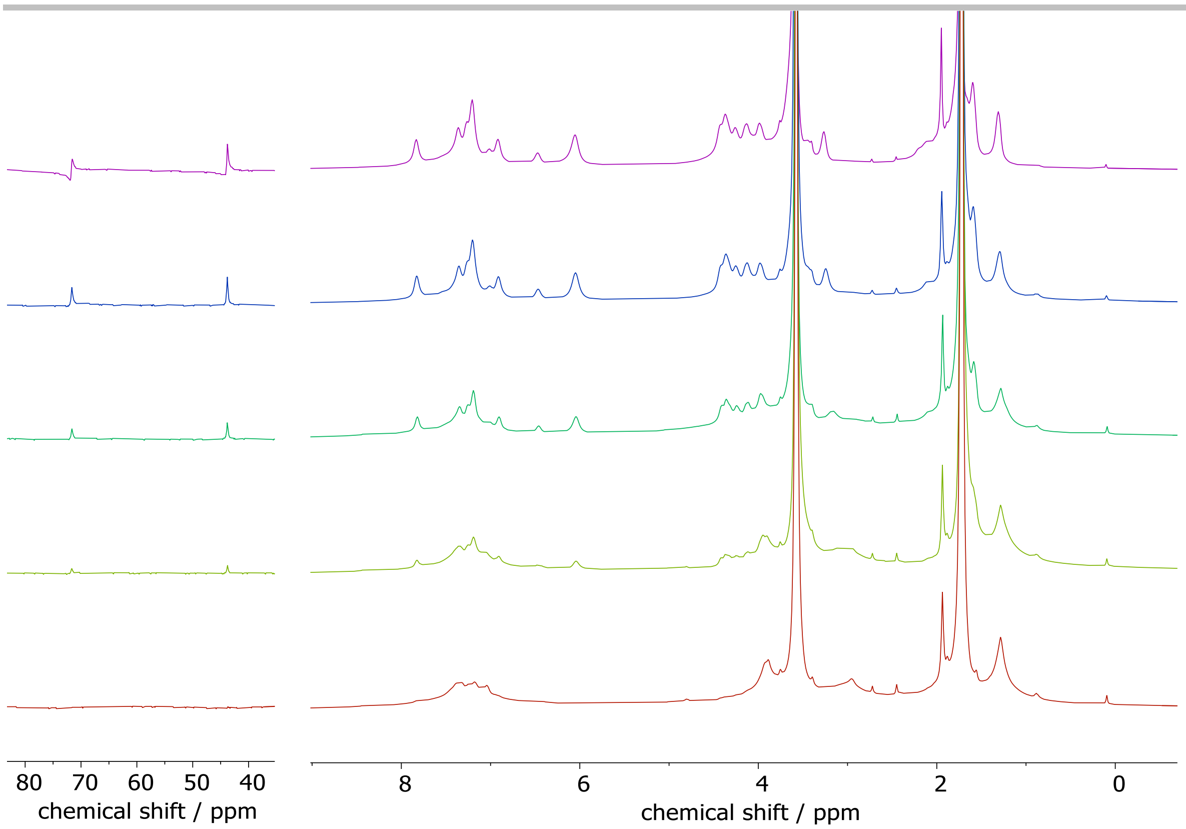

Figure S37. ^{1}H NMR Spectra of **Fe@2** in THF-d_8 at rt, directly after warming up from frozen solution under O_2 atmosphere and after 1, 2, 5 hours reaction time (from top to bottom).

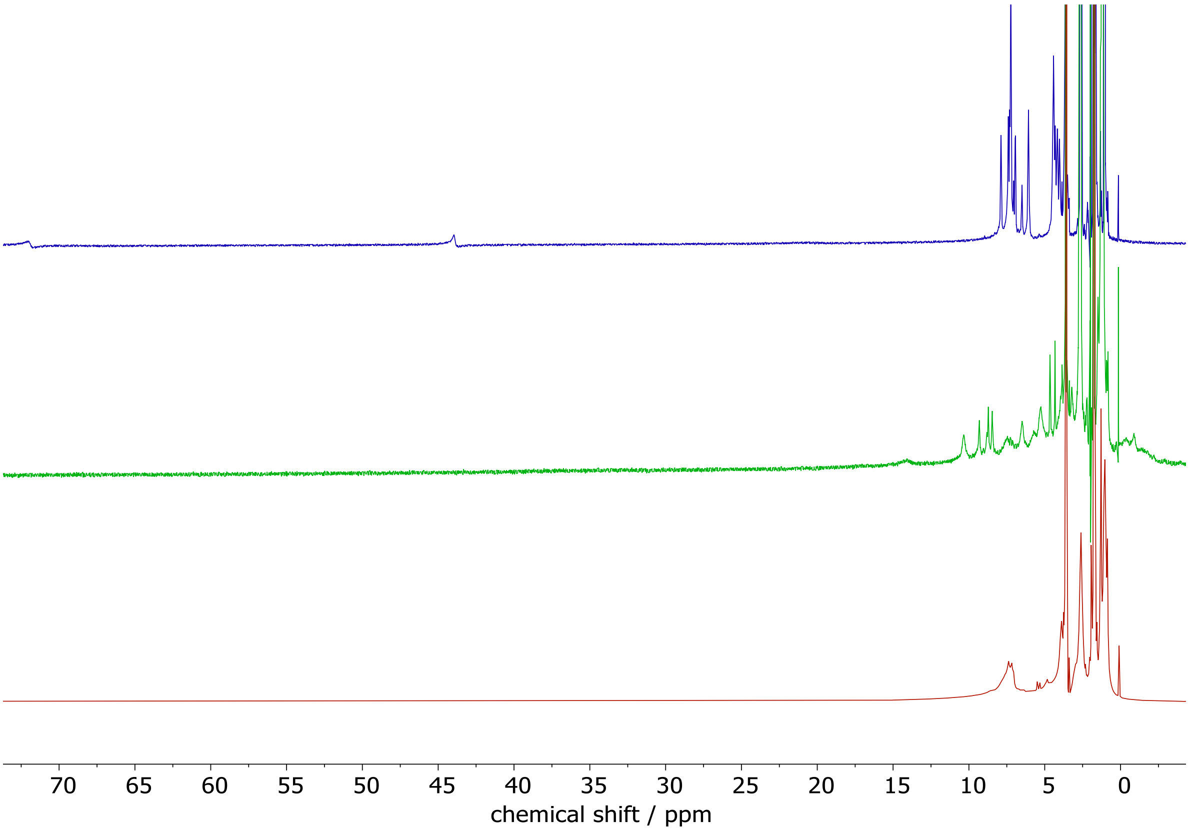

Figure S38. ^{1}H NMR Spectra of **Fe@2** in THF-d_8 at rt (top), after addition of α-ketoglutarate (middle) and directly after warming up from frozen solution under O_2 atmosphere (bottom).

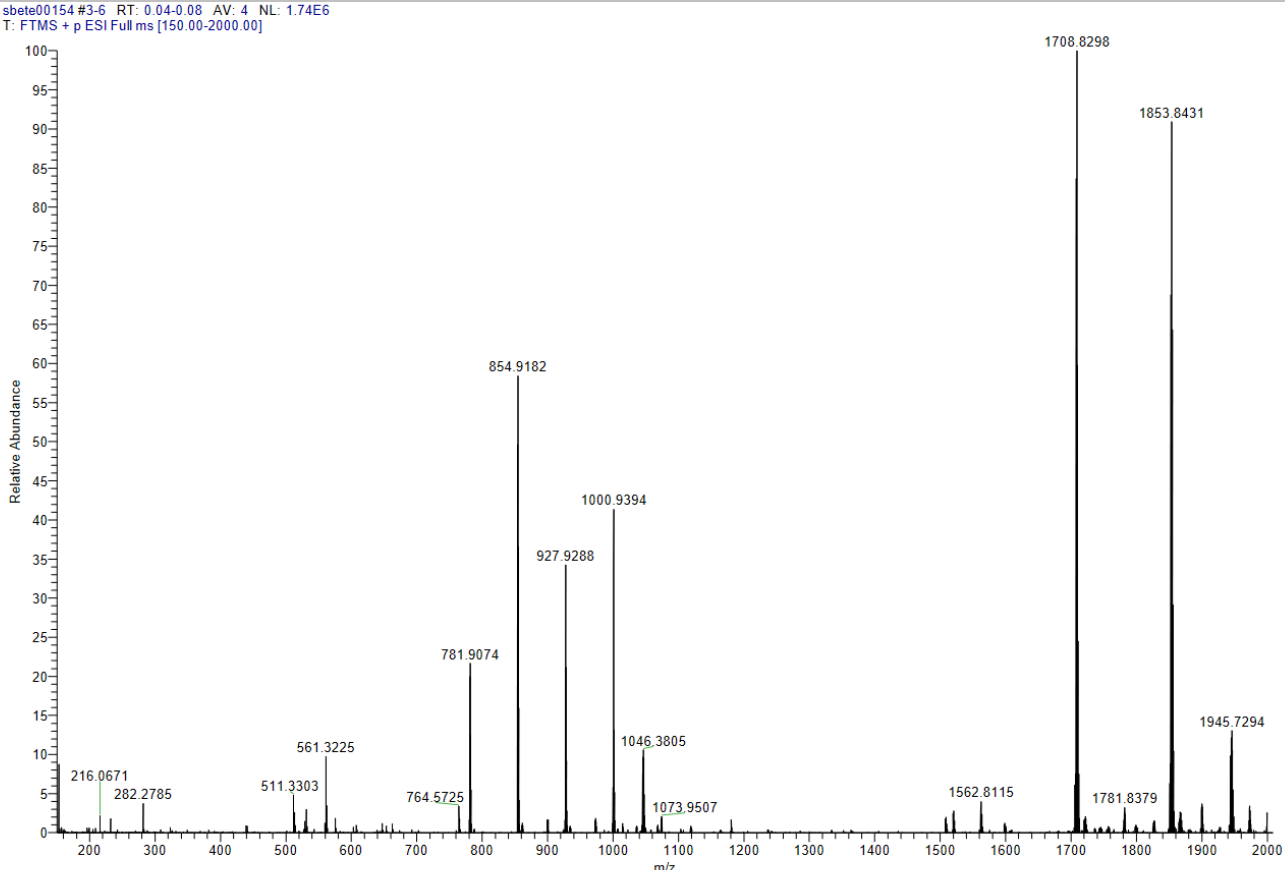

Figure S39. ESI MS of of **Fe@2** in presence of 2.2 eq $\boldsymbol{\alpha}$-ketoglutarate.

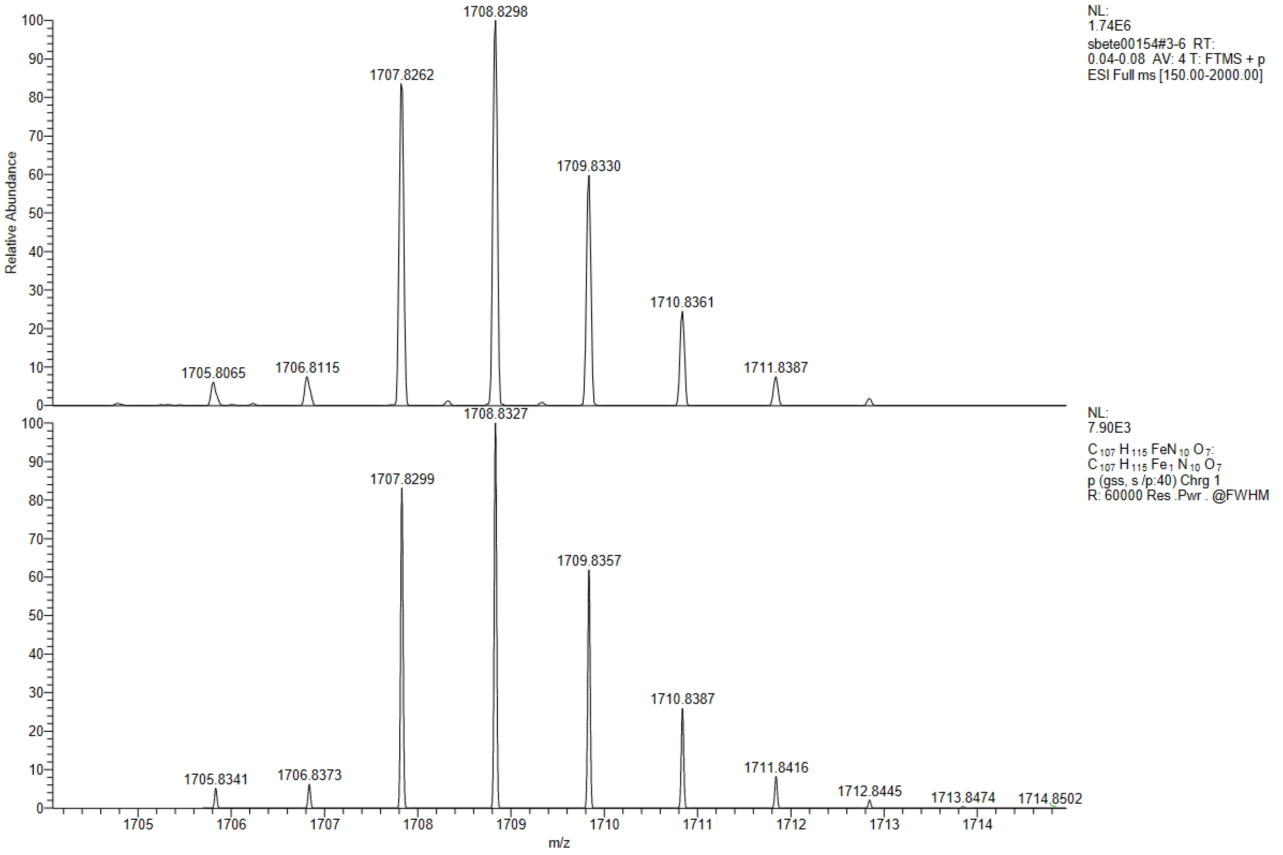

Figure S40. Measured and calculated ESI-MS of **[Fe@2+$\boldsymbol{\alpha}$KG]** for [M]+.

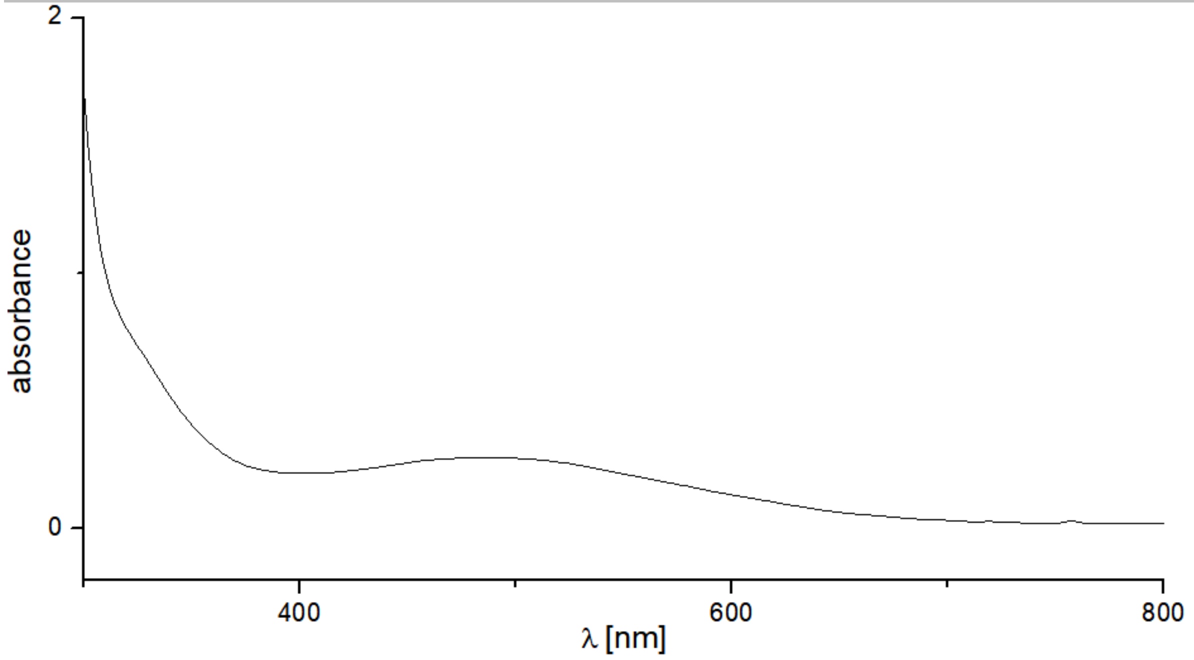

Figure S41. UV/Vis absorption spectrum of **Fe@2** in presence of 10 eq NEt_3 and 2.2 eq $\boldsymbol{\alpha}$-ketoglutarate in THF at rt, $\boldsymbol{c}$=1.2 · $\mathbf{10^{-6}}$ mM.

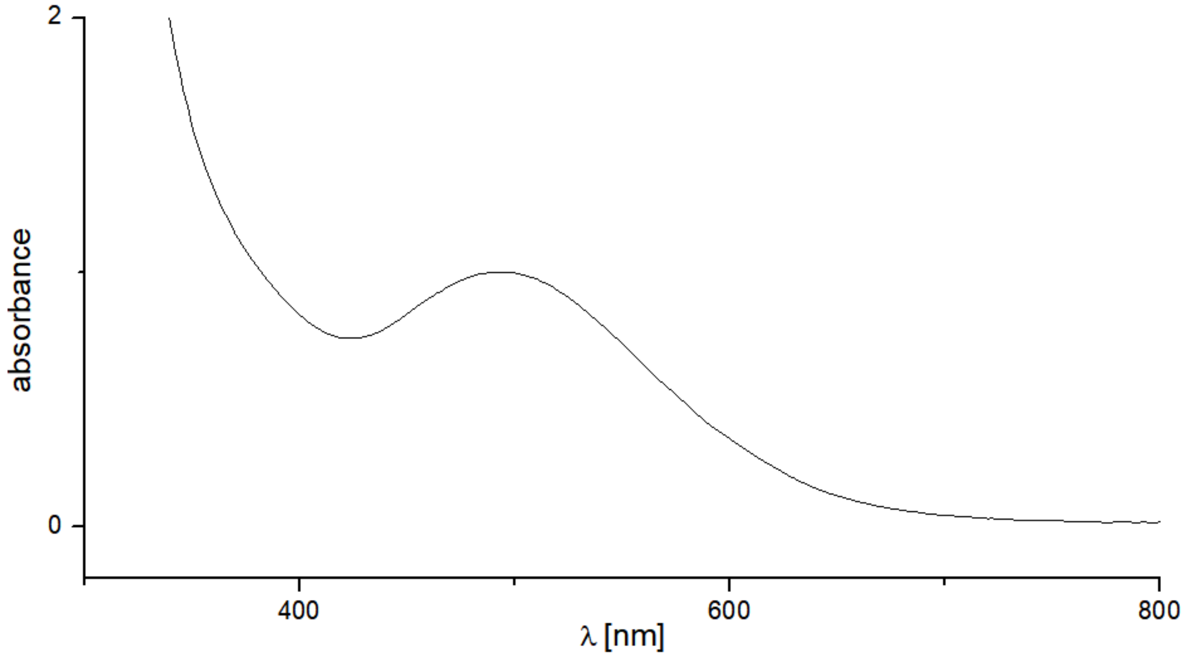

Figure S42. UV/Vis absorption spectrum of **Fe@2** in presence of 10 eq NEt_3 and 2.2 eq $\boldsymbol{\alpha}$-ketoglutarate after warming up from -80 °C to in THF at -20°C, $\boldsymbol{c}$=1.2 · $\mathbf{10^{-6}}$ mM.

X-ray Single-Crystal Structure Analysis

CCDC-2063258 ($Fe(NEt_3)$@2) contain the supplementary crystallographic data for this paper. This data can be obtained free of charge via http://www.ccdc.cam.ac.uk/ products/csd/request/ (or from Cambridge Crystallographic Data Centre, 12 Union Road, Cambridge, CB2 1EZ, UK. Fax: +44-1223-336-033; e-mail: deposit@ccdc.cam.ac.uk)

Crystallographic Details

Suitable single crystals for X-ray structure determination were selected from the mother liquor under an inert gas atmosphere and transferred in protective perfluoro polyether oil on a microscope slide. The selected and mounted crystals were transferred to the cold gas stream on the diffractometer. The diffraction data were obtained at 100 K on a Bruker D8 three-circle diffractometer, equipped with a PHOTON III detector and an INCOATEC microfocus source with Quazar mirror optics (Mo-Kα radiation, λ= 0.71073 Å).

The data obtained were integrated with SAINT and a semi-empirical absorption correction from equivalents with SADABS was applied. The structures were solved and refined using the Bruker SHELX 2014 software package.[7] All non-hydrogen atoms were refined with anisotropic displacement parameters. All C-H hydrogen atoms were refined isotropically on calculated positions by using a riding model with their U_{iso} values constrained to 1.5 U_{eq} of their pivot atoms for terminal sp^3 carbon atoms and 1.2 times for all other atoms.

X-ray Single-Crystal Structure Analysis of Fe@2

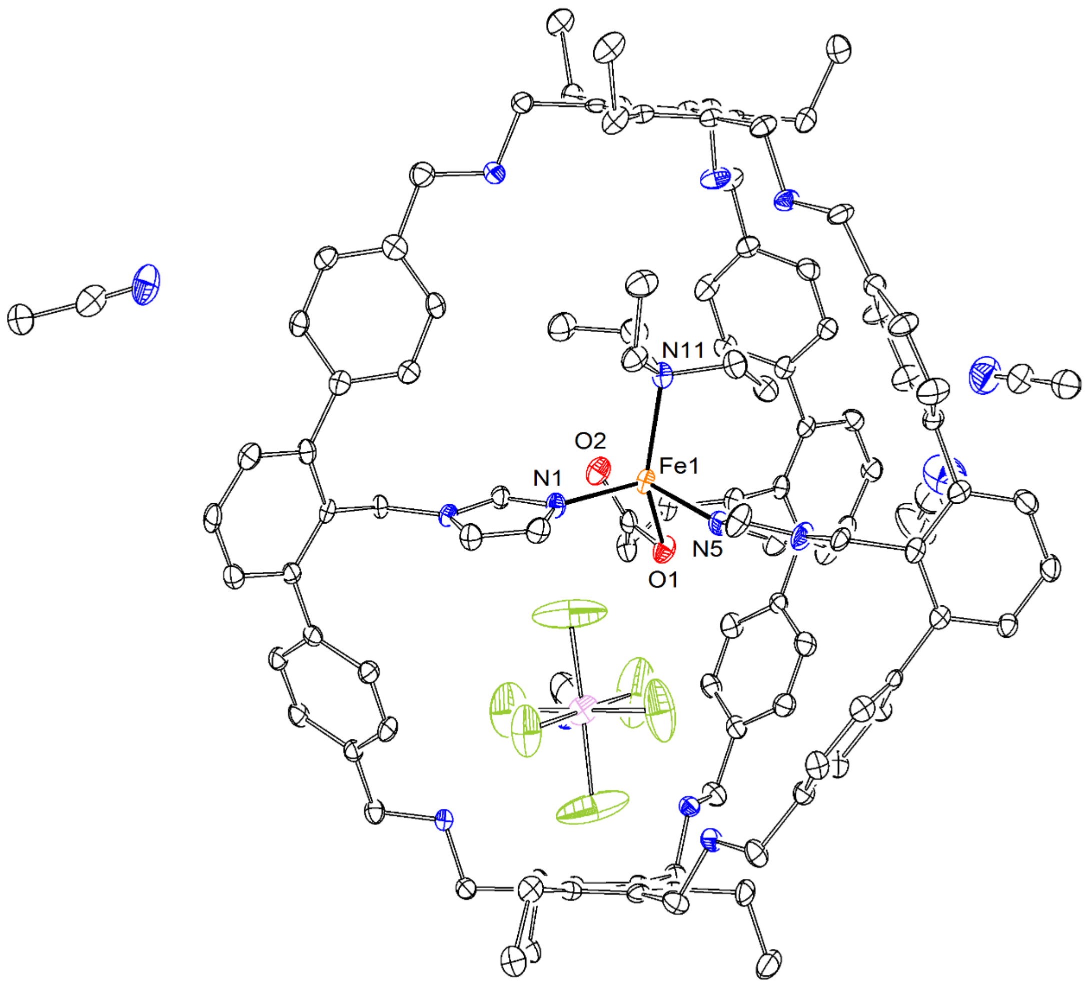

Figure S 2. Thermal ellipsoid plot of **Fe@2** with the anisotropic displacement parameters drawn at the 50% probability level. The asymmetric unit contains one complex molecule, four solvent molecules and one counter ion.

Table S1. Crystal data and structure refinement for **Fe@2**.

Identification code	SB_191220_2_MO	(sbe4-02)
Empirical formula	$C_{116}H_{136}F_6FeN_{15}O_2Sb$	
Formula weight	2063.99	
Temperature	100(2) K	
Wavelength	0.71073 Å	
Crystal system	Triclinic	
Space group	P-1	
Unit cell dimensions	a = 16.5958(4) Å	α= 64.0400(10)°
	b = 18.6880(5) Å	β= 76.1550(10)°
	c = 19.4938(5) Å	γ = 86.568(2)°
Volume	5270.9(2) $Å^3$	
Z	2	
Density (calculated)	1.300 Mg/m^3	
Absorption coefficient	0.463 mm^{-1}	
F(000)	2168	
Crystal size	0.193 x 0.119 x 0.082 mm^3	
Crystal description and colour	Needle, clear pale yellow	
Theta range for data collection	1.798 to 26.098°.	
Index ranges	-20<=h<=20,<br>-23<=k<=23,<br>-24<=l<=24	
Reflections collected	307413	
Independent reflections	20848 [R(int) = 0.1445]	
Completeness to theta = 25.242°	100.0 %	
Refinement method	Full-matrix least-squares on F^2	
Data / restraints / parameters	20848 / 6 / 1307	
Goodness-of-fit on F^2	1.158	
Final R indices [I>2sigma(I)]	R1 = 0.0815,	wR2 = 0.1543
R indices (all data)	R1 = 0.1056,	wR2 = 0.1665
Largest diff. peak and hole	1.034 and -0.742 $eÅ^{-3}$	

Table S2. Bond lengths [Å] and angles [°] for **Fe@2**.

Sb(1)-F(6)	1.848(4)
Sb(1)-F(1)	1.851(4)
Sb(1)-F(4)	1.851(4)
Sb(1)-F(5)	1.854(4)
Sb(1)-F(2)	1.858(4)
Sb(1)-F(3)	1.869(3)
Fe(1)-O(1)	2.062(3)
Fe(1)-N(1)	2.065(4)
Fe(1)-N(5)	2.102(4)
Fe(1)-N(11)	2.148(4)
Fe(1)-O(2)	2.289(3)
O(1)-C(49)	1.278(6)
O(2)-C(49)	1.248(6)
N(1)-C(1)	1.325(6)
N(1)-C(2)	1.379(6)
N(2)-C(1)	1.335(6)
N(2)-C(3)	1.371(6)
N(2)-C(4)	1.486(5)
N(3)-C(79)	1.457(6)
N(3)-C(17)	1.457(6)
N(3)-H(803)	0.98(2)
N(4)-C(24)	1.451(6)
N(4)-C(94)	1.463(6)
N(4)-H(804)	0.98(2)
N(5)-C(25)	1.300(6)
N(5)-C(26)	1.411(7)
N(6)-C(25)	1.312(6)
N(6)-C(27)	1.417(7)
N(6)-C(28)	1.490(6)
N(7)-C(41)	1.459(6)
N(7)-C(85)	1.471(6)
N(7)-H(807)	0.973(19)
N(8)-C(48)	1.463(6)
N(8)-C(100)	1.466(6)

N(8)-H(808)	0.98(2)
N(9)-C(82)	1.460(6)
N(9)-C(65)	1.466(6)
N(9)-H(809)	0.979(19)
N(10)-C(72)	1.455(6)
N(10)-C(97)	1.478(6)
N(10)-H(810)	0.99(2)
N(11)-C(103)	1.488(6)
N(11)-C(107)	1.498(6)
N(11)-C(105)	1.508(6)
N(12)-C(109)	1.152(9)
N(13)-C(111)	1.180(9)
N(14)-C(113)	1.158(9)
N(15)-C(115)	1.141(7)
C(2)-C(3)	1.356(7)
C(4)-C(5)	1.500(6)
C(5)-C(6)	1.410(6)
C(5)-C(10)	1.415(6)
C(6)-C(7)	1.396(6)
C(6)-C(11)	1.489(6)
C(7)-C(8)	1.386(7)
C(8)-C(9)	1.377(7)
C(9)-C(10)	1.401(6)
C(10)-C(18)	1.489(6)
C(11)-C(12)	1.381(6)
C(11)-C(16)	1.396(6)
C(12)-C(13)	1.394(7)
C(13)-C(14)	1.386(6)
C(14)-C(15)	1.397(6)
C(14)-C(17)	1.499(6)
C(15)-C(16)	1.383(7)
C(18)-C(23)	1.400(6)
C(18)-C(19)	1.413(6)
C(19)-C(20)	1.369(7)
C(20)-C(21)	1.400(7)
C(21)-C(22)	1.389(7)
C(21)-C(24)	1.504(7)

C(22)-C(23)	1.394(6)
C(26)-C(27)	1.357(7)
C(28)-C(29)	1.513(6)
C(29)-C(34)	1.401(6)
C(29)-C(30)	1.406(7)
C(30)-C(31)	1.390(7)
C(30)-C(42)	1.495(6)
C(31)-C(32)	1.380(7)
C(32)-C(33)	1.386(7)
C(33)-C(34)	1.398(6)
C(34)-C(35)	1.500(6)
C(35)-C(40)	1.386(6)
C(35)-C(36)	1.389(6)
C(36)-C(37)	1.384(7)
C(37)-C(38)	1.383(6)
C(38)-C(39)	1.388(7)
C(38)-C(41)	1.505(6)
C(39)-C(40)	1.374(6)
C(42)-C(43)	1.382(6)
C(42)-C(47)	1.396(7)
C(43)-C(44)	1.391(7)
C(44)-C(45)	1.385(7)
C(45)-C(46)	1.376(7)
C(45)-C(48)	1.506(6)
C(46)-C(47)	1.388(7)
C(49)-C(50)	1.509(7)
C(50)-C(51)	1.536(7)
C(51)-C(52)	1.537(6)
C(52)-C(53)	1.516(6)
C(53)-C(58)	1.405(6)
C(53)-C(54)	1.408(7)
C(54)-C(55)	1.396(7)
C(54)-C(66)	1.502(6)
C(55)-C(56)	1.381(7)
C(56)-C(57)	1.383(7)
C(57)-C(58)	1.392(6)
C(58)-C(59)	1.503(6)

C(59)-C(64)	1.380(7)
C(59)-C(60)	1.395(7)
C(60)-C(61)	1.369(7)
C(61)-C(62)	1.387(7)
C(62)-C(63)	1.383(7)
C(62)-C(65)	1.509(7)
C(63)-C(64)	1.389(7)
C(66)-C(67)	1.381(7)
C(66)-C(71)	1.393(7)
C(67)-C(68)	1.389(7)
C(68)-C(69)	1.386(7)
C(69)-C(70)	1.392(7)
C(69)-C(72)	1.516(6)
C(70)-C(71)	1.392(7)
C(73)-C(78)	1.406(6)
C(73)-C(74)	1.411(6)
C(73)-C(79)	1.514(6)
C(74)-C(75)	1.402(6)
C(74)-C(80)	1.509(6)
C(75)-C(76)	1.397(6)
C(75)-C(82)	1.518(6)
C(76)-C(77)	1.407(7)
C(76)-C(83)	1.516(6)
C(77)-C(78)	1.403(7)
C(77)-C(85)	1.512(6)
C(78)-C(86)	1.519(6)
C(80)-C(81)	1.537(7)
C(83)-C(84)	1.532(7)
C(86)-C(87)	1.531(7)
C(88)-C(89)	1.397(6)
C(88)-C(93)	1.407(6)
C(88)-C(94)	1.518(6)
C(89)-C(90)	1.401(6)
C(89)-C(95)	1.523(6)
C(90)-C(91)	1.410(6)
C(90)-C(97)	1.518(6)
C(91)-C(92)	1.402(6)

C(91)-C(98)	1.519(6)
C(92)-C(93)	1.402(6)
C(92)-C(100)	1.519(6)
C(93)-C(101)	1.516(6)
C(95)-C(96)	1.527(7)
C(98)-C(99)	1.534(7)
C(101)-C(102)	1.526(7)
C(103)-C(104)	1.519(7)
C(105)-C(106)	1.508(7)
C(107)-C(108)	1.503(7)
C(109)-C(110)	1.445(10)
C(111)-C(112)	1.468(11)
C(113)-C(114)	1.461(10)
C(115)-C(116)	1.460(8)
F(6)-Sb(1)-F(1)	92.9(3)
F(6)-Sb(1)-F(4)	89.9(2)
F(1)-Sb(1)-F(4)	87.8(2)
F(6)-Sb(1)-F(5)	176.8(3)
F(1)-Sb(1)-F(5)	90.3(3)
F(4)-Sb(1)-F(5)	90.0(2)
F(6)-Sb(1)-F(2)	90.2(3)
F(1)-Sb(1)-F(2)	91.86(19)
F(4)-Sb(1)-F(2)	179.7(2)
F(5)-Sb(1)-F(2)	89.9(2)
F(6)-Sb(1)-F(3)	88.5(2)
F(1)-Sb(1)-F(3)	178.5(2)
F(4)-Sb(1)-F(3)	91.51(17)
F(5)-Sb(1)-F(3)	88.4(2)
F(2)-Sb(1)-F(3)	88.78(16)
O(1)-Fe(1)-N(1)	107.65(15)
O(1)-Fe(1)-N(5)	91.13(15)
N(1)-Fe(1)-N(5)	108.07(16)
O(1)-Fe(1)-N(11)	136.48(15)
N(1)-Fe(1)-N(11)	108.08(15)
N(5)-Fe(1)-N(11)	100.57(16)
O(1)-Fe(1)-O(2)	60.10(12)

N(1)-Fe(1)-O(2)	89.54(14)
N(5)-Fe(1)-O(2)	150.18(15)
N(11)-Fe(1)-O(2)	96.29(14)
C(49)-O(1)-Fe(1)	94.7(3)
C(49)-O(2)-Fe(1)	85.2(3)
C(1)-N(1)-C(2)	105.4(4)
C(1)-N(1)-Fe(1)	123.9(3)
C(2)-N(1)-Fe(1)	130.7(3)
C(1)-N(2)-C(3)	107.3(4)
C(1)-N(2)-C(4)	122.8(4)
C(3)-N(2)-C(4)	129.7(4)
C(79)-N(3)-C(17)	115.1(4)
C(79)-N(3)-H(803)	111(3)
C(17)-N(3)-H(803)	111(3)
C(24)-N(4)-C(94)	113.4(4)
C(24)-N(4)-H(804)	113(5)
C(94)-N(4)-H(804)	103(5)
C(25)-N(5)-C(26)	104.6(4)
C(25)-N(5)-Fe(1)	137.4(4)
C(26)-N(5)-Fe(1)	114.1(3)
C(25)-N(6)-C(27)	106.1(4)
C(25)-N(6)-C(28)	127.3(4)
C(27)-N(6)-C(28)	126.6(4)
C(41)-N(7)-C(85)	111.5(4)
C(41)-N(7)-H(807)	110(3)
C(85)-N(7)-H(807)	109(3)
C(48)-N(8)-C(100)	113.6(4)
C(48)-N(8)-H(808)	107(3)
C(100)-N(8)-H(808)	109(3)
C(82)-N(9)-C(65)	113.2(4)
C(82)-N(9)-H(809)	110(3)
C(65)-N(9)-H(809)	109(3)
C(72)-N(10)-C(97)	112.2(4)
C(72)-N(10)-H(810)	113(8)
C(97)-N(10)-H(810)	110(8)
C(103)-N(11)-C(107)	111.8(4)
C(103)-N(11)-C(105)	109.5(4)

C(107)-N(11)-C(105)	108.2(4)
C(103)-N(11)-Fe(1)	104.9(3)
C(107)-N(11)-Fe(1)	114.7(3)
C(105)-N(11)-Fe(1)	107.6(3)
N(1)-C(1)-N(2)	111.6(4)
C(3)-C(2)-N(1)	109.4(4)
C(2)-C(3)-N(2)	106.4(4)
N(2)-C(4)-C(5)	114.2(4)
C(6)-C(5)-C(10)	119.7(4)
C(6)-C(5)-C(4)	118.8(4)
C(10)-C(5)-C(4)	121.0(4)
C(7)-C(6)-C(5)	119.9(4)
C(7)-C(6)-C(11)	119.0(4)
C(5)-C(6)-C(11)	121.1(4)
C(8)-C(7)-C(6)	120.2(4)
C(9)-C(8)-C(7)	120.1(4)
C(8)-C(9)-C(10)	121.7(4)
C(9)-C(10)-C(5)	118.3(4)
C(9)-C(10)-C(18)	117.5(4)
C(5)-C(10)-C(18)	124.2(4)
C(12)-C(11)-C(16)	118.4(4)
C(12)-C(11)-C(6)	121.6(4)
C(16)-C(11)-C(6)	120.1(4)
C(11)-C(12)-C(13)	120.9(4)
C(14)-C(13)-C(12)	121.4(4)
C(13)-C(14)-C(15)	117.0(4)
C(13)-C(14)-C(17)	122.7(4)
C(15)-C(14)-C(17)	120.2(4)
C(16)-C(15)-C(14)	122.0(4)
C(15)-C(16)-C(11)	120.2(4)
N(3)-C(17)-C(14)	110.9(4)
C(23)-C(18)-C(19)	117.1(4)
C(23)-C(18)-C(10)	124.0(4)
C(19)-C(18)-C(10)	118.6(4)
C(20)-C(19)-C(18)	121.2(4)
C(19)-C(20)-C(21)	121.6(4)
C(22)-C(21)-C(20)	117.7(4)

C(22)-C(21)-C(24)	122.1(4)
C(20)-C(21)-C(24)	120.1(4)
C(21)-C(22)-C(23)	121.2(4)
C(22)-C(23)-C(18)	121.1(4)
N(4)-C(24)-C(21)	112.8(4)
N(5)-C(25)-N(6)	114.6(5)
C(27)-C(26)-N(5)	109.1(5)
C(26)-C(27)-N(6)	105.6(4)
N(6)-C(28)-C(29)	109.6(4)
C(34)-C(29)-C(30)	119.4(4)
C(34)-C(29)-C(28)	120.7(4)
C(30)-C(29)-C(28)	119.7(4)
C(31)-C(30)-C(29)	119.4(4)
C(31)-C(30)-C(42)	120.4(4)
C(29)-C(30)-C(42)	120.2(4)
C(32)-C(31)-C(30)	121.3(5)
C(31)-C(32)-C(33)	119.6(4)
C(32)-C(33)-C(34)	120.3(4)
C(33)-C(34)-C(29)	119.9(4)
C(33)-C(34)-C(35)	118.6(4)
C(29)-C(34)-C(35)	121.3(4)
C(40)-C(35)-C(36)	118.0(4)
C(40)-C(35)-C(34)	119.7(4)
C(36)-C(35)-C(34)	122.3(4)
C(37)-C(36)-C(35)	120.8(4)
C(38)-C(37)-C(36)	120.9(4)
C(37)-C(38)-C(39)	118.0(4)
C(37)-C(38)-C(41)	122.0(4)
C(39)-C(38)-C(41)	120.0(4)
C(40)-C(39)-C(38)	121.2(4)
C(39)-C(40)-C(35)	121.0(4)
N(7)-C(41)-C(38)	111.9(4)
C(43)-C(42)-C(47)	118.1(4)
C(43)-C(42)-C(30)	121.7(4)
C(47)-C(42)-C(30)	120.1(4)
C(42)-C(43)-C(44)	120.4(5)
C(45)-C(44)-C(43)	121.5(4)

C(46)-C(45)-C(44)	118.0(4)
C(46)-C(45)-C(48)	120.2(4)
C(44)-C(45)-C(48)	121.8(4)
C(45)-C(46)-C(47)	121.1(5)
C(46)-C(47)-C(42)	120.7(4)
N(8)-C(48)-C(45)	111.7(4)
O(2)-C(49)-O(1)	120.0(4)
O(2)-C(49)-C(50)	120.9(4)
O(1)-C(49)-C(50)	119.1(4)
C(49)-C(50)-C(51)	109.7(4)
C(50)-C(51)-C(52)	112.6(4)
C(53)-C(52)-C(51)	115.1(4)
C(58)-C(53)-C(54)	119.0(4)
C(58)-C(53)-C(52)	119.1(4)
C(54)-C(53)-C(52)	121.8(4)
C(55)-C(54)-C(53)	119.4(4)
C(55)-C(54)-C(66)	116.6(4)
C(53)-C(54)-C(66)	124.1(4)
C(56)-C(55)-C(54)	121.0(5)
C(55)-C(56)-C(57)	119.8(5)
C(56)-C(57)-C(58)	120.4(4)
C(57)-C(58)-C(53)	120.3(4)
C(57)-C(58)-C(59)	117.9(4)
C(53)-C(58)-C(59)	121.7(4)
C(64)-C(59)-C(60)	118.0(4)
C(64)-C(59)-C(58)	121.9(4)
C(60)-C(59)-C(58)	120.1(4)
C(61)-C(60)-C(59)	121.1(5)
C(60)-C(61)-C(62)	121.5(5)
C(63)-C(62)-C(61)	117.4(4)
C(63)-C(62)-C(65)	123.0(4)
C(61)-C(62)-C(65)	119.6(4)
C(62)-C(63)-C(64)	121.6(4)
C(59)-C(64)-C(63)	120.4(5)
N(9)-C(65)-C(62)	111.9(4)
C(67)-C(66)-C(71)	118.4(4)
C(67)-C(66)-C(54)	121.7(4)

C(71)-C(66)-C(54)	119.6(4)
C(66)-C(67)-C(68)	121.2(5)
C(69)-C(68)-C(67)	121.5(5)
C(68)-C(69)-C(70)	116.6(4)
C(68)-C(69)-C(72)	123.0(4)
C(70)-C(69)-C(72)	120.4(4)
C(71)-C(70)-C(69)	122.7(4)
C(70)-C(71)-C(66)	119.5(4)
N(10)-C(72)-C(69)	111.4(4)
C(78)-C(73)-C(74)	120.7(4)
C(78)-C(73)-C(79)	120.0(4)
C(74)-C(73)-C(79)	119.2(4)
C(75)-C(74)-C(73)	119.0(4)
C(75)-C(74)-C(80)	120.8(4)
C(73)-C(74)-C(80)	120.2(4)
C(76)-C(75)-C(74)	120.8(4)
C(76)-C(75)-C(82)	119.8(4)
C(74)-C(75)-C(82)	119.4(4)
C(75)-C(76)-C(77)	119.7(4)
C(75)-C(76)-C(83)	120.6(4)
C(77)-C(76)-C(83)	119.7(4)
C(78)-C(77)-C(76)	120.4(4)
C(78)-C(77)-C(85)	120.3(4)
C(76)-C(77)-C(85)	119.3(4)
C(77)-C(78)-C(73)	119.2(4)
C(77)-C(78)-C(86)	120.9(4)
C(73)-C(78)-C(86)	120.0(4)
N(3)-C(79)-C(73)	108.9(4)
C(74)-C(80)-C(81)	112.1(4)
N(9)-C(82)-C(75)	110.5(4)
C(76)-C(83)-C(84)	111.6(4)
N(7)-C(85)-C(77)	113.3(4)
C(78)-C(86)-C(87)	114.3(4)
C(89)-C(88)-C(93)	120.4(4)
C(89)-C(88)-C(94)	119.7(4)
C(93)-C(88)-C(94)	119.8(4)
C(88)-C(89)-C(90)	120.3(4)

C(88)-C(89)-C(95)	119.9(4)
C(90)-C(89)-C(95)	119.8(4)
C(89)-C(90)-C(91)	119.5(4)
C(89)-C(90)-C(97)	120.4(4)
C(91)-C(90)-C(97)	120.1(4)
C(92)-C(91)-C(90)	120.0(4)
C(92)-C(91)-C(98)	120.0(4)
C(90)-C(91)-C(98)	119.9(4)
C(91)-C(92)-C(93)	120.3(4)
C(91)-C(92)-C(100)	119.2(4)
C(93)-C(92)-C(100)	120.5(4)
C(92)-C(93)-C(88)	119.4(4)
C(92)-C(93)-C(101)	120.9(4)
C(88)-C(93)-C(101)	119.8(4)
N(4)-C(94)-C(88)	110.0(4)
C(89)-C(95)-C(96)	111.5(4)
N(10)-C(97)-C(90)	110.2(4)
C(91)-C(98)-C(99)	112.4(4)
N(8)-C(100)-C(92)	109.9(4)
C(93)-C(101)-C(102)	113.3(4)
N(11)-C(103)-C(104)	115.8(4)
C(106)-C(105)-N(11)	112.4(4)
N(11)-C(107)-C(108)	115.7(4)
N(12)-C(109)-C(110)	179.1(8)
N(13)-C(111)-C(112)	177.6(8)
N(14)-C(113)-C(114)	178.7(8)
N(15)-C(115)-C(116)	178.3(7)

Symmetry transformations used to generate equivalent atoms:

Table 3. Torsion angles [°] for SB_191220_2_MO.

C(2)-N(1)-C(1)-N(2)	0.0(5)
Fe(1)-N(1)-C(1)-N(2)	-178.3(3)
C(3)-N(2)-C(1)-N(1)	0.0(5)
C(4)-N(2)-C(1)-N(1)	175.5(4)
C(1)-N(1)-C(2)-C(3)	0.0(5)
Fe(1)-N(1)-C(2)-C(3)	178.1(3)
N(1)-C(2)-C(3)-N(2)	0.0(5)
C(1)-N(2)-C(3)-C(2)	0.0(5)
C(4)-N(2)-C(3)-C(2)	-175.1(4)
C(1)-N(2)-C(4)-C(5)	-163.5(4)
C(3)-N(2)-C(4)-C(5)	10.9(7)
N(2)-C(4)-C(5)-C(6)	-100.4(5)
N(2)-C(4)-C(5)-C(10)	87.6(5)
C(10)-C(5)-C(6)-C(7)	1.9(6)
C(4)-C(5)-C(6)-C(7)	-170.2(4)
C(10)-C(5)-C(6)-C(11)	-178.5(4)
C(4)-C(5)-C(6)-C(11)	9.4(6)
C(5)-C(6)-C(7)-C(8)	-1.8(7)
C(11)-C(6)-C(7)-C(8)	178.5(4)
C(6)-C(7)-C(8)-C(9)	0.1(7)
C(7)-C(8)-C(9)-C(10)	1.7(7)
C(8)-C(9)-C(10)-C(5)	-1.6(7)
C(8)-C(9)-C(10)-C(18)	176.4(4)
C(6)-C(5)-C(10)-C(9)	-0.1(6)
C(4)-C(5)-C(10)-C(9)	171.8(4)
C(6)-C(5)-C(10)-C(18)	-178.0(4)
C(4)-C(5)-C(10)-C(18)	-6.1(7)
C(7)-C(6)-C(11)-C(12)	71.9(6)
C(5)-C(6)-C(11)-C(12)	-107.7(5)
C(7)-C(6)-C(11)-C(16)	-107.0(5)
C(5)-C(6)-C(11)-C(16)	73.3(6)
C(16)-C(11)-C(12)-C(13)	-1.9(7)
C(6)-C(11)-C(12)-C(13)	179.2(4)
C(11)-C(12)-C(13)-C(14)	1.3(7)
C(12)-C(13)-C(14)-C(15)	0.5(7)
C(12)-C(13)-C(14)-C(17)	177.6(4)

C(13)-C(14)-C(15)-C(16)	-1.8(7)
C(17)-C(14)-C(15)-C(16)	-179.0(4)
C(14)-C(15)-C(16)-C(11)	1.3(7)
C(12)-C(11)-C(16)-C(15)	0.6(7)
C(6)-C(11)-C(16)-C(15)	179.5(4)
C(79)-N(3)-C(17)-C(14)	151.6(4)
C(13)-C(14)-C(17)-N(3)	152.1(4)
C(15)-C(14)-C(17)-N(3)	-31.0(6)
C(9)-C(10)-C(18)-C(23)	129.9(5)
C(5)-C(10)-C(18)-C(23)	-52.2(6)
C(9)-C(10)-C(18)-C(19)	-43.9(6)
C(5)-C(10)-C(18)-C(19)	134.0(5)
C(23)-C(18)-C(19)-C(20)	-1.5(6)
C(10)-C(18)-C(19)-C(20)	172.7(4)
C(18)-C(19)-C(20)-C(21)	0.1(7)
C(19)-C(20)-C(21)-C(22)	1.1(7)
C(19)-C(20)-C(21)-C(24)	-177.1(4)
C(20)-C(21)-C(22)-C(23)	-0.8(7)
C(24)-C(21)-C(22)-C(23)	177.4(4)
C(21)-C(22)-C(23)-C(18)	-0.6(7)
C(19)-C(18)-C(23)-C(22)	1.8(6)
C(10)-C(18)-C(23)-C(22)	-172.1(4)
C(94)-N(4)-C(24)-C(21)	-174.7(4)
C(22)-C(21)-C(24)-N(4)	14.2(7)
C(20)-C(21)-C(24)-N(4)	-167.7(4)
C(26)-N(5)-C(25)-N(6)	-1.4(6)
Fe(1)-N(5)-C(25)-N(6)	-156.6(4)
C(27)-N(6)-C(25)-N(5)	2.1(7)
C(28)-N(6)-C(25)-N(5)	-178.7(5)
C(25)-N(5)-C(26)-C(27)	0.1(6)
Fe(1)-N(5)-C(26)-C(27)	161.9(4)
N(5)-C(26)-C(27)-N(6)	1.1(6)
C(25)-N(6)-C(27)-C(26)	-1.9(6)
C(28)-N(6)-C(27)-C(26)	178.9(5)
C(25)-N(6)-C(28)-C(29)	-172.7(5)
C(27)-N(6)-C(28)-C(29)	6.3(7)
N(6)-C(28)-C(29)-C(34)	-79.5(5)

N(6)-C(28)-C(29)-C(30)	95.3(5)
C(34)-C(29)-C(30)-C(31)	-0.5(6)
C(28)-C(29)-C(30)-C(31)	-175.4(4)
C(34)-C(29)-C(30)-C(42)	178.0(4)
C(28)-C(29)-C(30)-C(42)	3.1(6)
C(29)-C(30)-C(31)-C(32)	-0.2(7)
C(42)-C(30)-C(31)-C(32)	-178.7(5)
C(30)-C(31)-C(32)-C(33)	0.5(8)
C(31)-C(32)-C(33)-C(34)	-0.2(7)
C(32)-C(33)-C(34)-C(29)	-0.5(7)
C(32)-C(33)-C(34)-C(35)	175.5(4)
C(30)-C(29)-C(34)-C(33)	0.9(6)
C(28)-C(29)-C(34)-C(33)	175.6(4)
C(30)-C(29)-C(34)-C(35)	-175.0(4)
C(28)-C(29)-C(34)-C(35)	-0.2(6)
C(33)-C(34)-C(35)-C(40)	-63.7(6)
C(29)-C(34)-C(35)-C(40)	112.2(5)
C(33)-C(34)-C(35)-C(36)	118.1(5)
C(29)-C(34)-C(35)-C(36)	-66.0(6)
C(40)-C(35)-C(36)-C(37)	-1.3(7)
C(34)-C(35)-C(36)-C(37)	177.1(4)
C(35)-C(36)-C(37)-C(38)	0.1(7)
C(36)-C(37)-C(38)-C(39)	1.0(7)
C(36)-C(37)-C(38)-C(41)	179.9(4)
C(37)-C(38)-C(39)-C(40)	-1.0(7)
C(41)-C(38)-C(39)-C(40)	-179.9(4)
C(38)-C(39)-C(40)-C(35)	-0.1(7)
C(36)-C(35)-C(40)-C(39)	1.2(7)
C(34)-C(35)-C(40)-C(39)	-177.1(4)
C(85)-N(7)-C(41)-C(38)	168.8(4)
C(37)-C(38)-C(41)-N(7)	134.3(5)
C(39)-C(38)-C(41)-N(7)	-46.8(6)
C(31)-C(30)-C(42)-C(43)	87.5(6)
C(29)-C(30)-C(42)-C(43)	-91.0(6)
C(31)-C(30)-C(42)-C(47)	-95.1(6)
C(29)-C(30)-C(42)-C(47)	86.4(6)
C(47)-C(42)-C(43)-C(44)	-2.6(8)

C(30)-C(42)-C(43)-C(44)	174.9(5)
C(42)-C(43)-C(44)-C(45)	0.1(8)
C(43)-C(44)-C(45)-C(46)	2.2(8)
C(43)-C(44)-C(45)-C(48)	-177.6(5)
C(44)-C(45)-C(46)-C(47)	-2.0(8)
C(48)-C(45)-C(46)-C(47)	177.8(5)
C(45)-C(46)-C(47)-C(42)	-0.5(8)
C(43)-C(42)-C(47)-C(46)	2.8(8)
C(30)-C(42)-C(47)-C(46)	-174.7(5)
C(100)-N(8)-C(48)-C(45)	-166.3(4)
C(46)-C(45)-C(48)-N(8)	-150.6(5)
C(44)-C(45)-C(48)-N(8)	29.1(7)
Fe(1)-O(2)-C(49)-O(1)	1.0(4)
Fe(1)-O(2)-C(49)-C(50)	-176.2(4)
Fe(1)-O(1)-C(49)-O(2)	-1.1(5)
Fe(1)-O(1)-C(49)-C(50)	176.1(4)
O(2)-C(49)-C(50)-C(51)	77.1(6)
O(1)-C(49)-C(50)-C(51)	-100.1(5)
C(49)-C(50)-C(51)-C(52)	53.6(5)
C(50)-C(51)-C(52)-C(53)	154.8(4)
C(51)-C(52)-C(53)-C(58)	-79.7(5)
C(51)-C(52)-C(53)-C(54)	104.5(5)
C(58)-C(53)-C(54)-C(55)	-2.8(6)
C(52)-C(53)-C(54)-C(55)	173.1(4)
C(58)-C(53)-C(54)-C(66)	175.8(4)
C(52)-C(53)-C(54)-C(66)	-8.3(7)
C(53)-C(54)-C(55)-C(56)	3.0(7)
C(66)-C(54)-C(55)-C(56)	-175.7(4)
C(54)-C(55)-C(56)-C(57)	-1.0(7)
C(55)-C(56)-C(57)-C(58)	-1.1(7)
C(56)-C(57)-C(58)-C(53)	1.3(7)
C(56)-C(57)-C(58)-C(59)	-175.9(4)
C(54)-C(53)-C(58)-C(57)	0.7(6)
C(52)-C(53)-C(58)-C(57)	-175.3(4)
C(54)-C(53)-C(58)-C(59)	177.7(4)
C(52)-C(53)-C(58)-C(59)	1.8(6)
C(57)-C(58)-C(59)-C(64)	-88.2(6)

C(53)-C(58)-C(59)-C(64)	94.7(6)
C(57)-C(58)-C(59)-C(60)	90.5(6)
C(53)-C(58)-C(59)-C(60)	-86.6(6)
C(64)-C(59)-C(60)-C(61)	-1.6(7)
C(58)-C(59)-C(60)-C(61)	179.6(5)
C(59)-C(60)-C(61)-C(62)	0.8(8)
C(60)-C(61)-C(62)-C(63)	0.0(7)
C(60)-C(61)-C(62)-C(65)	-179.2(5)
C(61)-C(62)-C(63)-C(64)	0.0(7)
C(65)-C(62)-C(63)-C(64)	179.2(5)
C(60)-C(59)-C(64)-C(63)	1.6(7)
C(58)-C(59)-C(64)-C(63)	-179.6(4)
C(62)-C(63)-C(64)-C(59)	-0.9(8)
C(82)-N(9)-C(65)-C(62)	-171.9(4)
C(63)-C(62)-C(65)-N(9)	-117.0(5)
C(61)-C(62)-C(65)-N(9)	62.2(6)
C(55)-C(54)-C(66)-C(67)	119.4(5)
C(53)-C(54)-C(66)-C(67)	-59.3(6)
C(55)-C(54)-C(66)-C(71)	-55.1(6)
C(53)-C(54)-C(66)-C(71)	126.3(5)
C(71)-C(66)-C(67)-C(68)	0.8(7)
C(54)-C(66)-C(67)-C(68)	-173.7(4)
C(66)-C(67)-C(68)-C(69)	0.2(8)
C(67)-C(68)-C(69)-C(70)	-2.1(7)
C(67)-C(68)-C(69)-C(72)	177.7(5)
C(68)-C(69)-C(70)-C(71)	3.1(7)
C(72)-C(69)-C(70)-C(71)	-176.7(4)
C(69)-C(70)-C(71)-C(66)	-2.2(8)
C(67)-C(66)-C(71)-C(70)	0.2(7)
C(54)-C(66)-C(71)-C(70)	174.8(4)
C(97)-N(10)-C(72)-C(69)	168.5(4)
C(68)-C(69)-C(72)-N(10)	98.5(6)
C(70)-C(69)-C(72)-N(10)	-81.7(6)
C(78)-C(73)-C(74)-C(75)	2.2(6)
C(79)-C(73)-C(74)-C(75)	178.5(4)
C(78)-C(73)-C(74)-C(80)	-178.1(4)
C(79)-C(73)-C(74)-C(80)	-1.8(6)

C(73)-C(74)-C(75)-C(76)	1.3(6)
C(80)-C(74)-C(75)-C(76)	-178.4(4)
C(73)-C(74)-C(75)-C(82)	-175.9(4)
C(80)-C(74)-C(75)-C(82)	4.4(6)
C(74)-C(75)-C(76)-C(77)	-1.8(6)
C(82)-C(75)-C(76)-C(77)	175.4(4)
C(74)-C(75)-C(76)-C(83)	175.8(4)
C(82)-C(75)-C(76)-C(83)	-7.0(6)
C(75)-C(76)-C(77)-C(78)	-1.4(6)
C(83)-C(76)-C(77)-C(78)	-179.0(4)
C(75)-C(76)-C(77)-C(85)	177.0(4)
C(83)-C(76)-C(77)-C(85)	-0.6(6)
C(76)-C(77)-C(78)-C(73)	4.9(6)
C(85)-C(77)-C(78)-C(73)	-173.5(4)
C(76)-C(77)-C(78)-C(86)	-174.7(4)
C(85)-C(77)-C(78)-C(86)	6.8(6)
C(74)-C(73)-C(78)-C(77)	-5.3(6)
C(79)-C(73)-C(78)-C(77)	178.5(4)
C(74)-C(73)-C(78)-C(86)	174.3(4)
C(79)-C(73)-C(78)-C(86)	-1.9(6)
C(17)-N(3)-C(79)-C(73)	-179.4(4)
C(78)-C(73)-C(79)-N(3)	83.6(5)
C(74)-C(73)-C(79)-N(3)	-92.7(5)
C(75)-C(74)-C(80)-C(81)	89.2(5)
C(73)-C(74)-C(80)-C(81)	-90.6(5)
C(65)-N(9)-C(82)-C(75)	-171.5(4)
C(76)-C(75)-C(82)-N(9)	-83.6(5)
C(74)-C(75)-C(82)-N(9)	93.6(5)
C(75)-C(76)-C(83)-C(84)	-86.1(6)
C(77)-C(76)-C(83)-C(84)	91.5(5)
C(41)-N(7)-C(85)-C(77)	-172.0(4)
C(78)-C(77)-C(85)-N(7)	-109.3(5)
C(76)-C(77)-C(85)-N(7)	72.2(6)
C(77)-C(78)-C(86)-C(87)	-87.8(5)
C(73)-C(78)-C(86)-C(87)	92.6(5)
C(93)-C(88)-C(89)-C(90)	-2.5(6)
C(94)-C(88)-C(89)-C(90)	-178.2(4)

C(93)-C(88)-C(89)-C(95)	180.0(4)
C(94)-C(88)-C(89)-C(95)	4.3(6)
C(88)-C(89)-C(90)-C(91)	1.0(6)
C(95)-C(89)-C(90)-C(91)	178.5(4)
C(88)-C(89)-C(90)-C(97)	179.2(4)
C(95)-C(89)-C(90)-C(97)	-3.3(6)
C(89)-C(90)-C(91)-C(92)	-0.4(6)
C(97)-C(90)-C(91)-C(92)	-178.5(4)
C(89)-C(90)-C(91)-C(98)	-179.4(4)
C(97)-C(90)-C(91)-C(98)	2.4(6)
C(90)-C(91)-C(92)-C(93)	1.2(6)
C(98)-C(91)-C(92)-C(93)	-179.8(4)
C(90)-C(91)-C(92)-C(100)	179.1(4)
C(98)-C(91)-C(92)-C(100)	-1.8(6)
C(91)-C(92)-C(93)-C(88)	-2.6(6)
C(100)-C(92)-C(93)-C(88)	179.5(4)
C(91)-C(92)-C(93)-C(101)	176.3(4)
C(100)-C(92)-C(93)-C(101)	-1.6(6)
C(89)-C(88)-C(93)-C(92)	3.3(6)
C(94)-C(88)-C(93)-C(92)	179.0(4)
C(89)-C(88)-C(93)-C(101)	-175.6(4)
C(94)-C(88)-C(93)-C(101)	0.1(6)
C(24)-N(4)-C(94)-C(88)	-177.9(4)
C(89)-C(88)-C(94)-N(4)	85.4(5)
C(93)-C(88)-C(94)-N(4)	-90.3(5)
C(88)-C(89)-C(95)-C(96)	87.3(5)
C(90)-C(89)-C(95)-C(96)	-90.2(5)
C(72)-N(10)-C(97)-C(90)	174.2(4)
C(89)-C(90)-C(97)-N(10)	-92.3(5)
C(91)-C(90)-C(97)-N(10)	85.8(5)
C(92)-C(91)-C(98)-C(99)	-89.6(5)
C(90)-C(91)-C(98)-C(99)	89.4(5)
C(48)-N(8)-C(100)-C(92)	178.0(4)
C(91)-C(92)-C(100)-N(8)	-75.5(5)
C(93)-C(92)-C(100)-N(8)	102.4(5)
C(92)-C(93)-C(101)-C(102)	90.4(5)
C(88)-C(93)-C(101)-C(102)	-90.7(5)

C(107)-N(11)-C(103)-C(104)	-56.5(6)
C(105)-N(11)-C(103)-C(104)	63.3(5)
Fe(1)-N(11)-C(103)-C(104)	178.6(4)
C(103)-N(11)-C(105)-C(106)	172.0(4)
C(107)-N(11)-C(105)-C(106)	-65.9(5)
Fe(1)-N(11)-C(105)-C(106)	58.6(5)
C(103)-N(11)-C(107)-C(108)	-48.7(6)
C(105)-N(11)-C(107)-C(108)	-169.3(4)
Fe(1)-N(11)-C(107)-C(108)	70.6(5)

Symmetry transformations used to generate equivalent atoms:

References

[1] S. C. Bete, C. Würtele, M. Otte, *Chem. Commun.* **2019**, *55*, 4427–4430.

[2] O. Francesconi, M. Gentili, C. Nativi, A. Ardá, F. Javier, Cañada, J. Jiménez-Barbero, S. Roelens, Chem. Eur. J. **2014**, *20*, 6081-6091

[3] X. Wang, J. Barbosa, P. Blomgren, M. C. Bremer, J. Chen, J. J. Crawford, W. Deng, L. Dong, C. Eigenbrot, S. Gallion, J. Hau, H. Hu, A. R. Johnson, A. Katewa, J. E. Kropf, S. H. Lee, L. Liu, J. W. Lubach, J. Macaluso, P. Maciejewski, S. A. Mitchell, D. F. Ortwine, J. DiPaolo, K. Reif, H. Scheerens, A. Schmittt, H. Wong, J.-M. Xiong, J. Xu, Z. Zhao, F. Zhou, K. S. Currie and W. B. Young, *ACS Med. Chem. Lett,* **2017**, *8*, 606.

[4] A. Vacca, C. Nativi, M. Cacciarini, R. Pergoli and S. J. Roelens, S. *J. Am. Chem. Soc.,* 2004, **126**, 16456.

[5] Hathaway, B. J.; Holah, D. G.; Underhill, A. E. *J. Chem. Soc*. **1962**, 2444

[6] Dortmund Data Bank, 2020, www.ddbst.com

[7] a) APEX3 v2016.9-0 (SAINT/SADABS/SHELXT/SHELXL), Bruker AXS Inc., Madison, WI, USA, **2016**. b) George M. Sheldrick, *Acta Cryst.,* **2015**, A71, 3-8. c) George M. Sheldrick, *Acta Cryst*., **2015**, C71, 3-8. d) George M. Sheldrick, *Acta Cryst*., **2008**, A64, 112-122.

Author Contributions

S.B.: Synthesis and Characterization of the presented compounds. Obtained single crystals of **Fe@2**. Wrote the manuscript and the ESI with M.O.

M.O.: Wrote the manuscript and the ESI with S.B.. Performed the crystallographic characterization of **Fe@2**. Is the project administrator and aquired funding for the project.
